# Supplementary material for: DNA diamond formulates a decomposable composite letter constellation model for DNA data storage
Source: Nat Commun. 2026 Jan 31;17:1704. doi: 10.1038/s41467-026-68861-y (PMC12909984; doi:10.1038/s41467-026-68861-y)
Supplement: Supplementary file 1 — Supplementary Information [file 41467_2026_68861_MOESM1_ESM.pdf]

Supporting Information

DNA Diamond Formulates a Decomposable Composite Letter Constellation Model for DNA Data Storage

Qi Ge[1], Menghui Ren[1], Tingting Qi[1], Changcai Han[1], Yingjin Yuan[2,3]✉, Weigang Chen[1,2,3]✉

[1]School of Microelectronics, Tianjin University, Tianjin, China.

[2]State Key Laboratory of Synthetic Biology, Tianjin University, Tianjin, China.

[3]Frontiers Science Center for Synthetic Biology (Ministry of Education), School of Synthetic Biology and Biomanufacturing, Tianjin University, Tianjin, China.

✉e-mail: yjyuan@tju.edu.cn; chenwg@tju.edu.cn

Supporting Information includes

Supplementary Notes

Supplementary Note 1: The workflow of encoding for composite letters and indices.
Supplementary Note 2: Calculation of information density.
Supplementary Note 3: Mapping efficiency between binary bits and composite letters.
Supplementary Note 4: The principles of information entropy for set partitioning of composite letters.
Supplementary Note 5: Set partitioning method for the composite letter detection.
Supplementary Note 6: Cost analysis of the proposed composite-letter DNA storage system.

Supplementary Figures

Supplementary Figure 1. Workflow of DNA storage using decomposable composite letter constellations.
Supplementary Figure 2. Experimental verification using column-based synthesis and simulation.
Supplementary Figure 3. Experimental verification using array-based DNA synthesis.
Supplementary Figure 4. Illustration of the composite strand synthesis with column-based and array-based synthesizers.
Supplementary Figure 5. Writing workflow of composite DNA data storage.
Supplementary Figure 6. Natural base representation of composite letters and composite DNA strands.
Supplementary Figure 7. Entropy values of different composite alphabets.
Supplementary Figure 8. Workflow of composite letter detection based on NGS paired-end reads.
Supplementary Figure 9. Preprocessing of paired-end sequencing reads.
Supplementary Figure 10. Encoding schemes for eight-letter composite letter DNA storage.
Supplementary Figure 11. Encoding schemes for 15-letter composite letter DNA storage.
Supplementary Figure 12. Encoding of eight-letter composite DNA storage (126 composite strands, R=1/3).
Supplementary Figure 13. Encoding of eight-letter composite DNA storage (126 composite strands, R=5/6).
Supplementary Figure 14. Encoding of the eight-letter composite DNA storage system (10,000 composite strands).
Supplementary Figure 15. Encoding of the 15-letter composite DNA storage system (10,000 composite strands).
Supplementary Figure 16. Encoding of the 15-letter composite DNA storage system (32,767 composite strands).
Supplementary Figure 17. Mapping efficiency of bits to composite letters.
Supplementary Figure 18. Bit-to-alphabet mapping efficiency as a function of bit group size.

Supplementary Figure 19. Base frequency distribution (1,000×, 126 composite oligos).
Supplementary Figure 20. Base frequency distribution (50×, 126 composite oligos).
Supplementary Figure 21. Base frequency distribution (10×, 126 composite oligos).
Supplementary Figure 22. Base frequency distribution (1,000×, 10,000 composite oligos, eight-letter alphabet).
Supplementary Figure 23. Base frequency distribution (19×, 10,000 composite oligos, eight-letter alphabet).
Supplementary Figure 24. Base frequency distribution (1,000×, 10,000 composite oligos, 15-letter alphabet).
Supplementary Figure 25. Base frequency distribution (33×, 10,000 composite oligos, 15-letter alphabet).
Supplementary Figure 26. Entropy distribution under practical synthesis and sequencing.
Supplementary Figure 27. Composite letter detection using a normalized maximum likelihood estimation method within subsets.
Supplementary Figure 28. Simulation model based on sequencing coverage, error rate, and random instantiation.
Supplementary Figure 29. The number of sequencing reads associated with each encoded composite strand at different coverages.
Supplementary Figure 30. Read count distribution of composite DNA strands at different average coverages (126 composite strands).
Supplementary Figure 31. Read count distributions at different sequencing coverages (10,000 composite strands).
Supplementary Figure 32. Read count distribution of 10,000 individual composite strands with a chosen eight-letter alphabet.
Supplementary Figure 33. Read count distribution of 10,000 individual composite strands with a chosen 15-letter alphabet.
Supplementary Figure 34. Error matrix of the detected composite letters based on a 15-letter alphabet at various sequencing coverages.
Supplementary Figure 35. Distance distribution between the 15 composite letters in the decomposable diamond model.
Supplementary Figure 36. Error analysis of the two-stage composite letter detection method based on experimental sequencing data.
Supplementary Figure 37. Substitution error matrices for the eight-letter Alphabet 1 {A, T, G, C, R, Y, M, K} at four sequencing coverages.
Supplementary Figure 38. Substitution error matrices for the eight-letter Alphabet 2 {A, T, R, Y, M, K, S, W} at four sequencing coverages.
Supplementary Figure 39. Substitution error matrices for the eight-letter Alphabet 3 {A, T, G, C, H, B, V, D} at four sequencing coverages.
Supplementary Figure 40. Error rates of different composite letters in experimental tests.
Supplementary Figure 41. Error rates of different letters in a 15-letter composite alphabet.
Supplementary Figure 42. End-to-end decoding performance of the eight-letter system (126 strands, 116 letters, RS code, R=5/6).
Supplementary Figure 43. End-to-end decoding performance of the eight-letter system

(126 strands, 116 letters, NB-LDPC code, R=1/3).
Supplementary Figure 44. End-to-end decoding performance of the eight-letter system (10,000 strands, 124 letters).
Supplementary Figure 45. End-to-end decoding performance of the eight-letter system (10,000 strands, 112 letters).
Supplementary Figure 46. End-to-end decoding performance of the 15-letter system (10,000 strands, 124 letters).
Supplementary Figure 47. End-to-end decoding performance of the 15-letter system (10,000 strands, 112 letters).
Supplementary Figure 48. Benchmarking the stochastic error model used in simulations.
Supplementary Figure 49. Coverage distribution in simulations.
Supplementary Figure 50. Simulated copy-number heterogeneity and pre-decoding error distribution for LDPC-protected composite strands.
Supplementary Figure 51. Simulated copy-number heterogeneity and pre-decoding error distribution for RS-protected composite strands.
Supplementary Figure 52. Data recovery tests at various sequencing coverages based on simulated sequencing reads for two pools using alphabet 1 {A, T, G, C, R, Y, M, K}.
Supplementary Figure 53. Data recovery performance for Alphabet 3 {A, T, G, C, H, B, V, D} using a non-interleaved scheme.
Supplementary Figure 54. Frequency-based inference of composite letters.
Supplementary Figure 55. Homopolymer length distribution.
Supplementary Figure 56. Length distribution of assembled sequencing reads.
Supplementary Figure 57. Length distributions of index and payload regions after primer identification.
Supplementary Figure 58. Readout workflow with length filtering.
Supplementary Figure 59. Error distribution in double-end indices.
Supplementary Figure 60. Design of composite DNA strand structure enabling degradation-tolerant decoding.
Supplementary Figure 61. Cumulative distributions of read length under different degradation conditions.
Supplementary Figure 62. Proportion of read length under different thermal degradation conditions (only payload).
Supplementary Figure 63. Magnified view of proportion of read length under different thermal degradation conditions (only payload).
Supplementary Figure 64. Classification of sequencing reads according to primers under different thermal degradation conditions (126 composite strands, 116 nt).
Supplementary Figure 65. Data recovery performance of degraded samples (126 composite strands, 116 nt).
Supplementary Figure 66. Recovery performance using severely-degraded sequencing reads.
Supplementary Figure 67. Letter detection accuracy and recovery performance with length filtering.
Supplementary Figure 68. Cost analysis of different DNA storage schemes.

Supplementary Tables

Supplementary Table 1. Comparison of published composite letter data storage schemes.

Supplementary Table 2. Composite letters and corresponding natural bases used in experimental proofs.

Supplementary Table 3. The number of ordinary strands corresponding to four composite DNA pools in this work.

Supplementary Table 4. Coding potential and the net information density.

Supplementary Table 5. Primers used in this work.

Supplementary Table 6. Unique index used in this work.

Supplementary Table 7. Letter detection error rates under different detection methods with an eight-letter alphabet {A, T, G, C, R, Y, M, K} and RS(1890, 1575).

Supplementary Table 8. Letter detection error rates under different detection methods with a 15-letter alphabet (10,000 composite strands, 124 letters).

Supplementary Table 9. Length filtering of the different experiments.

Supplementary Table 10. Comparative cost analysis aligned with prior studies.

Supplementary Table 11. Initial vectors and normalized probability vectors corresponding to the 11 composite letters.

Supplementary Table 12. PCR reaction components used in this work.

Supplementary Table 13. PCR reaction conditions.

Supplementary Table 14. Negative binomial distribution parameters for each fitted sequencing coverage.

Supplementary References

Supplementary Notes

Supplementary Note 1: The workflow of encoding for composite letters and indices.

Storing information using composite letters has been demonstrated to be an effective approach to enhancing the logical density of DNA storage systems while utilizing the same synthesis instruments and conditions[1,2]. Compared with conventional natural-base storage, composite letters can increase logical density by at least twofold[3–5]. However, composite-letter DNA storage faces challenges such as elevated error rates and the demand for high sequencing coverage[6]. To address these challenges, we developed a systematic encoding strategy and implemented double-end indices to improve data reliability[7].

1. Alphabet design and experimental overview

To evaluate the practicality of composite-letter DNA storage, we performed a series of experiments using eight-letter and 15-letter alphabets, implemented on two synthesis platforms: column-based and array-based DNA synthesis.

Specifically, three types of eight-letter alphabets were experimentally validated using column-based DNA synthesis. An eight-letter and a 15-letter alphabet were tested using array-based synthesis to assess the feasibility of large-scale composite-letter DNA storage.

1) Column-based synthesis of three eight-letter alphabets:

- **Alphabet 1 {A, T, G, C, R, Y, M, K}**. Composite letters were generated by mixing two natural bases in a 1:1 ratio, producing {R, Y, M, K}. Combined with the natural bases {A, T, G, C}, this formed an eight-letter model.
- **Alphabet 2 {A, T, R, Y, M, K, S, W}**. Composite letters {R, Y, M, K, S, W} generated by mixing two natural bases in a 1:1 ratio, combined with {A, T} to create an eight-letter model.
- **Alphabet 3 {A, T, G, C, H, B, V, D}.** Composite letters {H, B, V, D} were generated by mixing three natural bases in a 1:1:1 ratio. When combined with {A, T, G, C}, this yielded an eight-letter model.

2) Array-based synthesis of eight-letter and 15-letter alphabets:

- **Alphabet 1 {A, T, G, C, R, Y, M, K}.** Two pools containing 10,000 composite strands were designed and synthesized to validate the scalability of the eight-letter system.

- **Alphabet 4 {A, T, G, C, R, Y, S, W, M, K, H, B, V, D, N}.** A fifteen-letter composite alphabet, derived from the DNA diamond model, was implemented to further enhance logical density. Similarly, two 10,000-strand pools were designed using this alphabet to demonstrate the feasibility of large-alphabet DNA storage. Each composite letter was defined by specific mixture proportions of the four natural bases during synthesis, represented as normalized vectors of base composition (Supplementary Table 2).

2. Encoding workflow for small-scale oligo pools

This section describes the encoding of small-scale oligo pools (126 composite strands) used to verify the feasibility and robustness of the composite letter DNA storage. The overall workflow integrated error-correction coding with composite-letter mapping to ensure reliable digital-to-DNA conversion. Short, high-reliability codes were employed to encode the double-end indices, which were concatenated to both ends of each payload sequence before primer addition.

Non-binary low-density parity-check (NB-LDPC) codes defined over higher-order Galois fields (GF(q), ($q > 2$)) are known to outperform binary LDPC codes[8–10]. Their error-correction capability typically improves as code length increases. Building on this property, our system adopted NB-LDPC block codes for robust error correction. For comparison, a high-rate Reed–Solomon (RS) code was also evaluated. To examine robustness against burst erasures caused by low sequencing coverage, both randomly interleaved and non-interleaved NB-LDPC codewords were designed.

2.1 Encoding of payload sequences (composite letters)

High-reliability scheme:

1) The digital information (945 bytes) was first encoded using NB-LDPC(3780, 1260) code over $GF(2^6)$ to generate a 22,680-bit sequence.
2) The bit sequence was segmented into 7,560 chunks of 3 bits, each mapped to an octal symbol, producing 7,560 symbols.
3) The 7,560 symbols were arranged into 126 payload sequences, each consisting of 60 letters, corresponding to 126 composite DNA strands.

4) Three text files were encoded using the NB-LDPC scheme; a symbol-level random interleaver was applied to one codeword to disperse burst-erasure events and achieve error-free recovery at low coverage.

High-code-rate scheme:

For comparison, a high-code-rate RS(1890, 1575) code over $GF(2^{12})$ (code rate 0.83) was applied to a 2,355-byte text file, producing a 22,680-bit codeword. The same 3-bit-to-letter mapping was used, yielding 126 payload sequences of 60 letters.

Using the above NB-LDPC(3780, 1260) and RS(1890, 1575) coding schemes in combination with the three designed eight-letter alphabets, four column-based synthesis experiments were performed (Supplementary Figs. 2 and 10):

- **Experiment 1**: 126 strands encoded using randomly interleaved NB-LDPC(3780, 1260) and Alphabet 1 {A, T, G, C, R, Y, M, K}.
- **Experiment 2**: 126 strands encoded using high-code-rate RS(1890, 1575) and Alphabet 1 {A, T, G, C, R, Y, M, K}.
- **Experiment 3**: 126 strands encoded using non-interleaved NB-LDPC(3780, 1260) and Alphabet 2 {A, T, R, Y, M, K, S, W}.
- **Experiment 4**: 126 strands encoded using non-interleaved NB-LDPC(3780, 1260) and Alphabet 3 {A, T, G, C, H, B, V, D}.

2.2 Encoding of double-end indices

The encoding workflow for double-end indices follows these well-defined steps:

1) **Encoding of the index:** The index, represented as a 7-bit binary sequence, ranges from 1 to 126 and corresponds to the sequence order of the 126 composite strands. Each 7-bit binary address is encoded using a BCH(15, 7) error correction code, generating a 15-bit codeword. An additional parity bit is appended, resulting in a 16-bit encoded binary sequence.
2) **Mapping bit sequence to index DNA sequence (5' end):** The 16-bit encoded binary sequence is divided into groups of two bits and mapped to DNA bases according to the rule:

00→A, 01→T, 10→G, and 11→C. This conversion produces an 8-nt index DNA sequence, which was appended to the 5' end of the payload sequence.

3) **Generating reverse index DNA sequence:** A parity bit is generated based on the original 7-bit binary address, which is then cyclically left-shifted by one bit to form a new 7-bit sequence. The reverse index sequence follows the format: ["parity bit–original address bits–parity bit–left-shifted address bits"], yielding a 16-bit encoded reverse index sequence.
4) **Mapping bit sequence to index DNA sequence (3' end):** The 16-bit reverse index sequence is encoded into an 8-nt DNA sequence using the same mapping rule as in Step 2. This reverse index DNA sequence is appended to the 3' end of the payload sequence. To form an oligonucleotide pool, 20-nt primer sequences are added to the both end for amplification.

3. Encoding workflow for large-scale oligo pools

To further evaluate the scalability of composite-letter DNA storage, four large-scale experiments were conducted using an eight-letter alphabet and a 15-letter alphabet. Two index schemes were designed for each oligo pool.

3.1 Encoding of payload sequences (composite letters)

Eight-letter composite letter DNA storage system:

1) A digital file of 187,500 bytes was segmented into 40 data blocks of 37,500 bits.
2) Each block contained 3,125 information symbols (12 bits each) and was encoded using RS(3750, 3125) code over $GF(2^{12})$, adding 625 redundant symbols for error correction.
3) Each 12-bit RS symbol was mapped into four octal symbols, and every 3 bits were mapped into one composite letter from the alphabet {A, T, G, C, R, Y, M, K}.
4) The encoded payload consisted of 15,000 octal symbols, corresponding to 250 strands of 60 letters each. Across 40 blocks, a total of 10,000 strands were generated.

15-letter composite letter DNA storage system:

1) An image file of 234,375 bytes was divided into 5 data blocks of 375,000 bits.
2) Each block comprised 25,000 information symbols of 15 bits and was encoded using

RS(30000, 25000) code over $GF(2^{15})$, adding 5,000 redundant symbols.

3) Each 15-bit symbol was mapped into four base-15 symbols, and every 15 bits were mapped into four composite letters from the alphabet {A, T, G, C, R, Y, S, W, M, K, H, B, V, D, N}.
4) The encoded payload consisted of 120,000 symbols, corresponding to 2,000 strands of 60 letters each. Across 5 blocks, a total of 10,000 strands were generated.

3.2 Index encoding schemes for large-scale composite strands

Two index schemes were designed for both the eight-letter and 15-letter systems.

1) Scheme 1 (independent 12-nt indices at both ends, 124 nt total length).

A 14-bit binary address was assigned to each sequence (sufficient to index up to 10,000 strands). This address was encoded using a shortened BCH code, BCH(24, 14), derived from BCH(31, 21), producing a 12-nt index. The forward (5') index was generated directly from the 14-bit address, whereas the reverse (3') index was produced by randomly interleaving the 14-bit address and applying the same BCH(24, 14) code, resulting in another 12-nt sequence. Together with 20-nt primers at both ends, the oligonucleotide length was 124 nt.

2) Scheme 2 (independent 6-nt indices at both ends, 112 nt total length).

A 14-bit address was similarly encoded using BCH(24, 14) code to produce a 12-nt index, which was then divided into two 6-nt halves. The first 6 nt were placed at the 5' end and the remaining 6 nt at the 3' end. With 20-nt primers at both ends, the final oligonucleotide length was 112 nt.

Supplementary Note 2: Calculation of information density.

In natural-base DNA storage systems, information density is usually measured in bits per nucleotide (bits/nt). In composite-letter systems such as the DNA diamond, each synthesis cycle corresponds to one composite letter. Therefore, bits per letter (bits/letter) is a more proper metric for describing the information density. The theoretical coding potential of an alphabet with K letters is defined as: $\log_2(K)$ bits/letter[1,2,6]. In practice, the entire storage process must account for reductions in information density caused by indices, PCR primers, and error

correction codes. Accordingly, when describing the information density of composite alphabets, we define the information density[1,6] (excluding indices and primers) as:

$$Information\ density = \frac{\text{Stored data size (bits)}}{\text{Total number of composite letters}}.$$

Taking into account the coding scheme, indices, and primers, the net information density can be calculated as:

$$Net\ information\ density = \frac{\text{Stored data size (bits)}}{\text{The length of DNA strand * strand count}}.$$

For the eight-letter DNA storage system, the theoretical coding potential is $\log_2(8) = 3$ bits/letter assuming no overhead from encoding, indices, and primers. In the simulation using a 15-letter alphabet, the theoretical coding upper bound increases to $\log_2(15) \approx 3.9$ bits/letter.

Small-scale validation using column-based DNA synthesis

In our small-scale experiments (Supplementary Fig. 2), we synthesized 126 composite strands per experiment, each containing 60 payload letters plus 56 nt of primers and indices (total 116 nt). With 126 strands, the total number of letters is 14,616.

Two encoding schemes were adopted:

1) **High-reliability scheme:**

The user data were encoded using NB-LDPC(3780, 1260) over $GF(2^6)$, with a code rate of 1/3. The payload logical density was 1 bit/letter. Considering all synthesized nucleotides, the net information density was 0.52 bits/letter.

2) **High-code-rate scheme:**

The user data were encoded using RS(1890, 1575), with a code rate of 0.83. The payload logical density was 2.5 bits/letter, and the corresponding net information density was 1.29 bits/letter.

In computational simulation using the 15-letter alphabet, the achieved payload logical density was 3.18 bits/letter. As this simulation excluded primers and index regions, we focused on the density of the payload without net density calculations.

Large-scale validation using array-based inkjet DNA synthesis

We designed and synthesized four large pools using low-cost array-based inkjet phosphoramidite chemistry (Supplementary Fig. 3). In this new design, the number of composite strands was increased to 10,000 strands.

(i) **Two experiments with the eight-letter alphabet:** The payload logical density was 2.5 bits/letter. The net information density was 1.21 bits/letter (124 letters including primers and indices) and 1.34 bits/letter (112 letters including primers and index).

(ii) **Experiment with the 15-letter alphabet:** The payload logical density was 3.125 bits/letter, and the corresponding net information density was 1.51 bits/letter (124 letters per DNA strand) and 1.67 bits/letter (112 letters per DNA strand).

Supplementary Note 3: Mapping efficiency between binary bits and composite letters.

To evaluate the information density of composite alphabets, we introduce the concept of mapping efficiency, defined as the ratio between the actual number of binary bits stored and the theoretical maximum capacity of the composite letters used. This metric enables us to quantify the trade-off between storage efficiency and error propagation when mapping binary data into composite letters.

During the mapping from binary data to composite letters, the binary data are divided into blocks of size n bits, each with an information capacity of $\log_2(2)\times n$. These blocks are mapped to composite letters from an alphabet of size k, requiring $\lceil n/\log_2(k)\rceil$ composite letters per block, with an information capacity of $\log_2(k)\times\lceil n/\log_2(k)\rceil$. Thus, η can be expressed as:

$$\eta = \frac{\log_2(2)\times n}{(\log_2(k)\times\lceil n/\log_2(k)\rceil)},$$

where n is the size of the binary data block (in bits), k is the size of the composite alphabet (e.g., $k=8$ or $k=15$), and $\lceil \bullet \rceil$ denotes the ceiling function to calculate the number of composite letters required to represent n bits of data.

We investigated the impact of block size (n) on the coding potential for both the eight-

letter and 15-letter composite systems (Supplementary Fig. 18).

(1) For the eight-letter composite system, the theoretical density is calculated as $\log_2(8) = 3$ bits per letter. It means that each 3-bit data block can be directly mapped to a single composite letter with a mapping efficiency of 100%. In practice, any encoding scheme with a block length that is an integer multiple of 3 bits can also be adopted.

(2) For the 15-letter composite system, the theoretical density is $\log_2(15) \approx 3.9$ bits/letter. When we choose $n = 15$, the mapping efficiency is 0.96, corresponding to 3.75 bits/letter. Using a longer block size can further improve the mapping efficiency (e.g., $n = 31$, efficiency = 0.99), but this would require eight scomposite letters per block. We selected the 15-bit block configuration in our study, as it offers an optimal trade-off between coding efficiency and error correction.

In summary, the mapping efficiency is determined by both the size of the composite alphabet and the chosen block size. For the eight-letter alphabet, three binary bits can be directly mapped to one letter, yielding an efficiency of 1.0. For the 15-letter alphabet, a 15-bit block mapped to four letters achieves an efficiency of 0.96. Larger block sizes can further improve efficiency but result in greater error propagation. Therefore, our choice of a 15-bit block represents an optimal configuration in terms of both efficiency and robustness.

Supplementary Note 4: The principles of information entropy for set partitioning of composite letters.

Entropy is a fundamental metric for quantifying randomness in sequencing data. *Naef et al.* proposed an entropy-based base-calling approach that assigns weighted significance to low-quality data during sequence alignment, clustering, and assembly, improving both efficiency and accuracy[11]. Low-entropy regions indicate high certainty and reliable base identification, whereas high-entropy regions suggest sequencing noise and ambiguity.

We introduced the concept of information entropy into composite letter DNA storage. Unlike previous methods that constructed composite letter alphabets based on the resolution parameter, our proposed approach ensures that the selected composite letters share only several

discrete entropy values. Specifically, we chose 15 composite letters with the discrete entropy values, corresponding to the vertices, edges, faces, and centroid of our proposed decomposable diamond constellation model. These composite letters exhibit only four distinct entropy values with well-separated distances. During data readout, the observed base frequencies are used to compute the entropy, which then determines the subset to which a composite letter belongs. This reduces the number of candidate letters from a 15-letter subset to an even smaller subset, simplifying the detection process. By leveraging entropy constraints, this method effectively narrows the search space, thereby reducing the complexity of composite letter detection.

Supplementary Note 5: Set partitioning method for the composite letter detection.

The principles of the set partitioning method

In communication systems, set partitioning is a critical technique in the design of coded modulation schemes[12,13]. By dividing the symbol constellation into smaller subsets with increasing minimum distance within each subset, the set partitioning method effectively reduces the composite letter detection error rates. This technique plays an essential role in optimizing composite letter detection processes by leveraging the distinguishable information entropy of different composite letter subsets.

Application of set partitioning in composite letter detection

We integrate the set partitioning method into a composite letter detection framework tailored for DNA data storage. Our encoding scheme utilizes 15 decomposable constellations, comprising four natural bases {A, T, G, C} and 11 composite letters. These letters represent mixtures of natural bases in specific proportions and are categorized into four subsets to optimize the detection and decoding process:

- **Subset 1**: Natural bases {A, T, G, C}, characterized by high specificity and low entropy in sequencing reads.
- **Subset 2**: Composite letters {R, Y, M, K, S, W}, which are equimolar mixtures of two natural bases.

- **Subset 3**: Composite letters {H, B, V, D}, formed by equimolar mixtures of three natural bases.
- **Subset 4:** Composite letter 'N', an equimolar mixture of all four natural bases.

These decomposable constellations enable a two-stage letter detection approach. In the first step, composite letters are partitioned into different subsets using set partitioning, and in the second step, precise detection is performed within each subset. This method progressively narrows the candidate base space during data readout, reducing the complexity of composite letter detection.

Entropy-based partitioning of composite letters

During the data readout process, the observed frequency of the four natural bases is used to compute information entropy for each composite letter. Entropy values serve as a discriminative metric for subset classification, significantly reducing the candidate search space:

- **Subset 1 detection**: If entropy is below 0.5, the letter is classified into a natural base set {A, T, G, C}, reducing the candidate space from 15 to 4 letters.
- **Subset 2 detection**: Letters in subset 2, consisting of two natural bases, exhibit entropy values close to 1. For instance, R (A, G) or Y (C, T) yield an equal probability distribution of their constituent bases. This confines the candidate space to {R, Y, M, K, S, W}, reducing it to 6 letters.
- **Subset 3 detection**: Letters in subset 3, composed of three natural bases (e.g., H = {A, C, T} or D = {A, G, T}), exhibit entropy values around 1.58 due to the equal contribution of three components, limiting the candidate space to {H, B, V, D}.
- **Subset 4 detection:** Letter in subset 4, comprising all four natural bases (N = {A, T, G, C}), exhibit an entropy value of approximately 2.0 due to equal base contributions.

This entropy-based coarse-grained detection strategy ensures that the decoding process operates within progressively smaller subsets, improving efficiency and accuracy.

Likelihood-based composite letter detection

Once a letter is grouped into a specific letter set, a more precise detection is performed using a maximum likelihood estimation (MLE) approach. Within each subset, the observed base frequency is compared against the theoretical frequency vectors of each candidate letter. For example, a letter in subset 2, such as R (A, G), should have observed frequencies of approximately 50% A and 50% G, while a letter in subset 3, such as H (A, C, T), should have nearly equal proportions of A, C, and T.

The likelihood function for each candidate letter is computed based on the observed frequencies, and the candidate with the highest likelihood is selected. This step ensures that even minor variations in sequencing reads caused by noise or errors are robustly handled.

Supplementary Note 6: Cost analysis of the proposed composite-letter DNA storage system.

One of the primary motivations for adopting composite alphabets in DNA storage is to reduce the number of synthesis cycles, which remain the dominant contributor to the total system cost[1,2]. To assess the practical benefits of our proposed eight-letter system and 15-letter system, we conducted a comparative cost analysis aligned with prior studies. The analysis considers three aspects: (1) synthesis and sequencing cost projections, and (2) normalized cost under varying synthesis-to-sequencing cost ratios.

Comparison of synthesis and sequencing costs for DNA data storage

Logical density and minimum coverage required for reliable decoding are key determinants of total cost. To ensure a fair comparison across studies with different encoding schemes, we projected the number of synthesized nucleotides required to store 1 MB of data based on the reported logical density of each method. The synthesis cost was calculated as the product of the total number of synthesized nucleotides in a single synthesis run and the unit cost per synthesized base. The sequencing cost was estimated as the product of the total number of sequenced bases required for error-free recovery and the unit sequencing cost. The number of required sequencing bases is proportional to the sequencing coverage necessary for complete data recovery.

Following prior work[3, 14], we assumed a sequencing cost of \$0.0000012/100 nt and a synthesis cost of \$0.05/100 nt. To account for indexing and amplification overhead, we considered both "only payload" and "payload and indices" cases (Supplementary Fig. 68). Under these assumptions, the synthesis-to-sequencing cost ratio can be as high as 40,000:1, making synthesis the dominant contributor to the total cost.

When the payload and index regions are considered, the cost of storing 1 MB of data is \$3,813 for the four-letter system (Organick et al.), \$2,140 for the six-letter system of Anavy et al., \$1,142 for the 15-letter system of Anavy et al., \$2,097 for the six-letter system of Choi et al., \$1,244 for the 15-letter system of Choi et al., \$2,036 for the six-letter system of Xu et al., and \$2,016 and \$1,613 for our eight-letter and 15-letter systems, respectively. Therefore, compared with the conventional four-letter system, the schemes of Anavy et al., Choi et al., Xu et al., and our proposed schemes all reduce synthesis cost. Based on the eight-letter scheme, the synthesis cost is reduced by ~45%. For the 15-letter systems, the designs of Anavy et al. and Choi et al. reduce synthesis cost by ~70% and ~67%, respectively, while our 15-letter design achieves a ~58% reduction. Under a like-for-like comparison within the same alphabet size, our eight-letter system attains a similar synthesis cost to prior six-letter systems, because our use of robust double-ended indices slightly lowers the net information density. Compared with the 15-letter systems of Anavy et al. and Choi et al., our 15-letter system does not further reduce cost. This is mainly because prior 15-letter designs used relatively small composite pools and thus short indices, whereas our pool size is 10,000 strands. As the data scale grows, index overhead occupies a larger fraction of each strand, leading to additional synthesis cost.

Despite the higher sequencing coverage required for composite letters, for example, our system achieves reliable data recovery at 16× coverage for the eight-letter system and 35× coverage for the 15-letter system. Since sequencing cost is orders of magnitude lower than synthesis cost, the overall readout cost remains lower than that of conventional schemes.

Normalized cost under varying synthesis-to-sequencing cost ratios

We further performed a normalized cost analysis under varying synthesis to sequencing

cost ratios ($C_{\text{syn}} : C_{\text{seq}}$), acknowledging that this ratio may decrease as synthesis technologies mature[1,6]. For comparative purposes, we assumed representative $C_{\text{syn}} : C_{\text{seq}}$ ratios of 1000:1 or 500:1. We used the standard four-letter design from Organick et al., which required 5× sequencing depth, as a cost normalization baseline.

The overall cost of storing data volume D bits under a given synthesis-to-sequencing cost ratio can be expressed as:

$$\text{Amortized Cost} = (D/l)C_{\text{syn}} + (D/l)C_{\text{seq}} \times m = (D/l)(C_{\text{syn}} + C_{\text{seq}} m),$$

where the l is the logical density (the stored information bits per synthesized nucleotide), m is the minimum sequencing coverage required for data recovery. Using this formulation, we then scaled the total cost of each scheme relative to this benchmark (Supplementary Table 10). Under these assumptions, and considering the "payload and indices" case (excluding primers). The results indicated that, the total cost was reduced by 47% for the eight-letter system and by 56% for the 15-letter system at a synthesis-to-sequencing cost ratio of 1000:1. When the ratio decreased to 500:1, the total cost reductions were 46% and 55% for the eight-letter and 15-letter systems, respectively.

Supplementary Figures

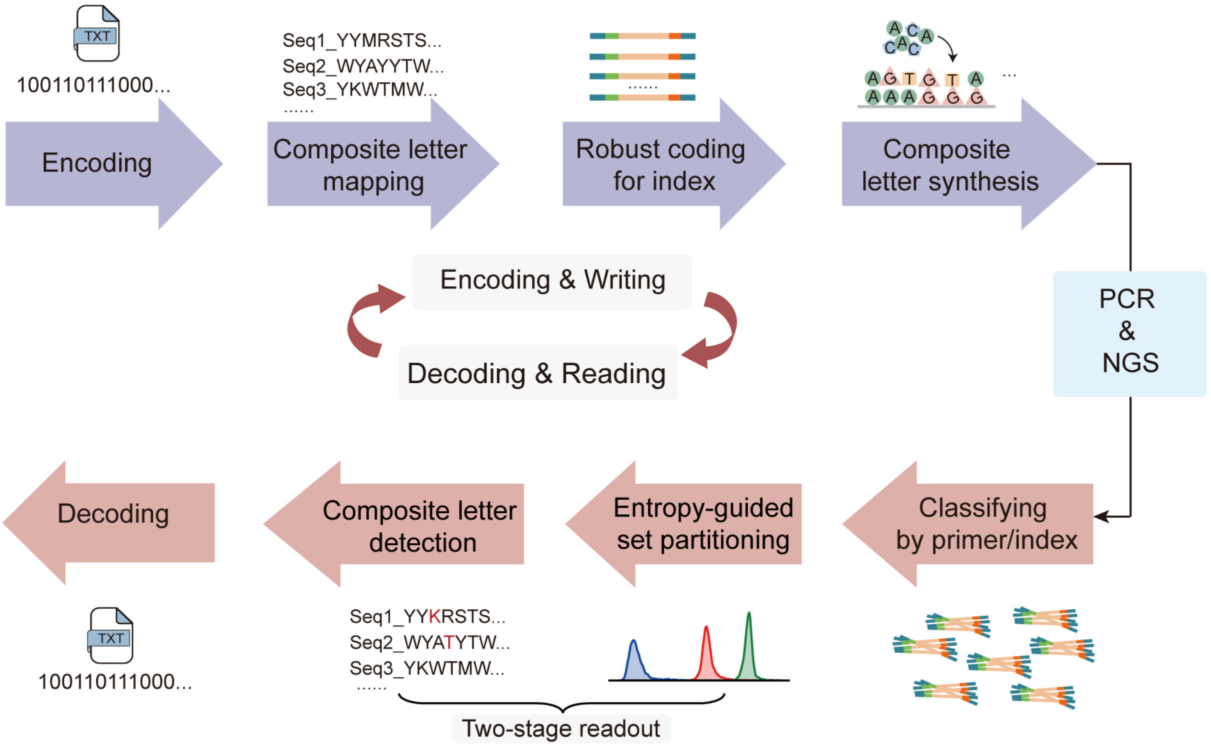

Supplementary Figure 1. Workflow of DNA storage using decomposable composite letter constellations. The proposed composite DNA storage system consists of two fundamental steps: data encoding and data readout. Reliable coding and robust double-end indices are designed to improve the reliability of read identification, particularly at low sequencing coverages. The readout process includes NGS paired-end read preprocessing, such as primer identification and double-end index identification, followed by composite letter detection via set partitioning.

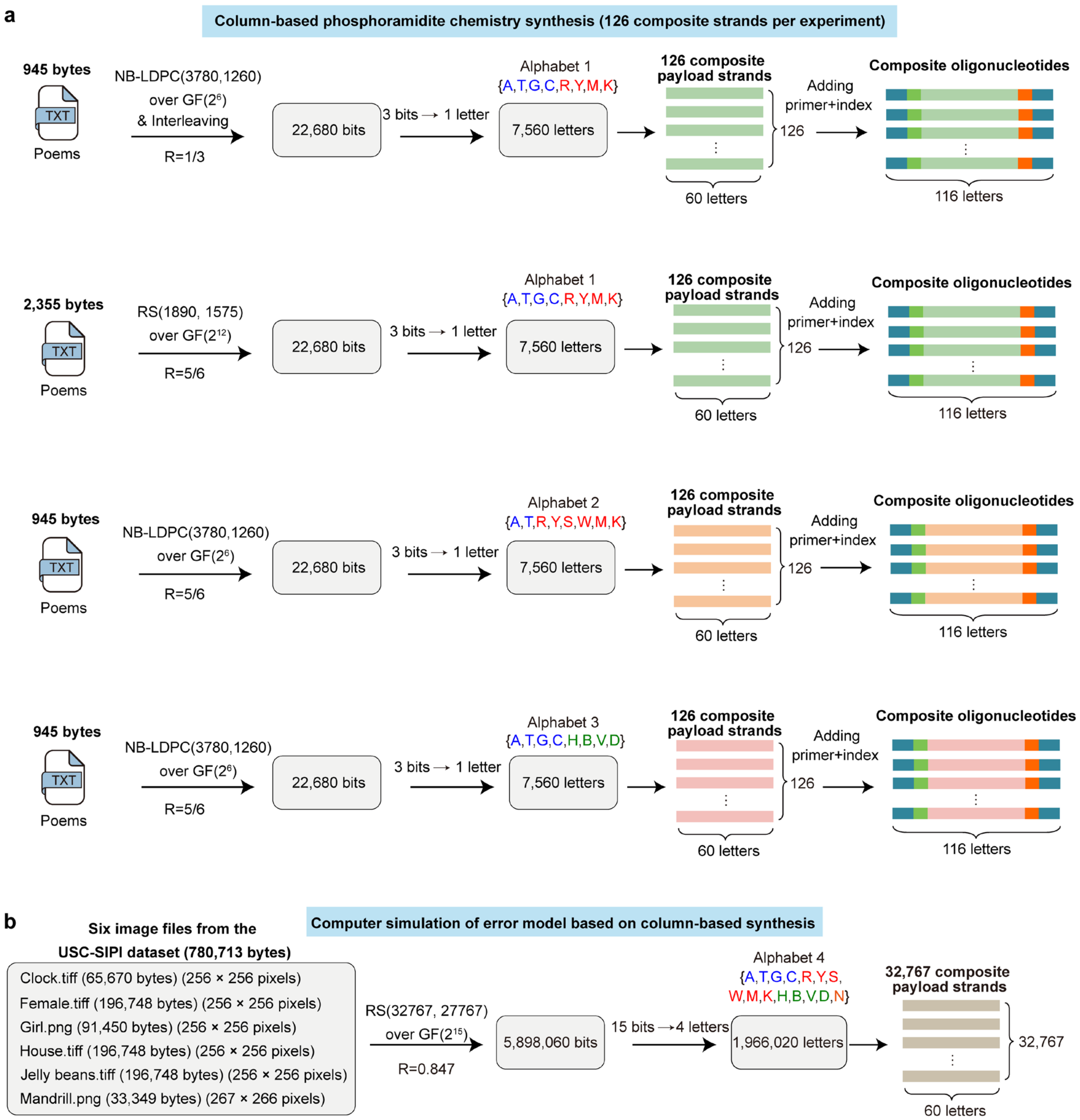

Supplementary Figure 2. Experimental verification using column-based synthesis and simulation. a, Four independent experiments were performed using column-based phosphoramidite chemistry. Two code rates, 1/3 and 5/6, were employed in combination with three distinct eight-letter alphabet schemes. In each experiment, 126 designed sequences with a payload of 60 composite letters and a total length of 116 nucleotides were synthesized. Each of the 126 sequences was individually synthesized in a separate column well. **b**, Computer simulation using six image files from the University of Southern California-Signal and Image Processing Institute (USC-SIPI) database (http://sipi.usc.edu/database/) (total 780,713 bytes). Data were encoded with an RS code (R=0.847), mapped to the 15-letter alphabet (15-bit to 4-letter) and formed 32,767 composite payload strands of 60 letters.

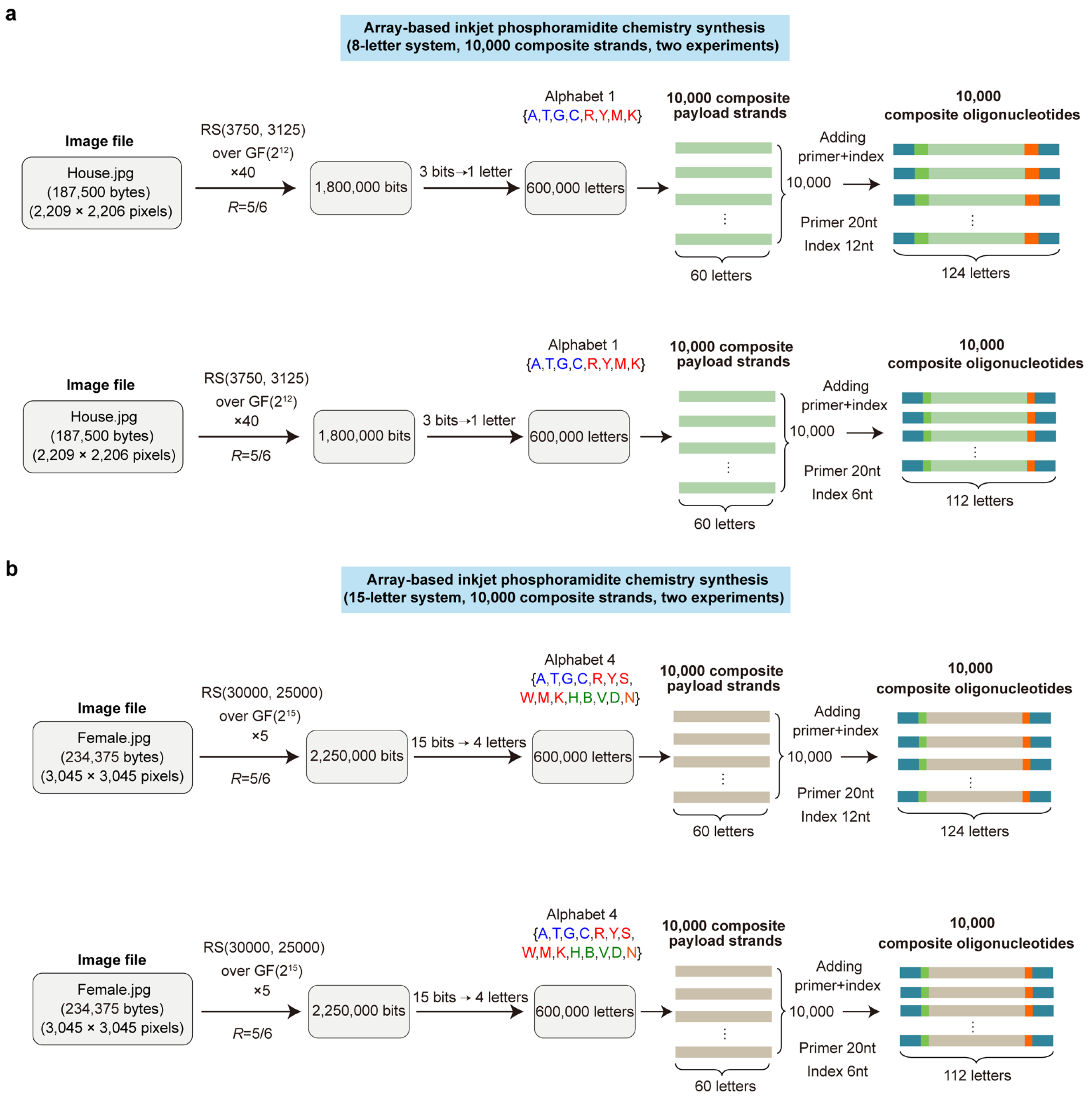

Supplementary Figure 3. Experimental verification using array-based DNA synthesis. a, Two composite oligo pools based on the eight-letter alphabet were synthesized using low-cost array-based inkjet phosphoramidite chemistry. Each experiment encoded a 187,500-byte image file into 600,000 composite letters, segmented into 10,000 strands of 60 letters. After adding 20-nt primers and indices of 12 nt or 6 nt, the resulting oligonucleotides were 124 letters and 112 letters, respectively. **b,** Two composite oligo pools based on the 15-letter alphabet were synthesized. Each experiment encoded a 234,375-byte image file into 600,000 composite letters, segmented into 10,000 strands of 60 letters. All four pools were synthesized using array-based inkjet DNA synthesis. The selected images (House.jpg and Female.jpg) are from USC-SIPI dataset (http://sipi.usc.edu/database/).

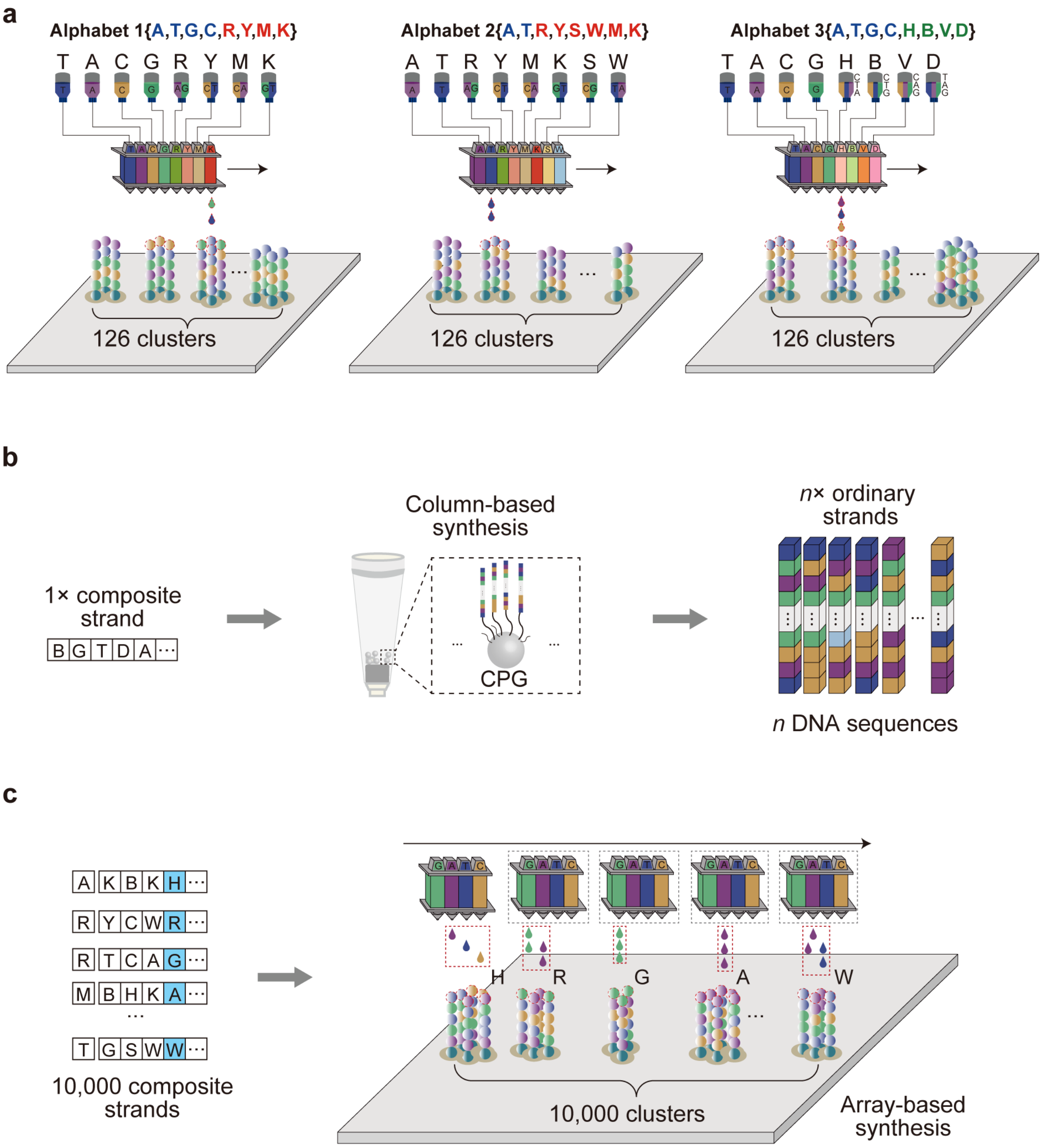

Supplementary Figure 4. Illustration of the composite strand synthesis with column-based and array-based synthesizers. a, Column-based synthesis of composite DNA strands. Eight reagent channels correspond to eight composite bases. Three composite alphabets were chosen and 126 composite strands were synthesized for each alphabet. **b**, Each designed composite strand, composed of composite letters (e.g., B, G, T, D, A, etc.), is individually synthesized in a dedicated well using column-based DNA synthesis. **c**, Array-based inkjet synthesis of large-scale composite-letter pools. Four printheads deliver monomer droplets onto array chips following predefined base mixing ratios.

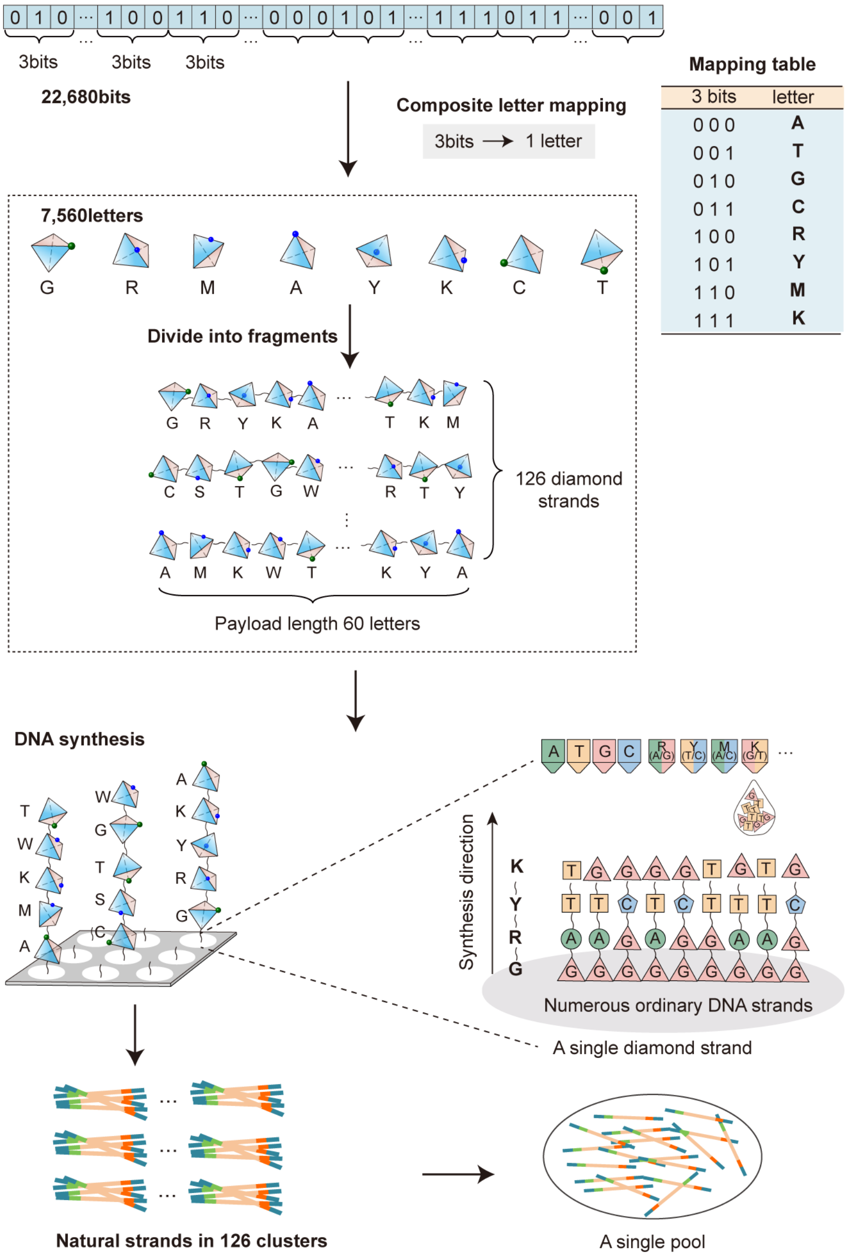

Supplementary Figure 5. Writing workflow of composite DNA data storage. First, the encoded bits are mapped to composite DNA letters (e.g., mapping 3 bits to 1 letter), where each composite letter represents a predefined mixture of nucleotides (e.g., A, T, G, C, R, Y, M, K). Second, 126 composite strands with a payload length of 60 letters are generated. Third, these sequences are synthesized in parallel using column-based phosphoramidite chemistry, in which different nucleotide mixtures are deposited in each synthesis cycle. Finally, ordinary DNA strands are produced, forming clusters that reflect the designed composite letters.

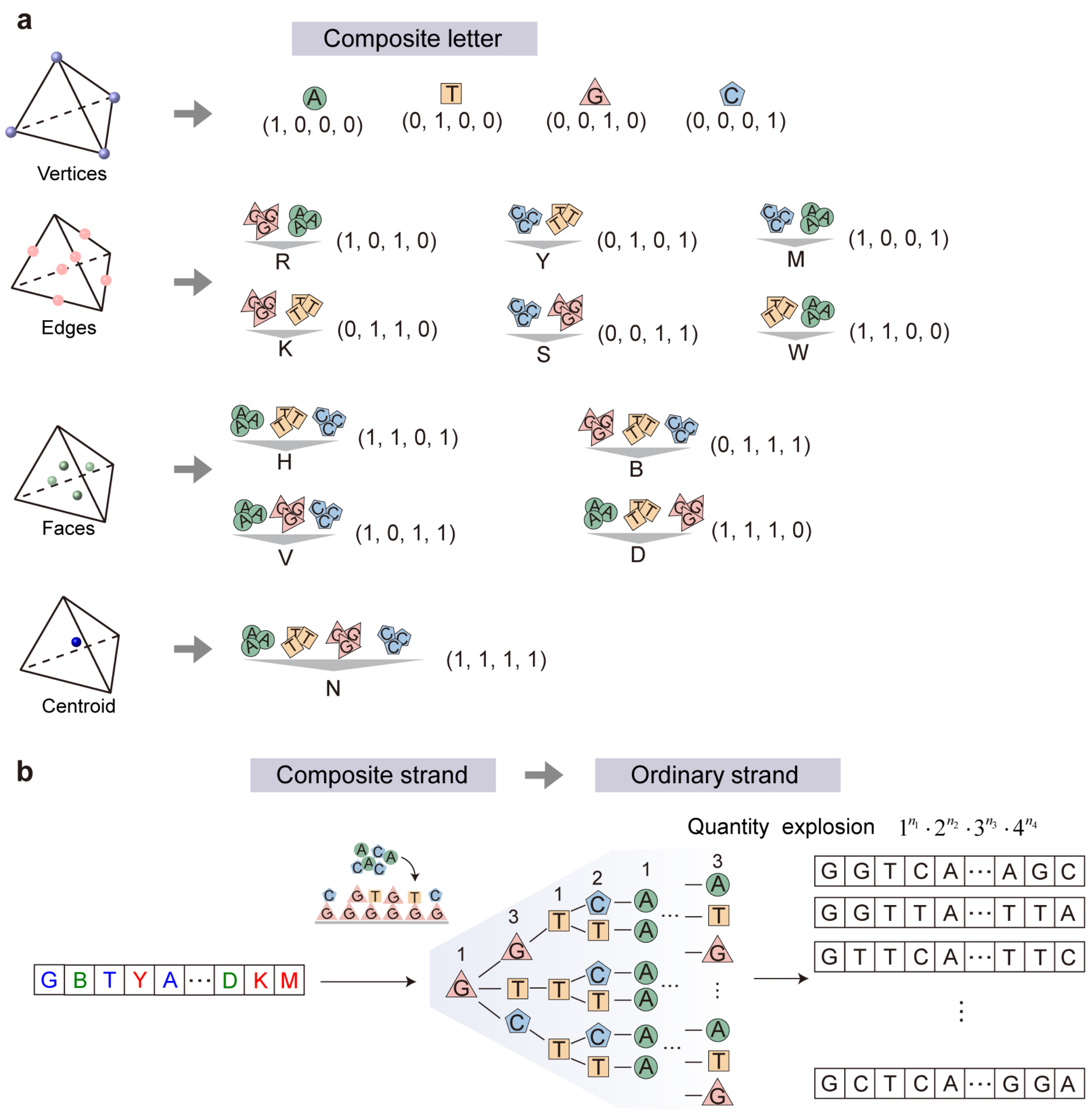

Supplementary Figure 6. Natural base representation of composite letters and composite DNA strands. a, The 15-letter composite alphabet includes four natural bases and eleven composite letters, each composite letter formed by controlled mixtures of the four natural bases. These letters correspond to vertices, edges, faces, and the centroid of a decomposable diamond constellation model. **b**, A strand written with these composite letters generates a combinatorial family of ordinary strands after DNA synthesis. The total number of strands expands as $1^{n_1}\cdot 2^{n_2}\cdot 3^{n_3}\cdot 4^{n_4}$, where n_1, n_2, n_3, and n_4 are the counts of natural bases, letters in the set {R, Y, M, K, S, W}, letters in the set {H, B, V, D}, and the letter 'N', respectively.

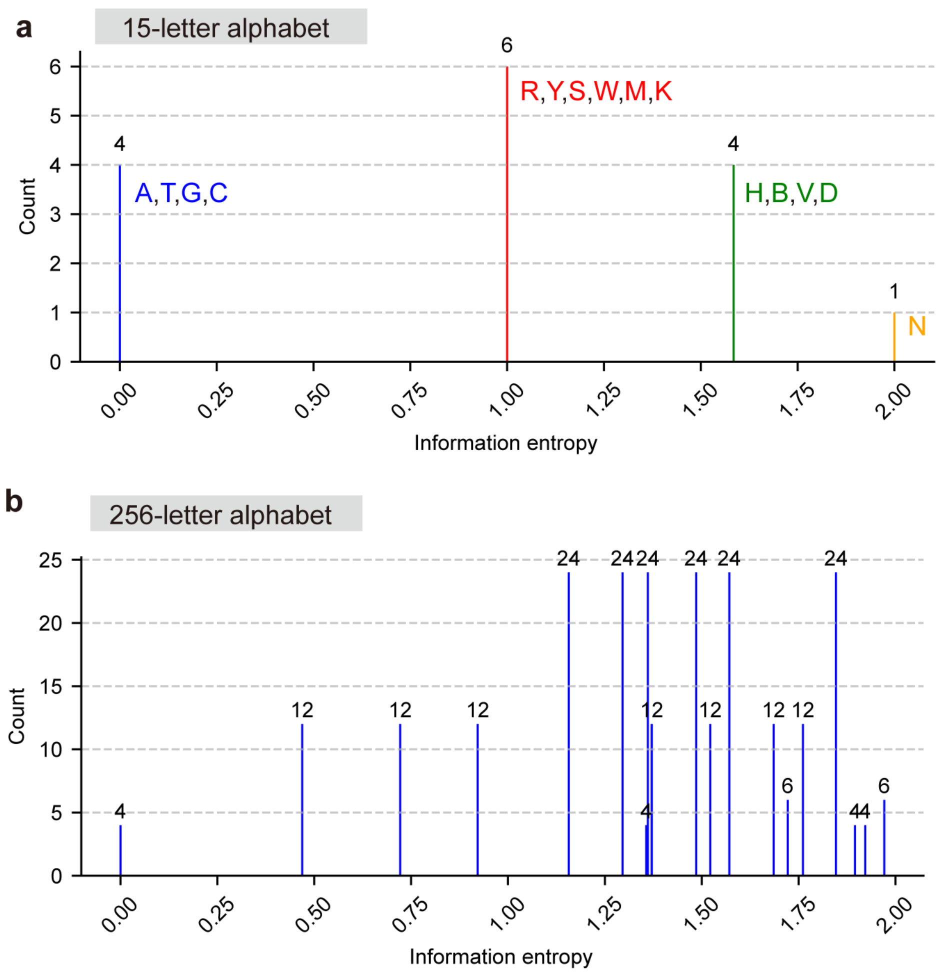

Supplementary Figure 7. Entropy values of different composite alphabets. a, Information entropy values of the 15 letters used in this study, exhibiting only four discrete entropy values. **b**, The discrete entropy values of a 256-letter composite alphabet with a resolution of 10. Source data are provided as a Source Data file.

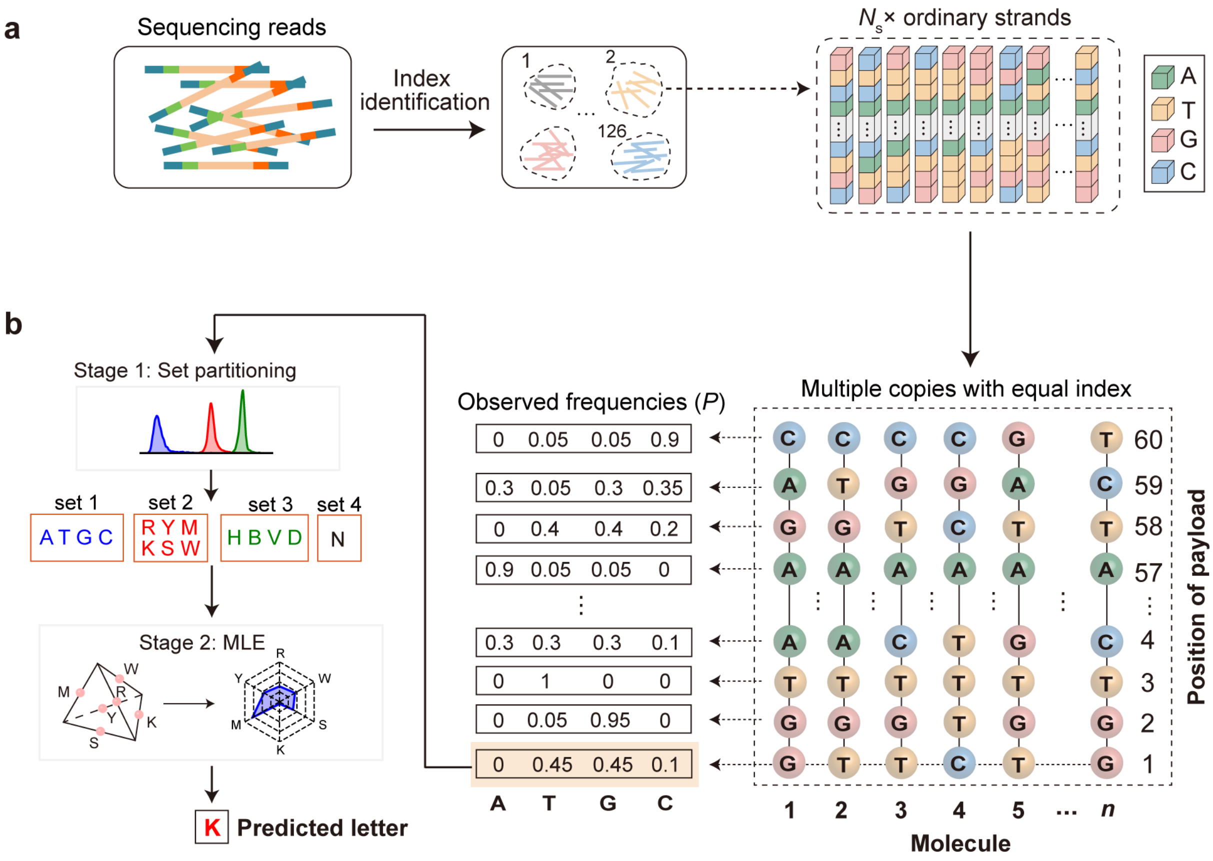

Supplementary Figure 8. Workflow of composite letter detection based on NGS paired-end reads. a, Preprocessing of sequencing reads, including paired-end assembly, primer identification, length filtering, and index identification. **b**, Two-stage composite letter detection: (1) Set partitioning based on observed base frequencies and information entropy. (2) Letter inference within each partitioned subset using a maximum likelihood estimation approach.

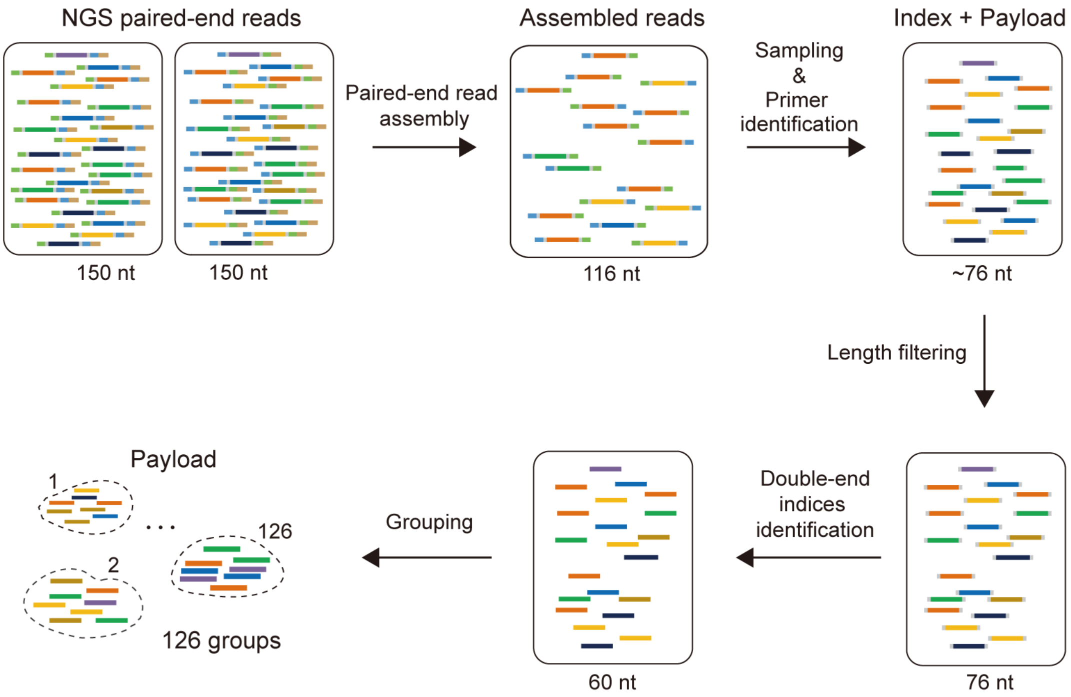

Supplementary Figure 9. Preprocessing of paired-end sequencing reads. Preprocessing of raw sequencing reads involves multiple steps, including the assembly of paired-end reads, subsampling at different coverages, primer identification, length filtering, and the identification of double-end indices. After preprocessing, a subset with a specific subsample coverage is obtained, consisting of 126 groups, each containing a set of related read copies. These processed reads are then used for composite strand inference through a two-stage letter detection method.

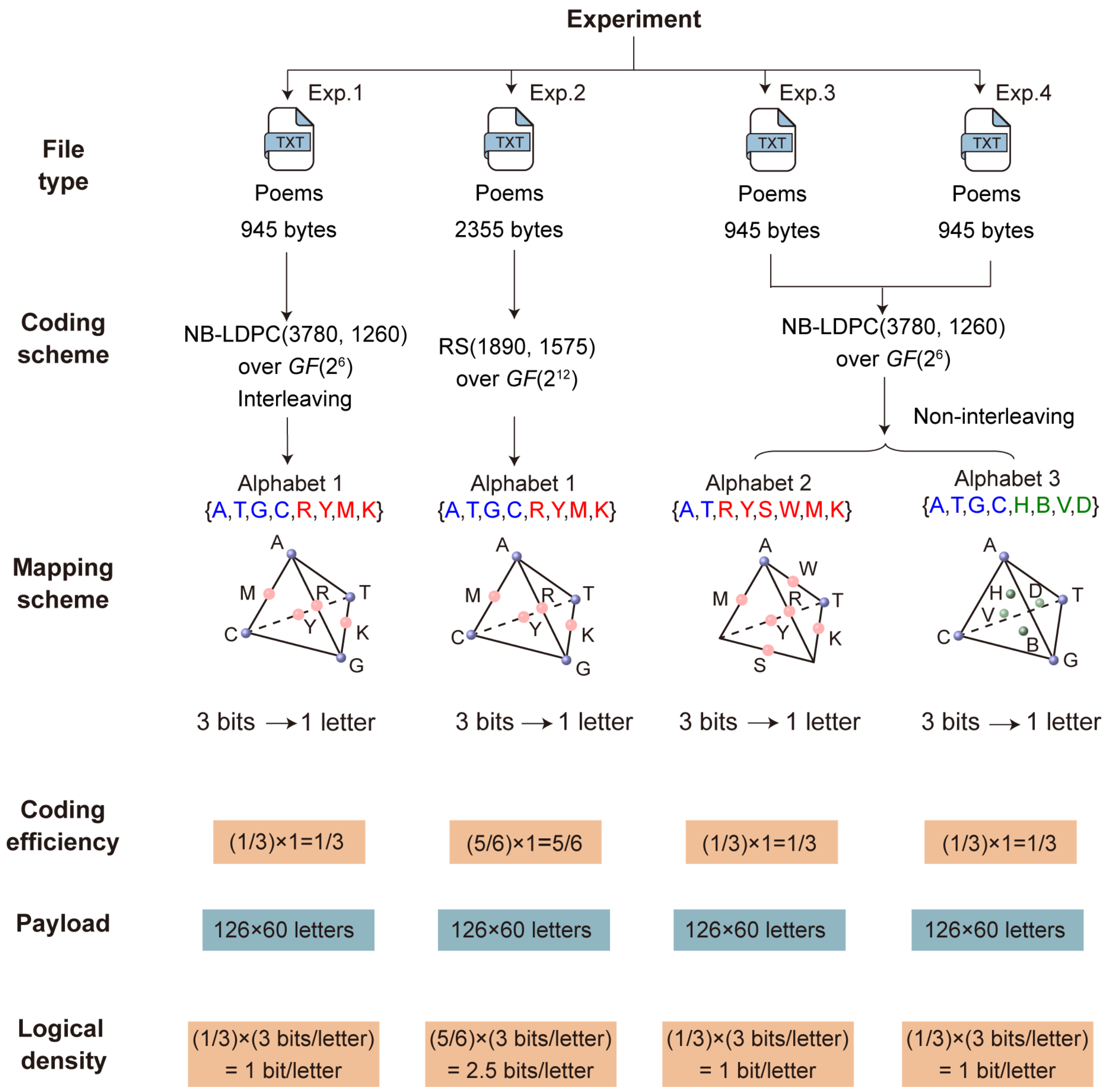

Supplementary Figure 10. Encoding schemes for eight-letter composite letter DNA storage. Three composite alphabets were designed and validated experimentally using different coding schemes. These include a high-error-correction LDPC code (code rate: 0.33) and a high-efficiency RS code (code rate: 0.83), achieving logical densities of 1 bit and 2.5 bits per letter, respectively. A codeword-level symbol interleaving strategy was additionally employed as a control experiment for the LDPC scheme.

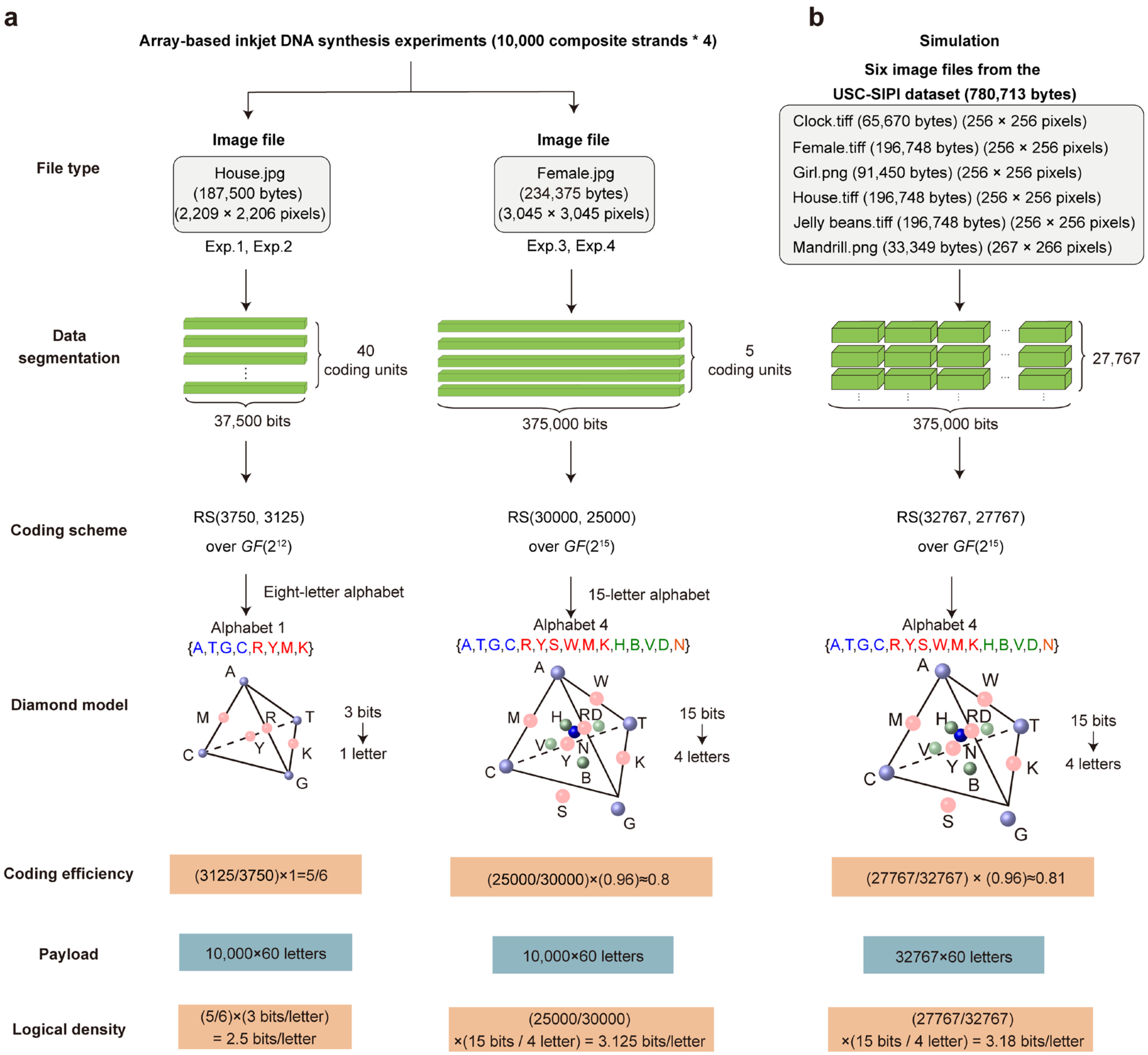

Supplementary Figure 11. Encoding schemes for 15-letter composite letter DNA storage. **a**, Two RS coding schemes were designed for the eight-letter and 15-letter alphabets. Two image files of 187,500 bytes and 234,375 bytes were encoded into pools containing 10,000 strands, respectively. The payload length of each strand was 60 letters. Considering the code rate and bit-to-letter mapping efficiency, the overall encoding efficiencies were 0.83 and 0.80, corresponding to logical densities of 2.5 bits/letter and 3.125 bits/letter of the encoded payload. **b**, Large-scale simulation using RS coding to encode six image files from the USC-SIPI dataset (http://sipi.usc.edu/database/) into a 15-letter composite alphabet, attaining a logical density of 3.18 bits per letter.

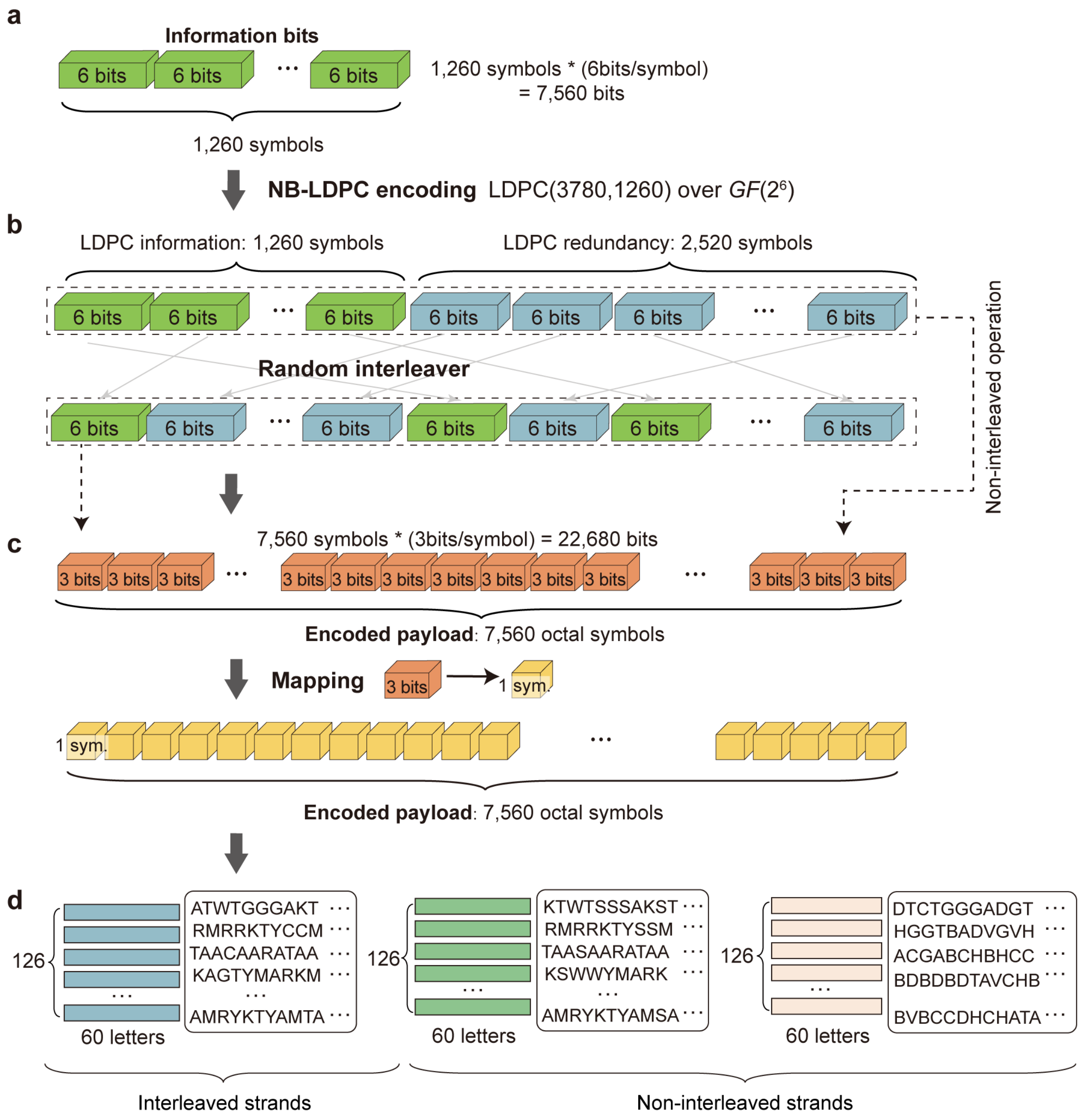

Alphabet 1 {A,T,G,C,R,Y,M,K}

3 bits	sym.	letter	3 bits	sym.	letter
0 0 0	0	A	1 0 0	4	R
0 0 1	1	T	1 0 1	5	Y
0 1 0	2	G	1 1 0	6	M
0 1 1	3	C	1 1 1	7	K

Alphabet 2 {A,T,R,Y,S,W,M,K}

3 bits	sym.	letter	3 bits	sym.	letter
0 0 0	0	A	1 0 0	4	M
0 0 1	1	T	1 0 1	5	K
0 1 0	2	R	1 1 0	6	S
0 1 1	3	Y	1 1 1	7	W

Alphabet 3 {A,T,G,C,H,B,V,D}

3 bits	sym.	letter	3 bits	sym.	letter
0 0 0	0	A	1 0 0	4	H
0 0 1	1	T	1 0 1	5	B
0 1 0	2	G	1 1 0	6	V
0 1 1	3	C	1 1 1	7	D

Supplementary Figure 12. Encoding of eight-letter composite DNA storage (126 composite strands, *R*=1/3). a, A 7,560-bit information sequence is split into 1,260 six-bit symbols. **b**, The 1,260 symbols are encoded with a non-binary LDPC (3780, 1260) code over *GF*(2^6), resulting in a 22,680-bit codeword. A randomly interleaved composite pool serves as a control to evaluate resistance to erasures caused by molecule loss. **c**, The 22,680-bit payload is translated to the composite strands by converting every three bits into one of eight composite letters. **d**, Three synthesis pools are constructed for practical proofs with three different eight-letter alphabets.

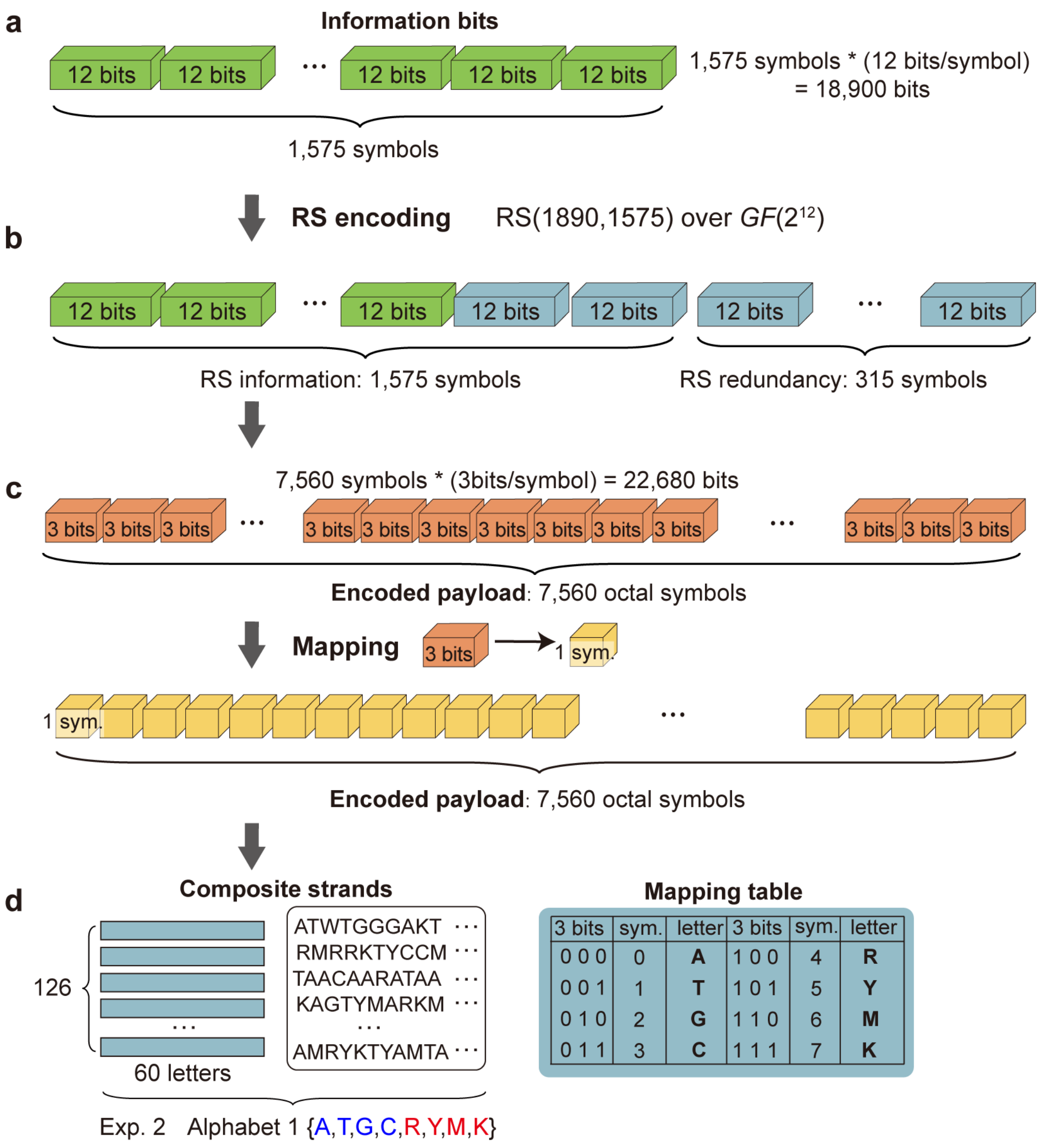

Supplementary Figure 13. Encoding of eight-letter composite DNA storage (126 composite strands, *R*=5/6). a, An 18,900-bit information sequence is split into 1,575 symbols, each containing 12 bits. **b**, The original sequence is encoded to a 2,2680-bit codeword using the RS(1890, 1575) code over $GF(2^{12})$ with a coding rate of 0.83. **c**, The 2,2680-bit payload is mapped into 7,560 composite letters by converting every 3 bits to a single composite letter. **d**, A total of 126 composite strands with an eight-letter composite alphabet are obtained according to the mapping schemes. Based on this high-code-rate coding method, a storage density of 2.5 bits per letter is achieved.

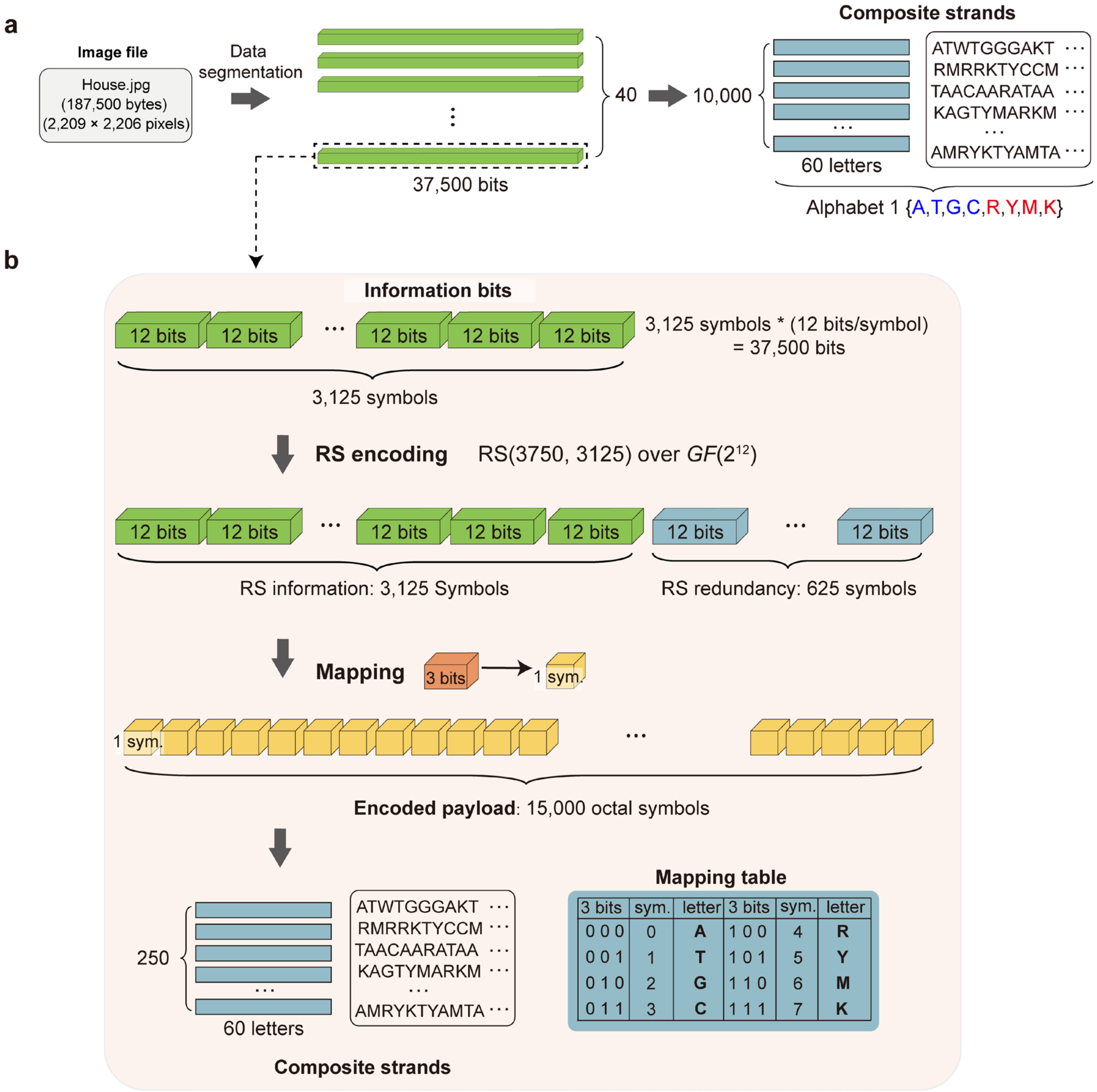

Supplementary Figure 14. Encoding of the eight-letter composite DNA storage system (10,000 composite strands). a, A digital image file of 187,500 bytes was first segmented into 40 data blocks of 37,500 bits and then encoded into 10,000 composite strands. **b**, Each block was divided into 3,125 symbols of 12 bits and encoded using Reed–Solomon (RS) coding, which added 625 redundant symbols for cross-strand error correction (RS(3750, 3125) over $GF(2^{12})$). Each 12-bit RS symbol was then mapped into four octal symbols, which were further converted into composite letters using an eight-letter alphabet {A, T, G, C, R, Y, M, K}. The encoded payload consisted of 15,000 octal symbols, corresponding to 250 composite strands of 60 letters. Each block was independently encoded into 250 composite strands of 60 letters, and a total of 40 data blocks were encoded to obtain 10,000 composite strands. The selected image (House.jpg) is from USC-SIPI dataset (http://sipi.usc.edu/database/).

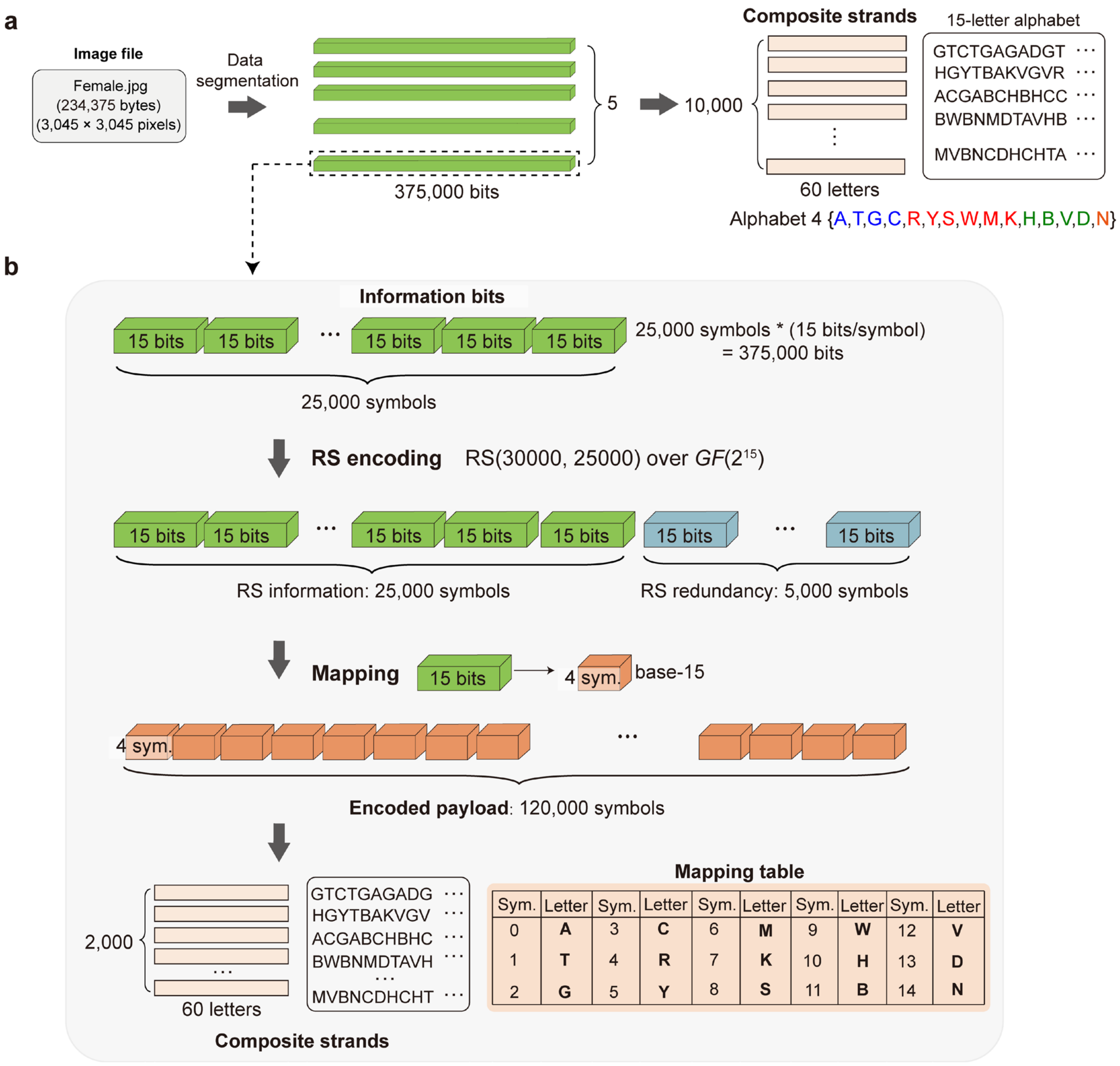

Supplementary Figure 15. Encoding of the 15-letter composite DNA storage system (10,000 composite strands). a, A digital image file of 234,375 bytes was first segmented into 5 data blocks of 375,000 bits and then encoded into 10,000 composite strands. **b**, Each block was divided into 25,000 information symbols of 15 bits and encoded using RS(30000, 25000) coding over $GF(2^{15})$, which added 5,000 redundant symbols for error correction. Each 15-bit symbol was then mapped into four base-15 symbols, which were further converted into composite letters using a 15-letter alphabet {A, T, G, C, R, Y, S, W, M, K, H, B, V, D, N}. The encoded payload consisted of 120,000 symbols, corresponding to 2,000 composite strands of 60 letters. Each block was independently encoded into 2,000 composite strands of 60 letters, and a total of five data blocks were encoded to obtain 10,000 composite strands. The selected image (Female.jpg) is from USC-SIPI dataset (http://sipi.usc.edu/database/).

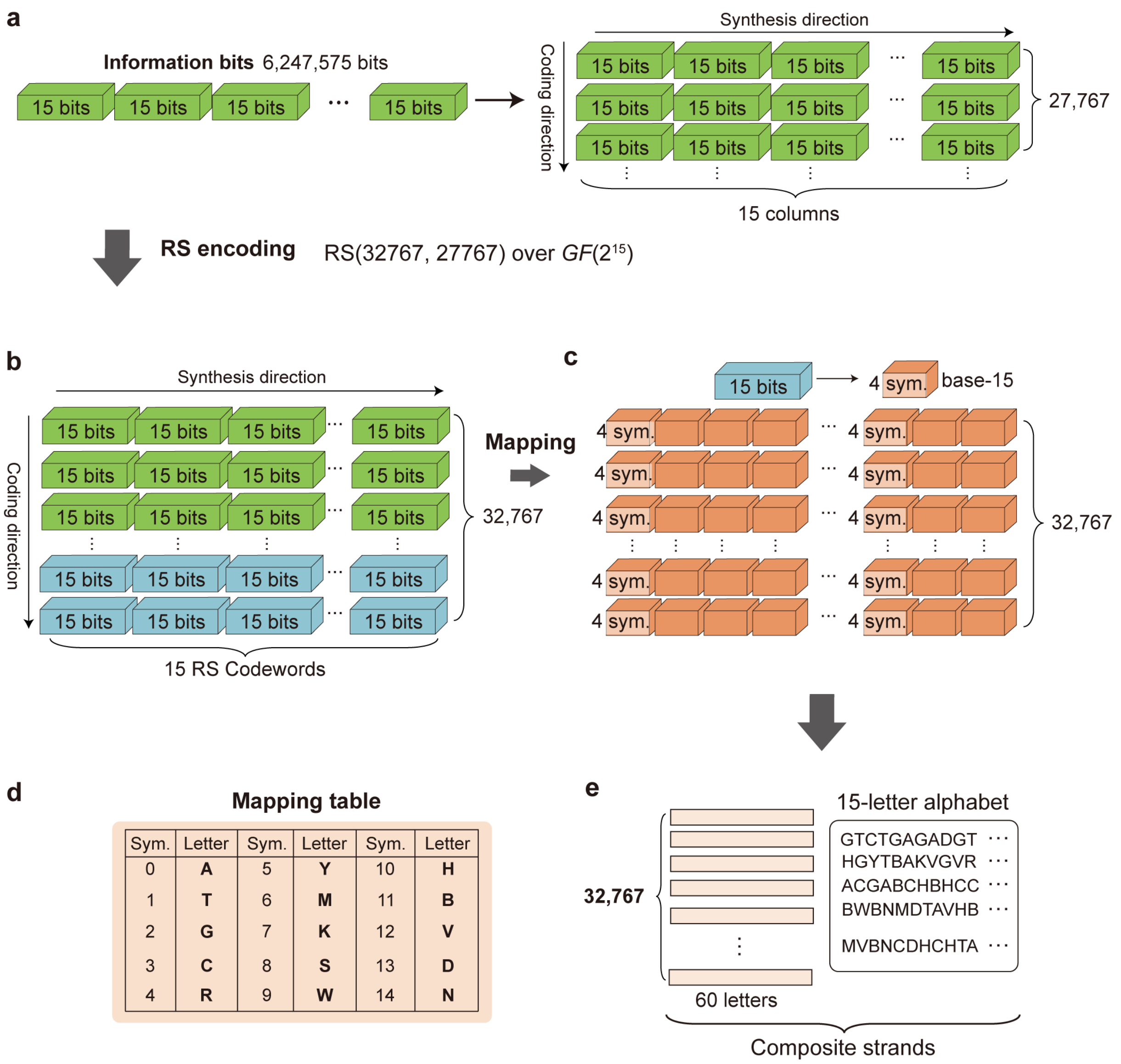

Supplementary Figure 16. Encoding of the 15-letter composite DNA storage system (32,767 composite strands). a, The 6,247,575 information bits are rearranged into an RS encoding matrix consisting of 27,767 rows, each containing 15 chunks, with each chunk comprising 15 bits. **b**, The payload is encoded using an RS(32767, 27767) code over *GF*(2^{15}), generating 15 RS blocks. **c**, Each 15-bit RS symbol is mapped to four base-15 symbols, corresponding to four composite letters. **d**, The mapping relationship between base-15 symbols and the 15-letter composite alphabet. **e**, The RS-encoded symbols are converted into 32,767 composite strands of 60 letters.

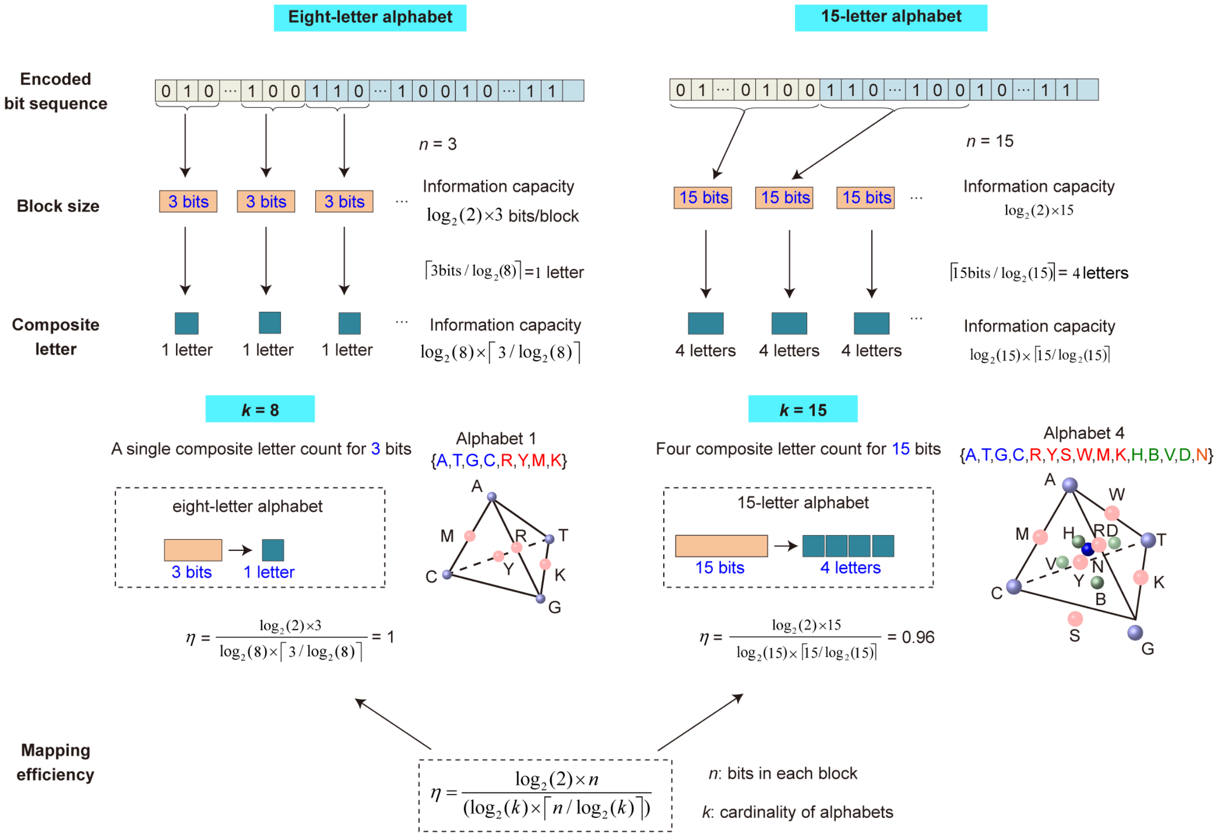

Supplementary Figure 17. Mapping efficiency of bits to composite letters. Binary blocks of n bits are mapped to composite letters from an alphabet of size k, requiring $\lceil n / \log_2(k) \rceil$ letters. Examples show that for an eight-letter alphabet, 3 bits can be perfectly represented by a single composite letter, achieving a mapping efficiency of 100%. In contrast, for a 15-letter alphabet, 15 bits require 4 letters, and the mapping efficiency is 96%.

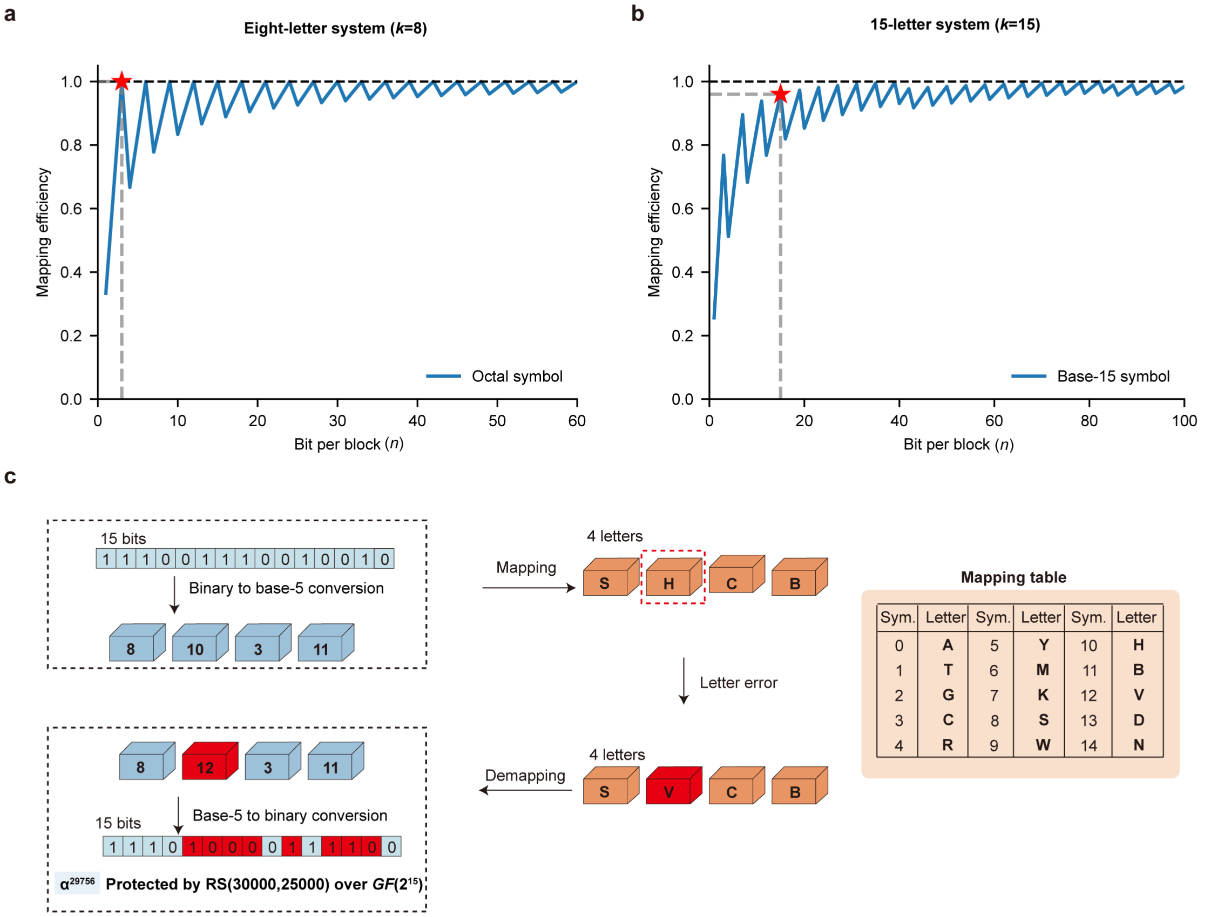

Supplementary Figure 18. Bit-to-alphabet mapping efficiency as a function of bit group size. a, Mapping efficiency from binary bits to the eight-letter alphabets. **b**, Mapping efficiency from binary bits to 15-letter alphabets. Mapping efficiency is defined as the ratio between the number of input bits in each block and the amount of information that can be represented using a fixed number of multi-valued symbols. The red stars indicate the configurations used in our study. Every three bits are mapped to a single octal symbol and a 15-bit group is mapped to four base-15 symbols. For the 15-letter system, this mapping achieves a mapping efficiency of 96%. **c**, Example of letter mapping and de-mapping in the 15-letter composite encoding scheme. During sequencing or synthesis, a letter error may occur (for example, “H” is misread as “V”), leading to an incorrect symbol after de-mapping. Each 15-bit symbol is protected by Reed–Solomon coding (RS(30000, 25000) over $GF(2^{15})$), enabling correction of such symbol-level errors during decoding. Source data are provided as a Source Data file.

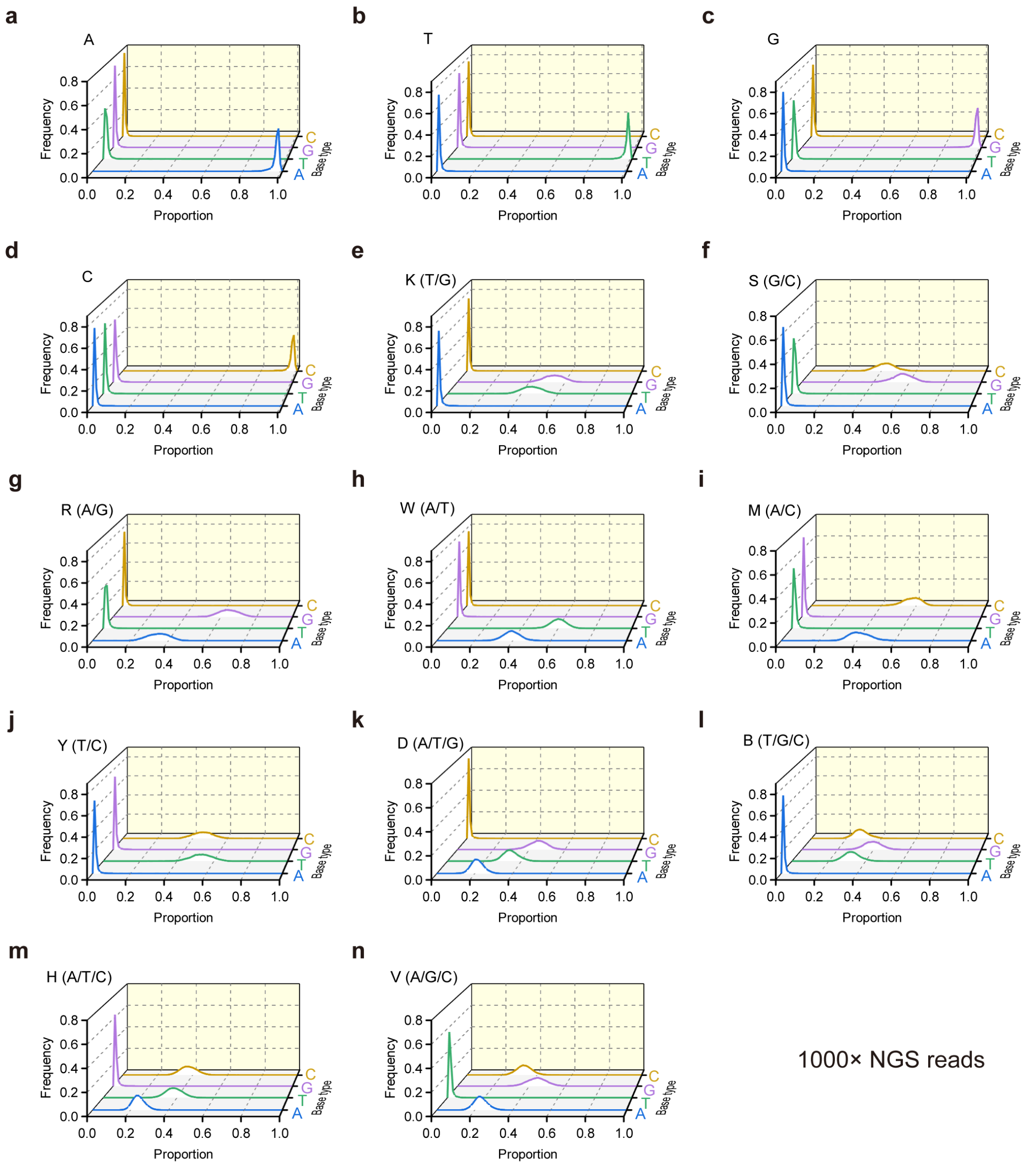

Supplementary Figure 19. Base frequency distribution (1,000×, 126 composite oligos). a–n, Observed frequencies of the four natural bases from 15 composite letters. At a sequencing depth of 1000× coverage, sequencing errors have minimal impact on the peak distribution, and a single peak per base closely approximates the ideal signal model. Source data are provided as a Source Data file.

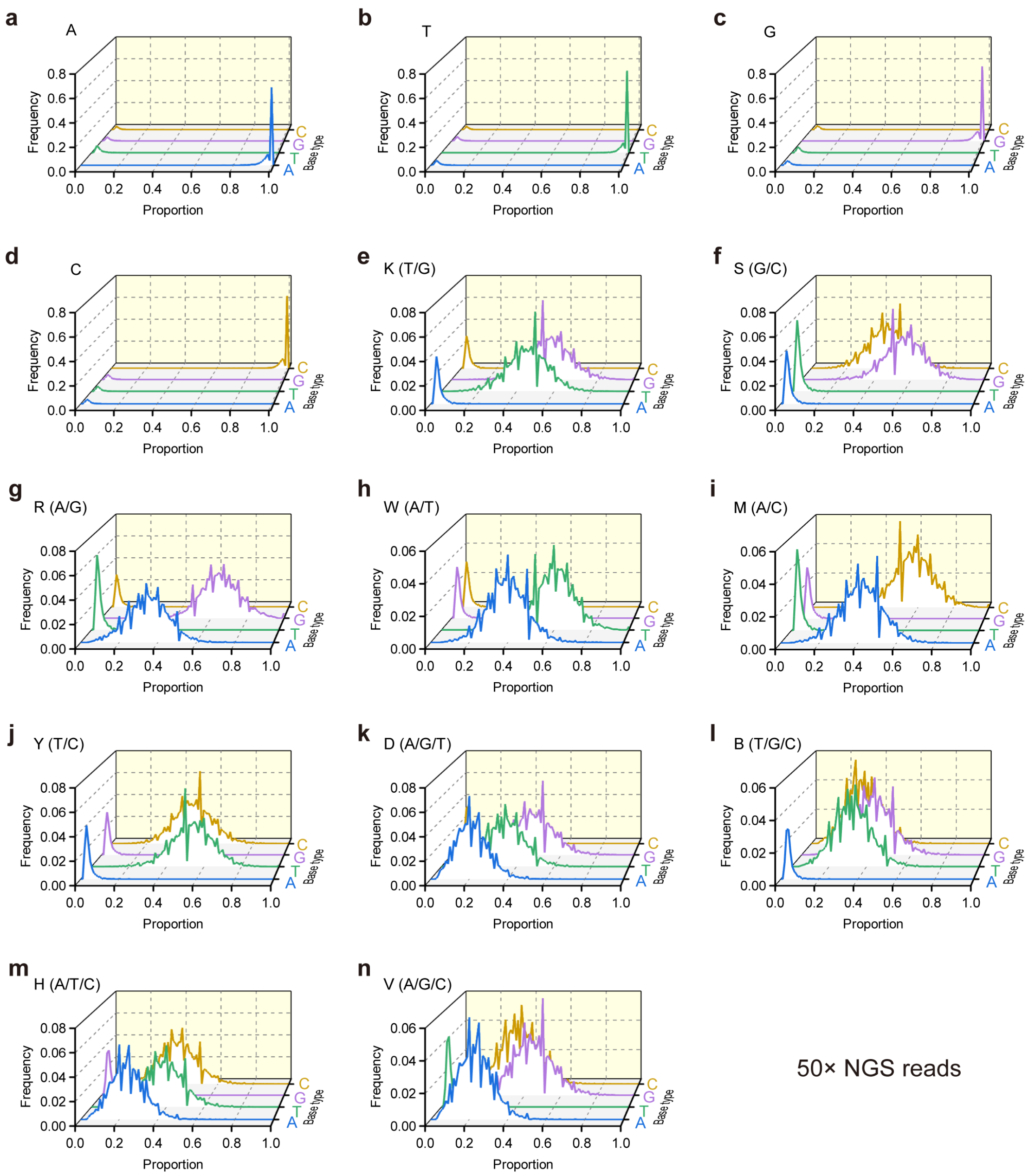

Supplementary Figure 20. Base frequency distribution (50×, 126 composite oligos). a–n, At a sequencing coverage of 50×, the signals of the four bases in composite letters become more fluctuating, transitioning from a single peak at high coverage to multiple peaks. Meanwhile, the frequency distribution range of each base expands, making letter detection more challenging. Source data are provided as a Source Data file.

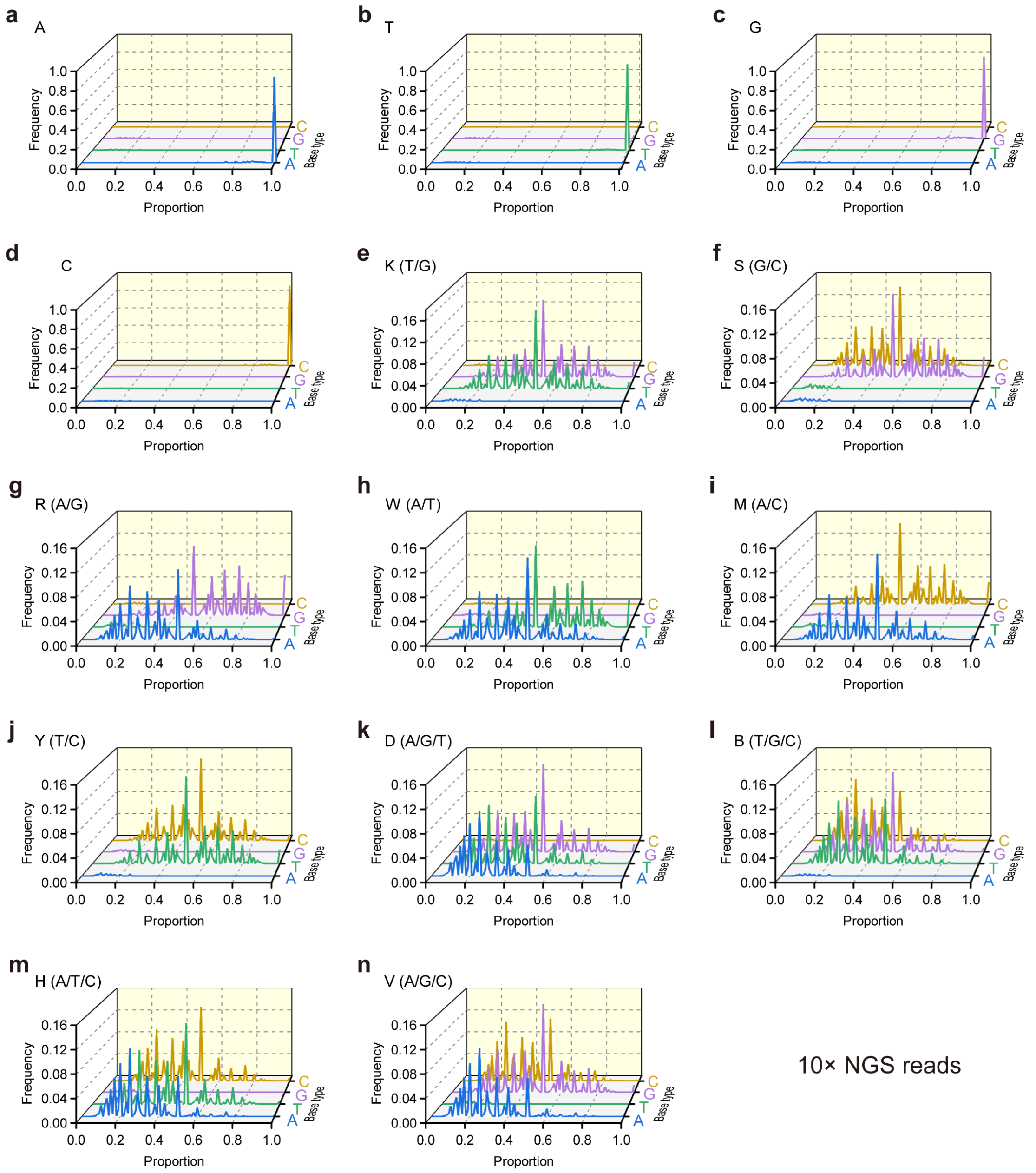

Supplementary Figure 21. Base frequency distribution (10×, 126 composite oligos). a–n, Each letter is represented by the frequency distribution of four natural bases, indicating the sequencing signal of the letter during the sequencing process. At a low sequencing coverage of 10×, increased noise and peak broadening lead to higher uncertainty in composite letter detection. Source data are provided as a Source Data file.

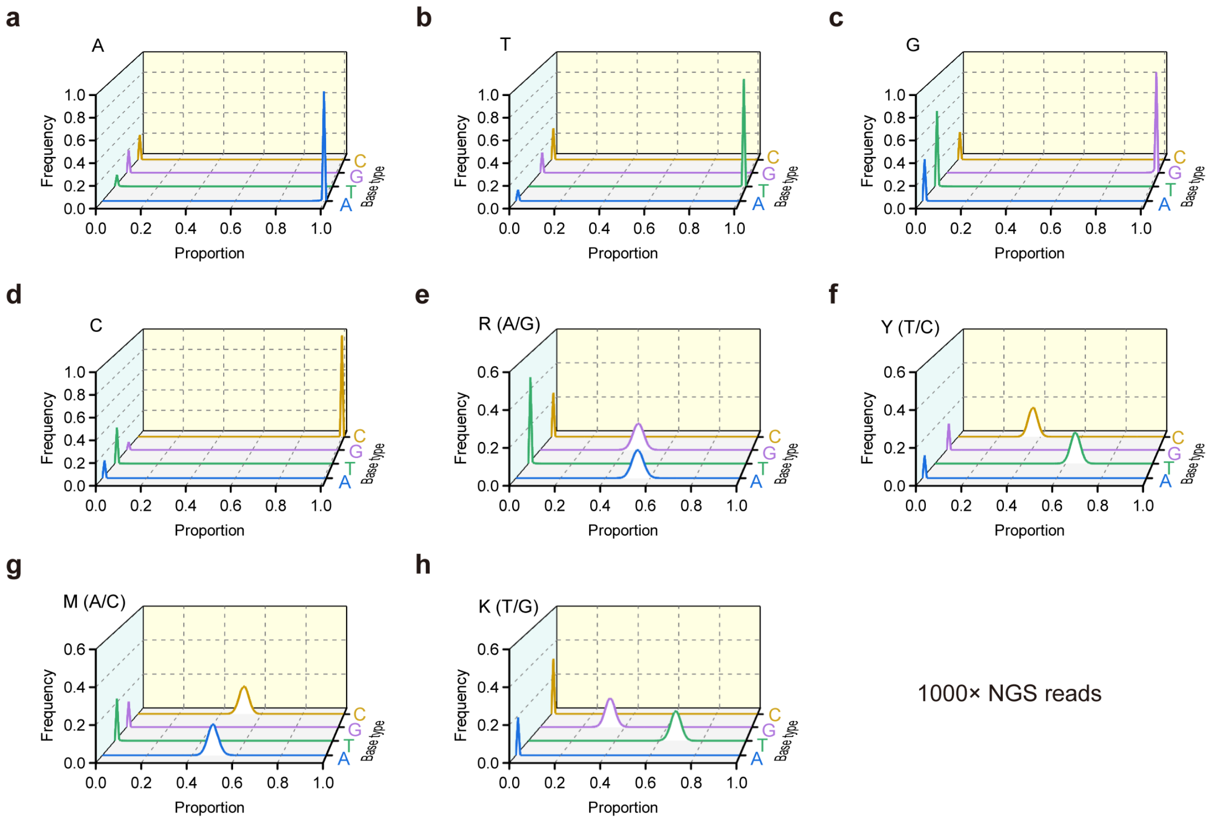

Supplementary Figure 22. Base frequency distribution (1,000×, 10,000 composite oligos, eight-letter alphabet). a–h, Distributions of four constituent bases (A, T, G, C) for each of the eight composite letters at 1,000× average coverage at each synthesis position. The results are derived from sequencing data of 10,000 composite strands encoded with an eight-letter alphabet {A, T, G, C, R, Y, M, K}. Source data are provided as a Source Data file.

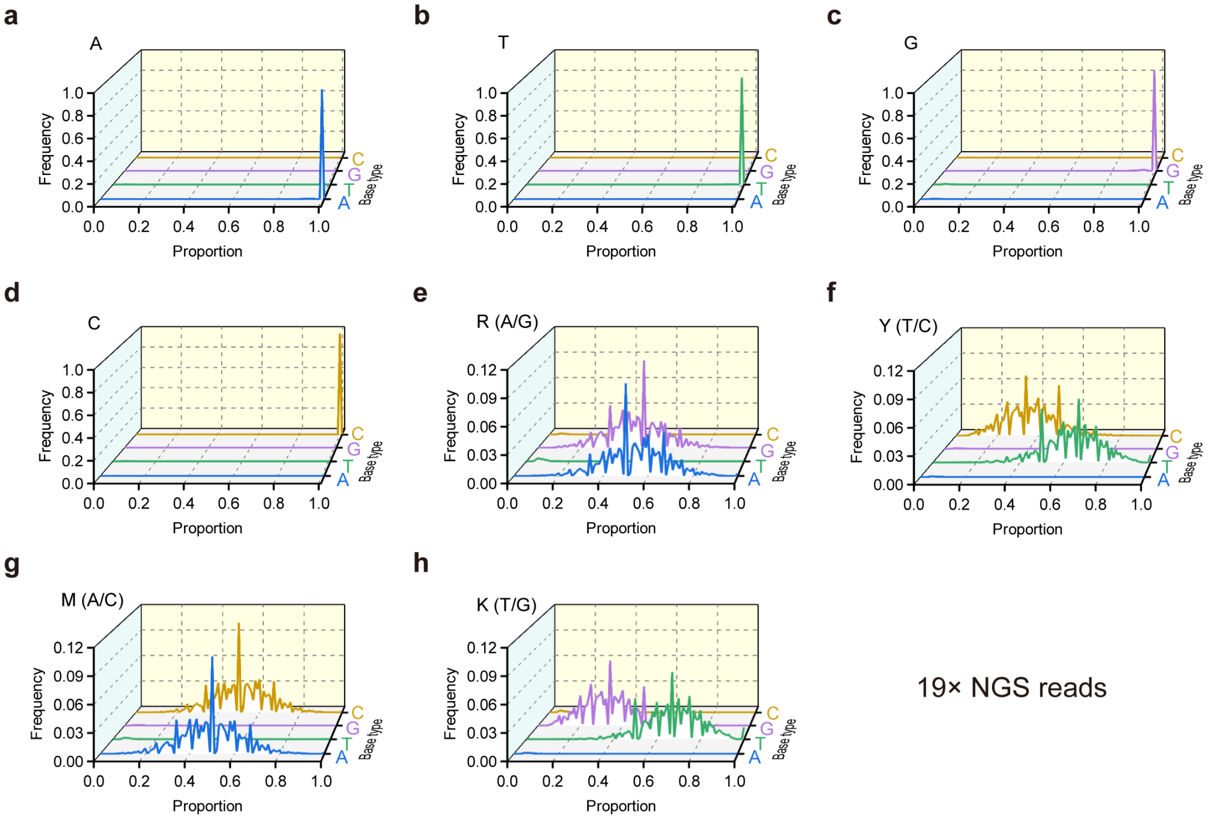

Supplementary Figure 23. Base frequency distribution (19×, 10,000 composite oligos, eight-letter alphabet). a–h, Distributions of four constituent bases (A, T, G, C) for each of the eight composite letters at 19× average coverage, representing the minimum coverage at which error-free data recovery was achieved. The results are derived from sequencing data of 10,000 composite strands encoded with the eight-letter alphabet {A, T, G, C, R, Y, M, K}. Source data are provided as a Source Data file.

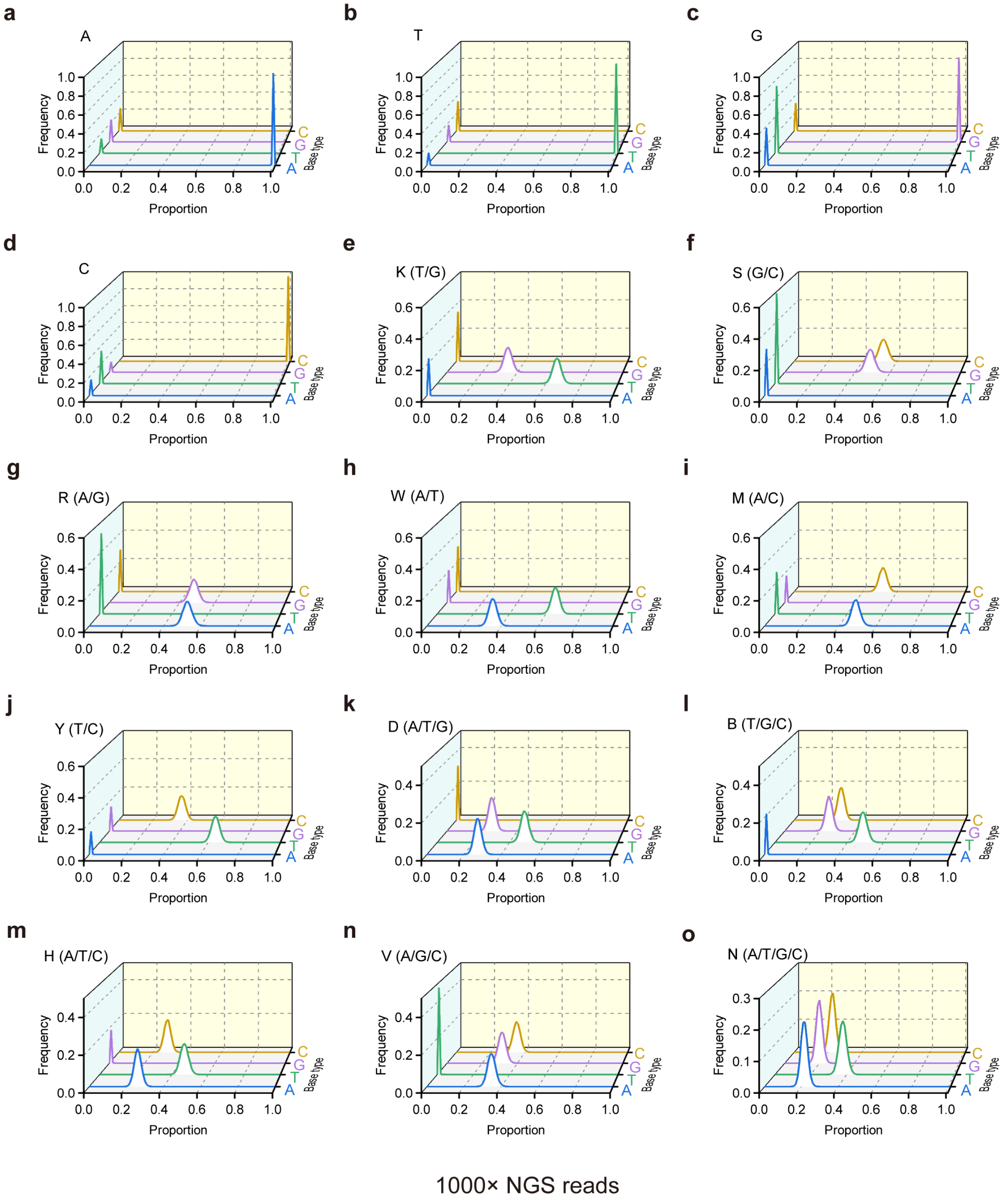

Supplementary Figure 24. Base frequency distribution (1,000×, 10,000 composite oligos, 15-letter alphabet). a–o, Distributions of four constituent bases (A, T, G, C) for each of the 15 composite letters at 1,000× average coverage. The results are derived from sequencing data of 10,000 composite strands encoded with the 15-letter alphabet {A, T, G, C, R, Y, S, W, M, K, H, B, V, D, N}. Source data are provided as a Source Data file.

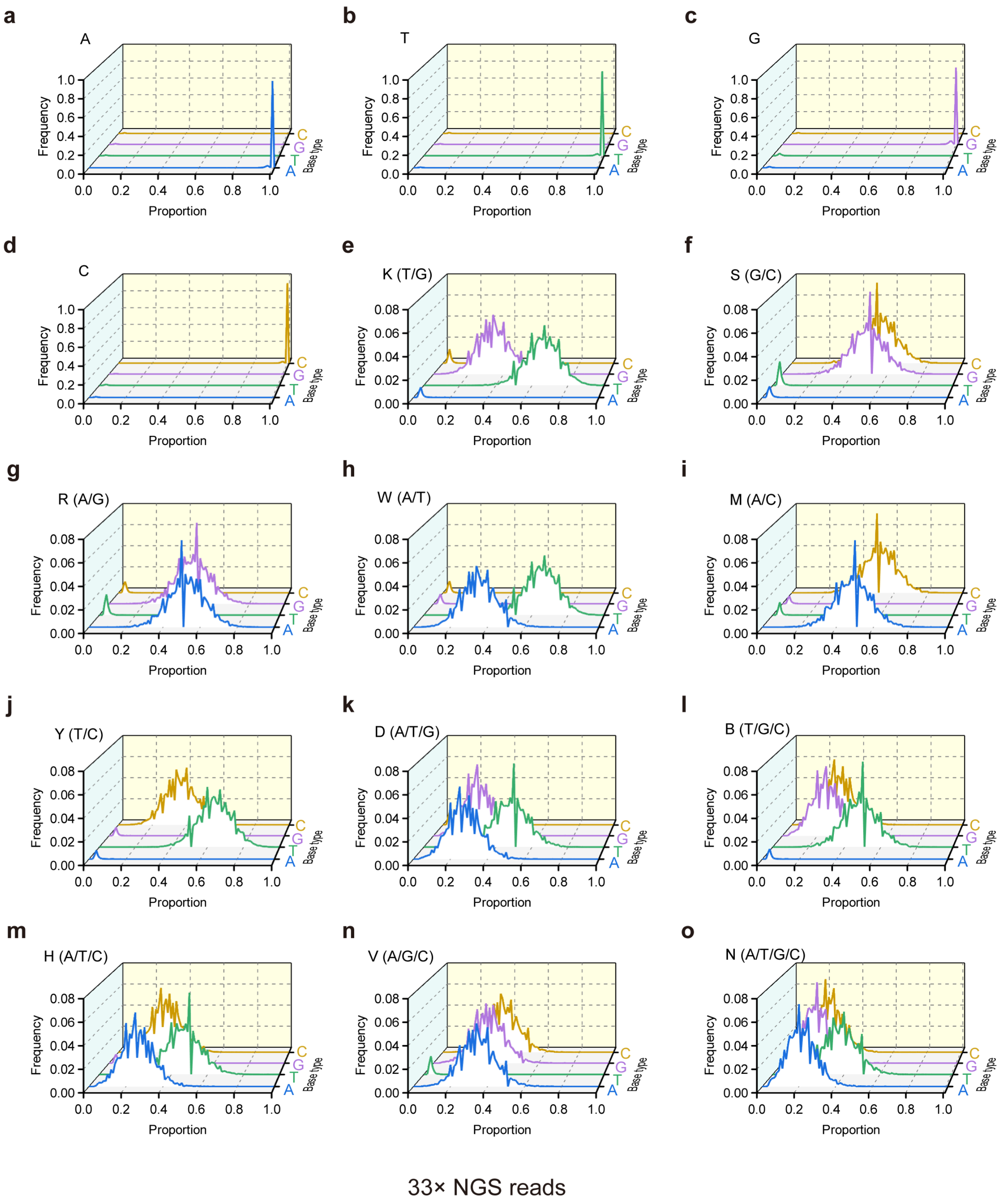

Supplementary Figure 25. Base frequency distribution (33×, 10,000 composite oligos, 15-letter alphabet). a–o, Distributions of four constituent bases (A, T, G, C) for each of the 15 composite letters at 33× average coverage, representing the minimum coverage at which error-free data recovery was achieved. The results are derived from raw sequencing data of 10,000 composite strands with the 15-letter alphabet {A, T, G, C, R, Y, S, W, M, K, H, B, V, D, N}. Source Data are provided as a Source Data file.

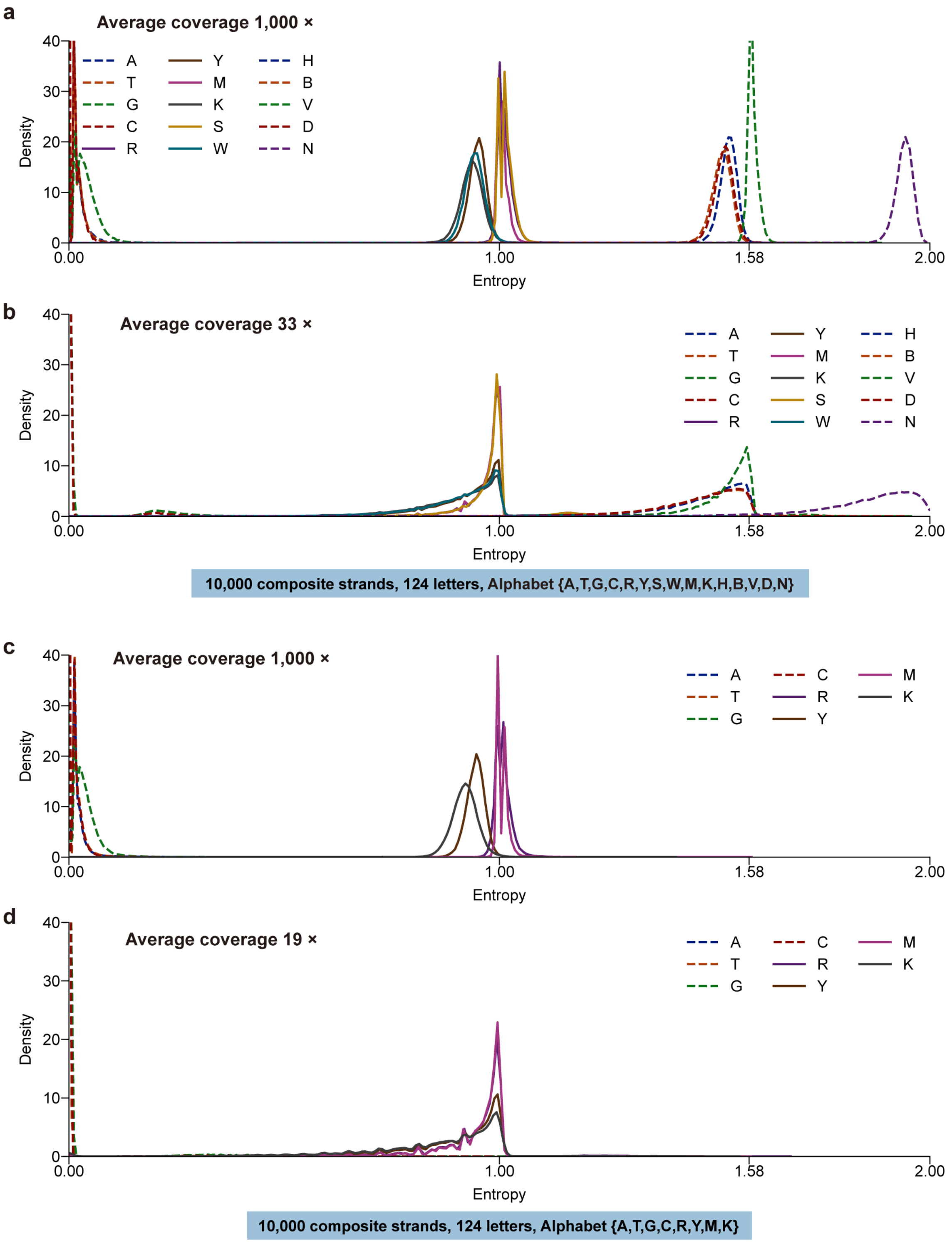

Supplementary Figure 26. Entropy distribution under practical synthesis and sequencing. **a and b**, Entropy distributions of the 15-letter alphabet at high coverage (1,000×) and the minimum coverage for error-free decoding (33×). **c and d**, Entropy distributions of the eight-letter alphabet at high coverage (1,000×) and the minimum coverage for error-free decoding (19×). Each curve represents the entropy distribution for individual composite letters under noisy sequencing conditions. Source data are provided as a Source Data file.

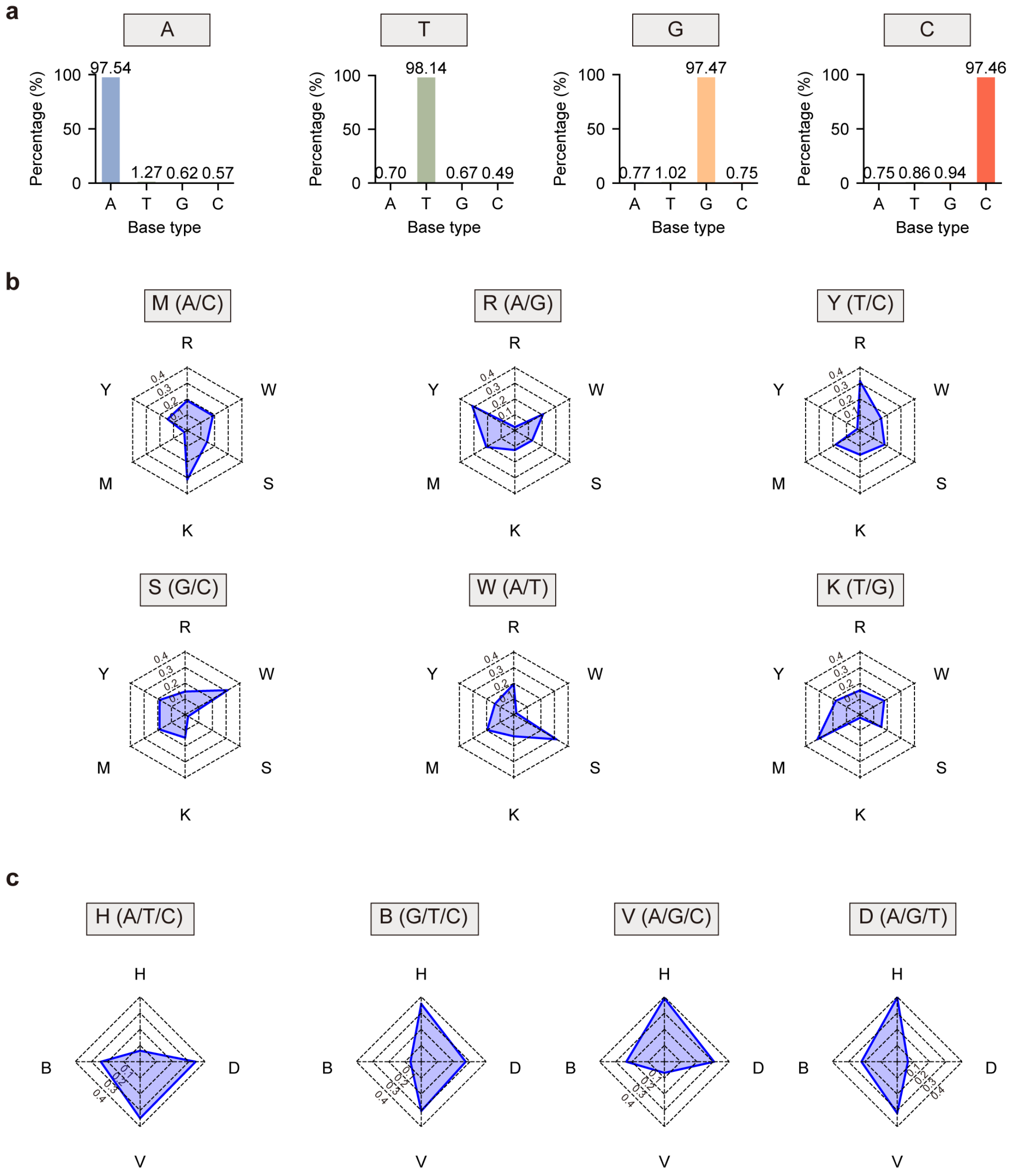

Supplementary Figure 27. Composite letter detection using a normalized maximum likelihood estimation method within subsets. a, For natural bases, the base with the highest frequency is inferred as the correct base. **b** and **c**, Inference results for ten composite letters, with the point closest to the center of the radar plot representing the predicted letter. The results indicate that the normalized maximum likelihood value for correctly inferred letters remains below 0.1. Results are computed from base-frequency observations at 1,000× sequencing coverage (*n* = 126,000 reads). Source data are provided as a Source Data file.

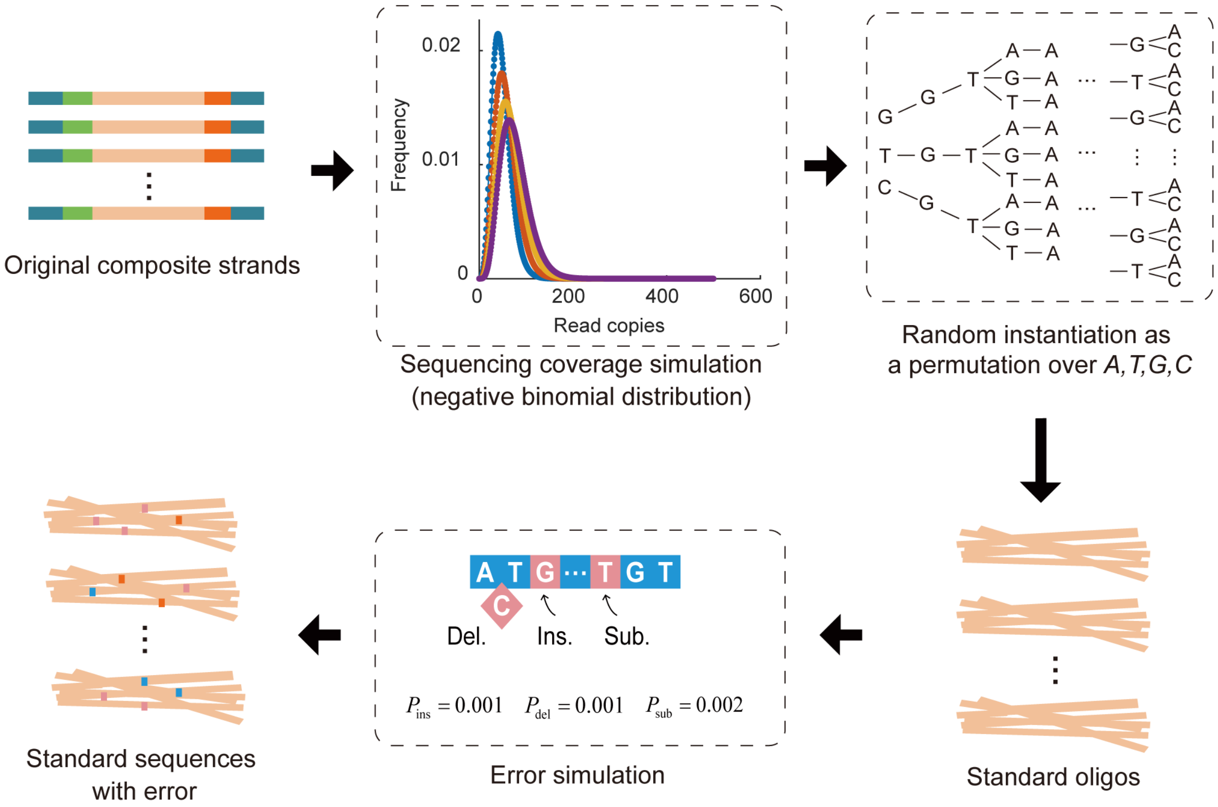

Supplementary Figure 28. Simulation model based on sequencing coverage, error rate, and random instantiation. The simulation model incorporates three functions: (1) Sequencing coverage modeling using a negative binomial distribution. (2) Composite letters random instantiation into four natural bases (A, T, G, C) based on the predefined ratios. (3) Insertion, deletion, and substitution error simulation. The error rates of the three types of errors can be set freely.

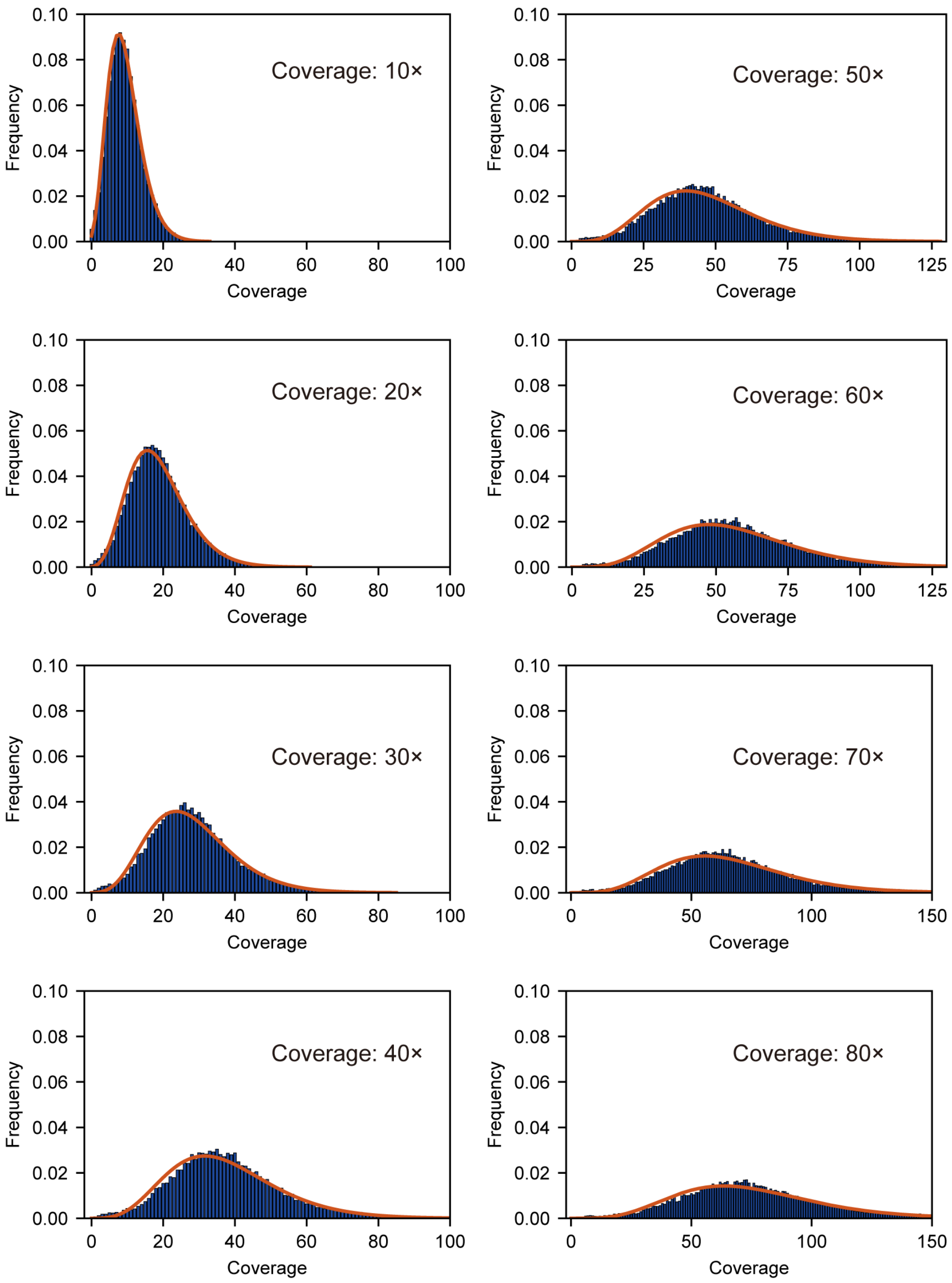

Supplementary Figure 29. The number of sequencing reads associated with each encoded composite strand at different coverages. This result was generated from sequencing reads of a DNA pool containing composite letters formed by an eight-letter alphabet {A, T, G, C, H, B, V, D}. As the average sequencing coverage increases, the distribution range for each unique sequence becomes broader. Due to PCR amplification bias and stochastic sampling, the 126 unique molecules are represented unevenly. The overlaid orange curves represent negative binomial distributions fitted to the read copies. Source data are provided as a Source Data file.

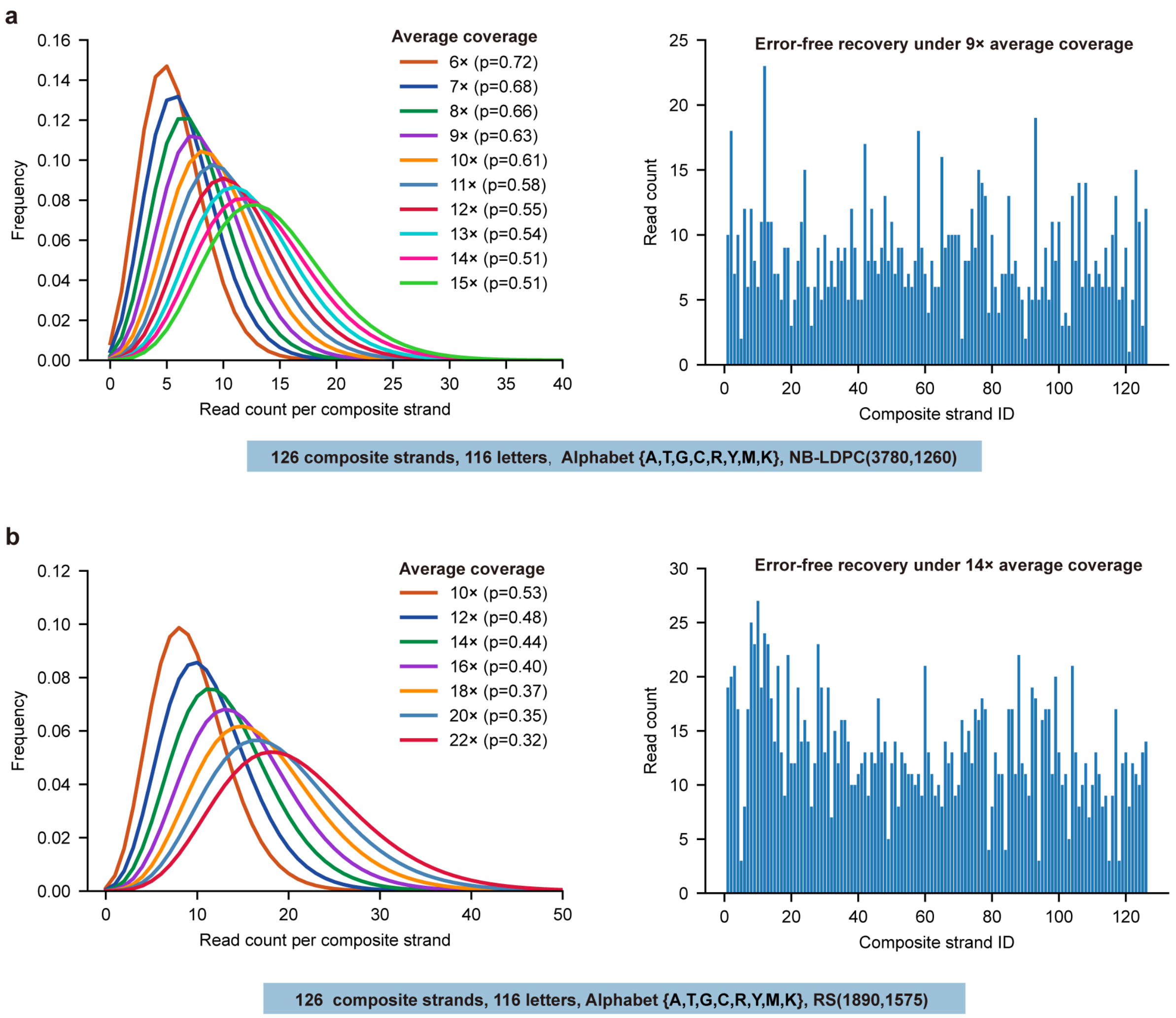

Supplementary Figure 30. Read count distribution of composite DNA strands at different average coverages (126 composite strands). a, Distribution of sequencing read counts per composite strand synthesized by the column-based method (NB-LDPC(3780, 1260) code with an eight-letter alphabet). The left panel shows that the read count distributions at different coverages follow a negative binomial distribution. The parameter *p* denotes the fitted success probability of the negative binomial model. The right panel shows the read count distribution across 126 composite strands at 9× average coverage, where error-free data recovery was achieved. The read counts exhibited noticeable non-uniformity. **b,** Distribution of sequencing read counts from the composite pool encoded with the RS(1890, 1575) code using the eight-letter alphabet. The left panel shows the fitted negative binomial distributions at different coverages. The right panel presents the read-count distribution of individual composite strands (126 in total) at 14× average coverage, where error-free recovery was obtained at this coverage. Source data are provided as a Source Data file.

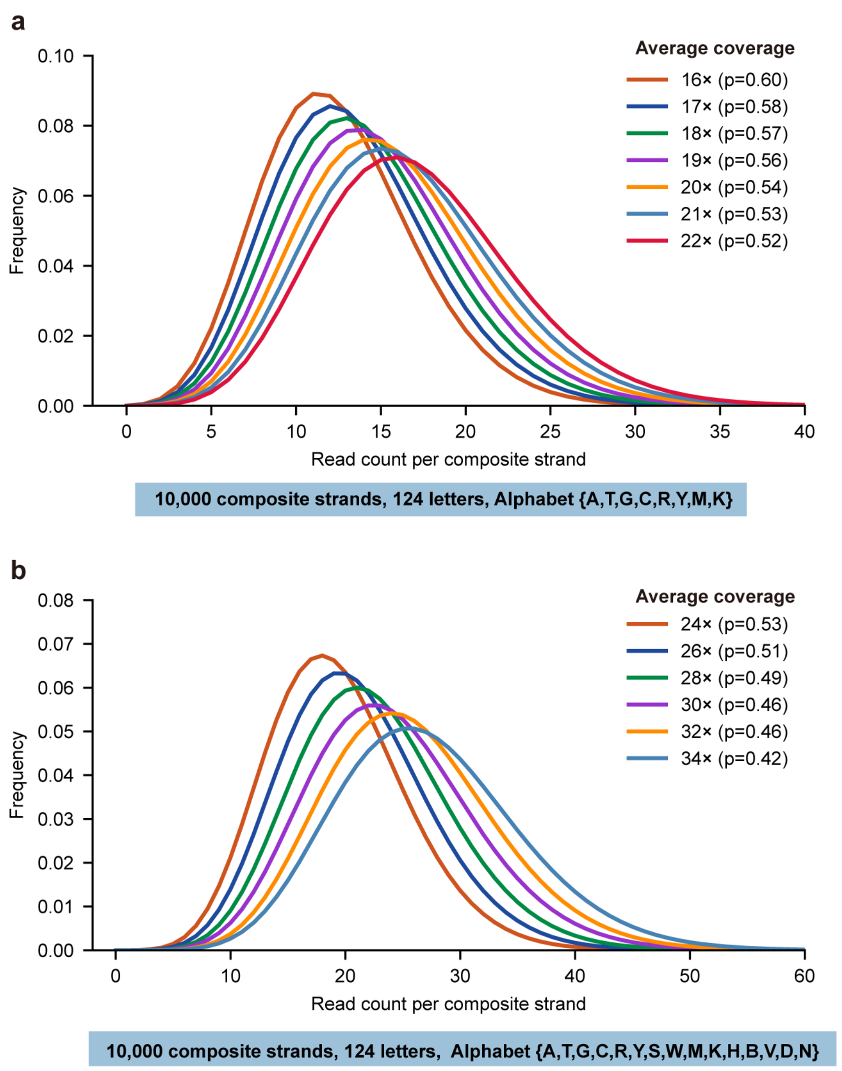

Supplementary Figure 31. Read count distributions at different sequencing coverages (10,000 composite strands). a, Read count distribution of 10,000 composite strands with an eight-letter alphabet (array-based inkjet synthesis). The observed distributions at different average coverages follow negative binomial statistics. **b,** Distribution of read counts for 10,000 composite strands (124 letter length, 15-letter alphabet). In all cases, PCR amplification bias and random sequencing sampling produced negative binomial-like strand copy distributions, and the fitted parameter *p* quantifies the degree of non-uniformity across strands. Source data are provided as a Source Data file.

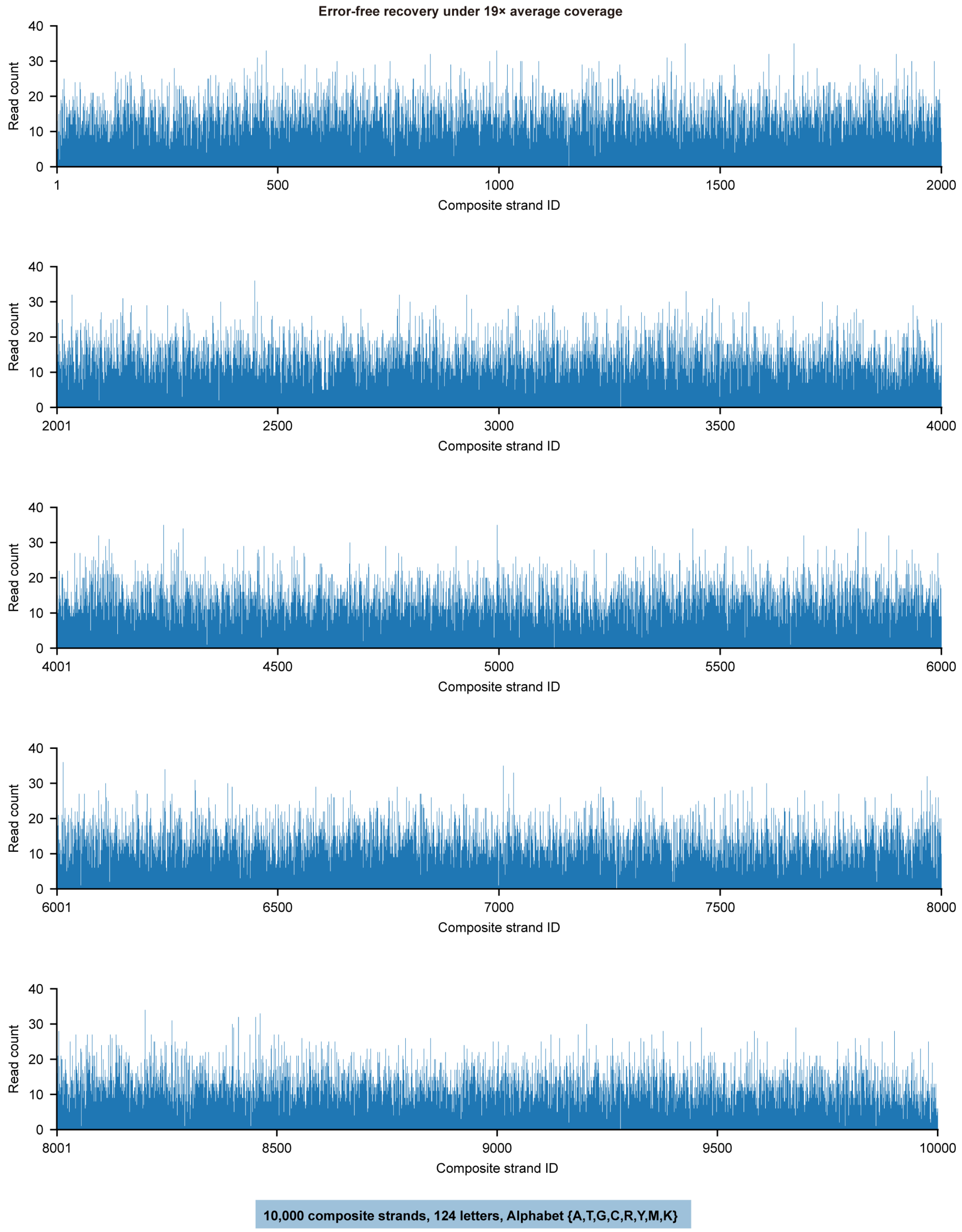

Supplementary Figure 32. Read count distribution of 10,000 individual composite strands with a chosen eight-letter alphabet. Read count distribution of 10,000 composite strands obtained from the pool experiment using the eight-letter composite alphabet at an average sequencing coverage of 19×. Each bar represents the number of sequencing reads corresponding to a single composite strand. Source data are provided as a Source Data file.

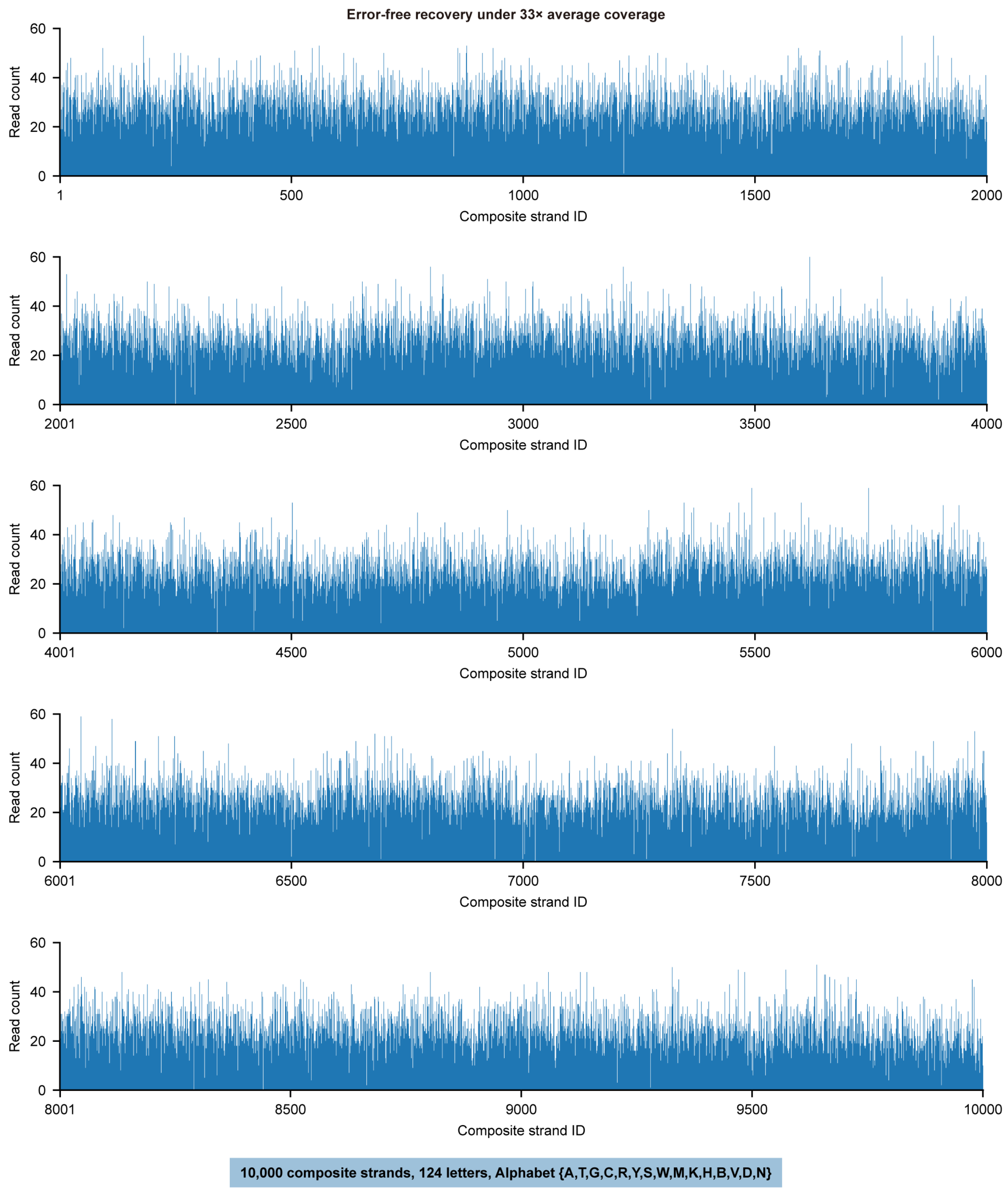

Supplementary Figure 33. Read count distribution of 10,000 individual composite strands with a chosen 15-letter alphabet. The read count distribution of 10,000 composite strands synthesized with the 15-letter composite alphabet at an average sequencing coverage of 33×. The copy number of each strand is non-uniform after sequencing. Source data are provided as a Source Data file.

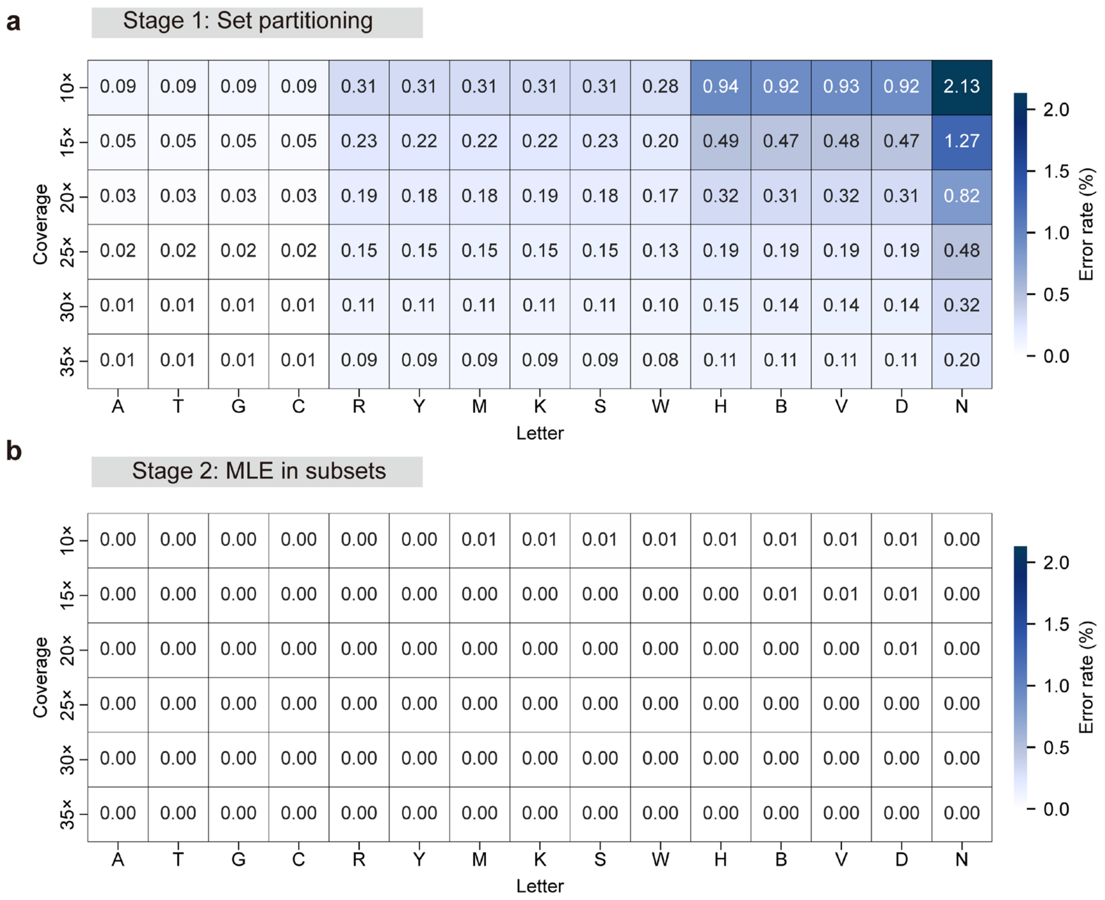

Supplementary Figure 34. Error matrix of the detected composite letters based on a 15-letter alphabet at various sequencing coverages. a, Constituent base frequency variation at low coverage affects set partitioning accuracy. The error rate statistics are based on simulation test data using a 15-letter alphabet. **b**, Letter detection rates using maximum likelihood estimation within each subset. The error frequency matrix indicates the error rate, where the (*i*, *j*) entry represents the frequency of the *i*-th letter being incorrectly inferred at the *j*-th coverage. The set partitioning method improves the detection accuracy within each subset. At the same coverage, letters with higher resolution have higher detection error rates. Source data are provided as a Source Data file.

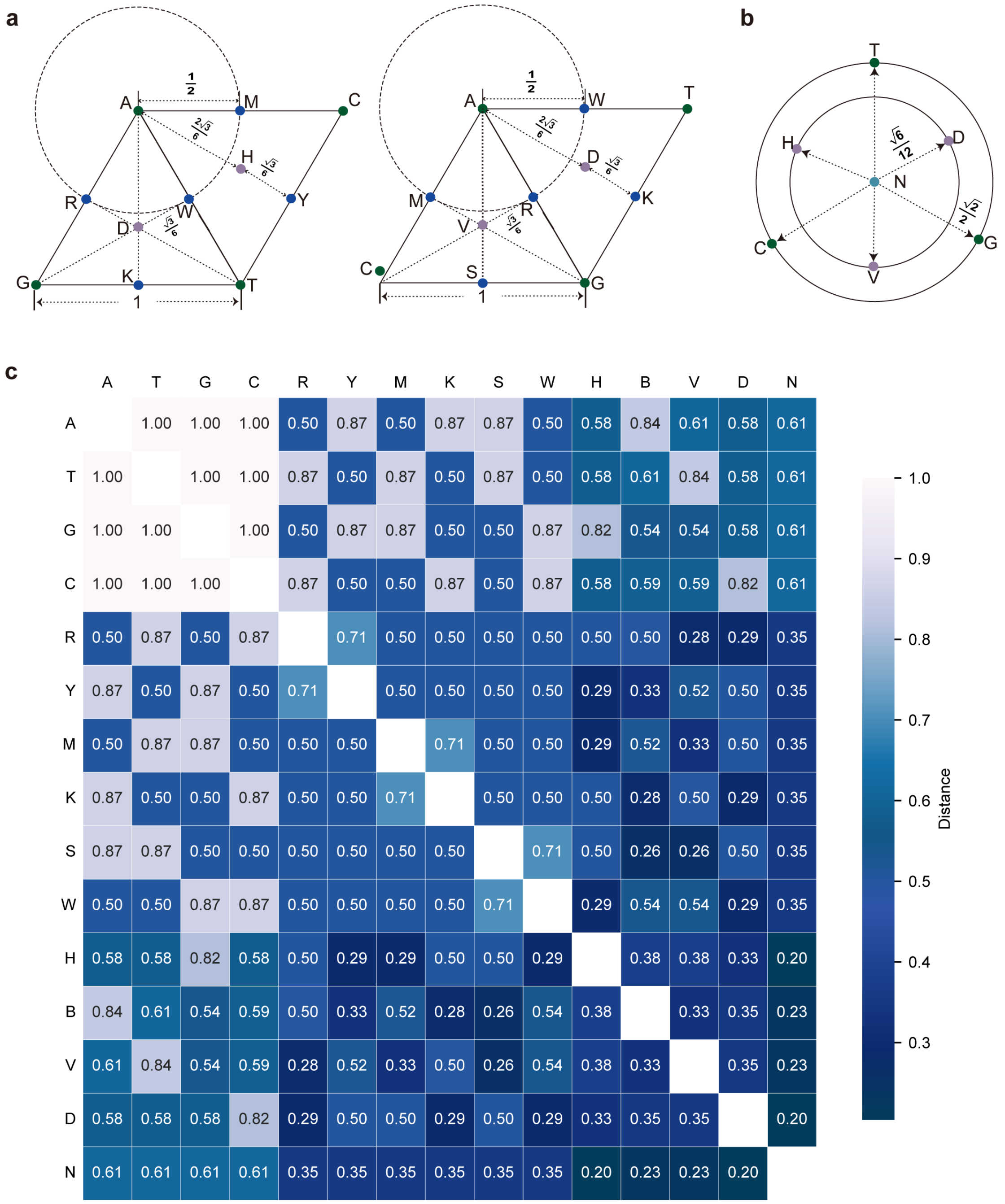

Supplementary Figure 35. Distance distribution between the 15 composite letters in the decomposable diamond model. **a**, Geometric representation of distances between different composite letters in the diamond model. **b**, The distance from the centroid of the tetrahedron to the centers of the faces and edges. **c**, Distance matrix of the 15 composite letters. The distance matrix indicates the distances, where the (i, j) entry represents the distance between the i-th letter and the j-th letter. A smaller value indicates a higher likelihood of error during data retrieval, where a letter is erroneously inferred as a closer one. Source data are provided as a Source Data file.

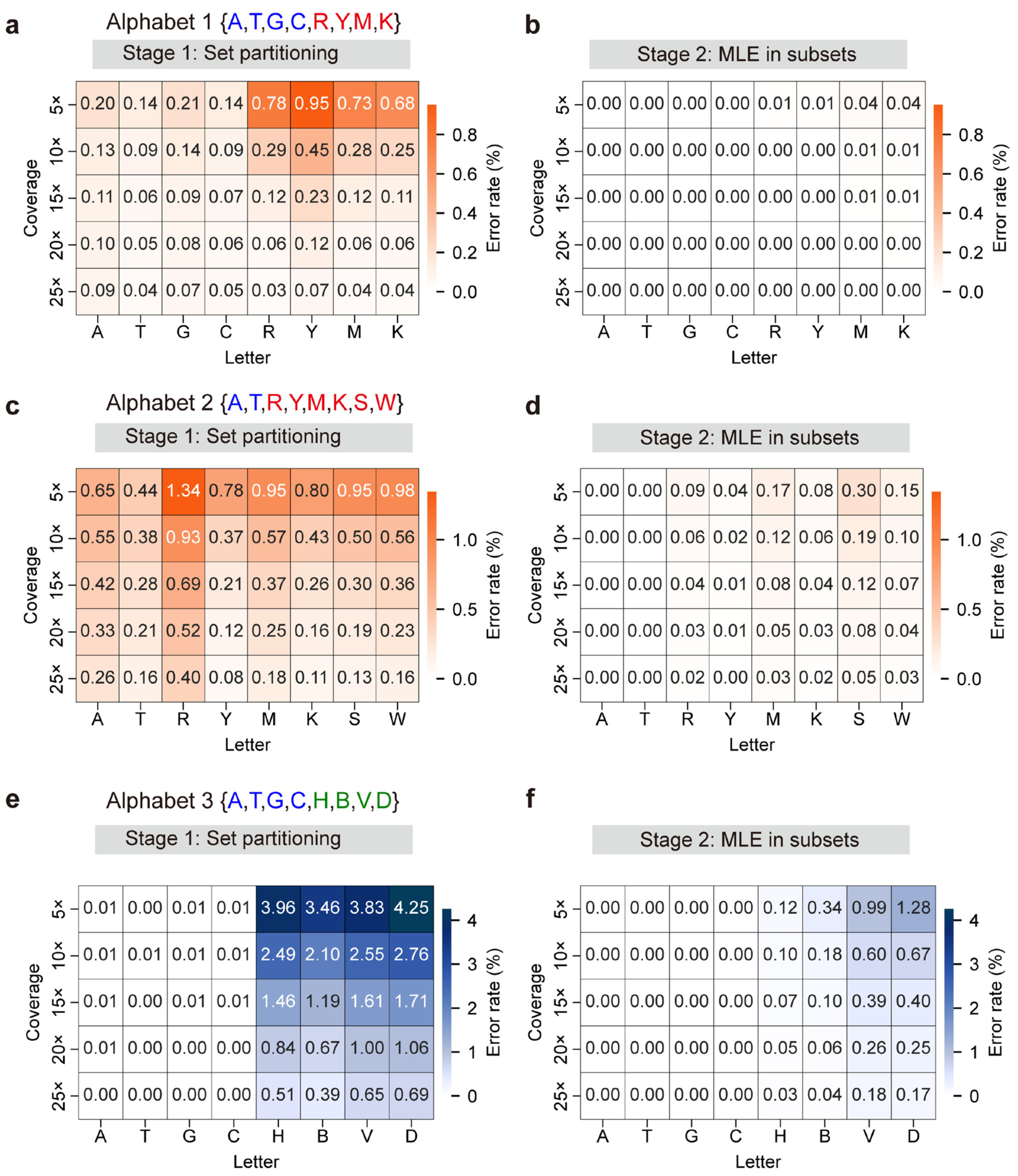

Supplementary Figure 36. Error analysis of the two-stage composite letter detection method based on experimental sequencing data. a and b, Error frequency matrices for an eight-letter alphabet {A, T, G, C, R, Y, M, K} at different sequencing coverages using the two-stage method. **c and d**, Error frequency matrices for an eight-letter alphabet {A, T, R, Y, M, K, S, W} at different coverages. **e and f**, Error frequency matrices of letter detection using an eight-letter alphabet {A, T, G, C, H, B, V, D} at different coverages. The results highlight that the set partitioning in the first stage improves detection accuracy within subsets. Source data are provided as a Source Data file.

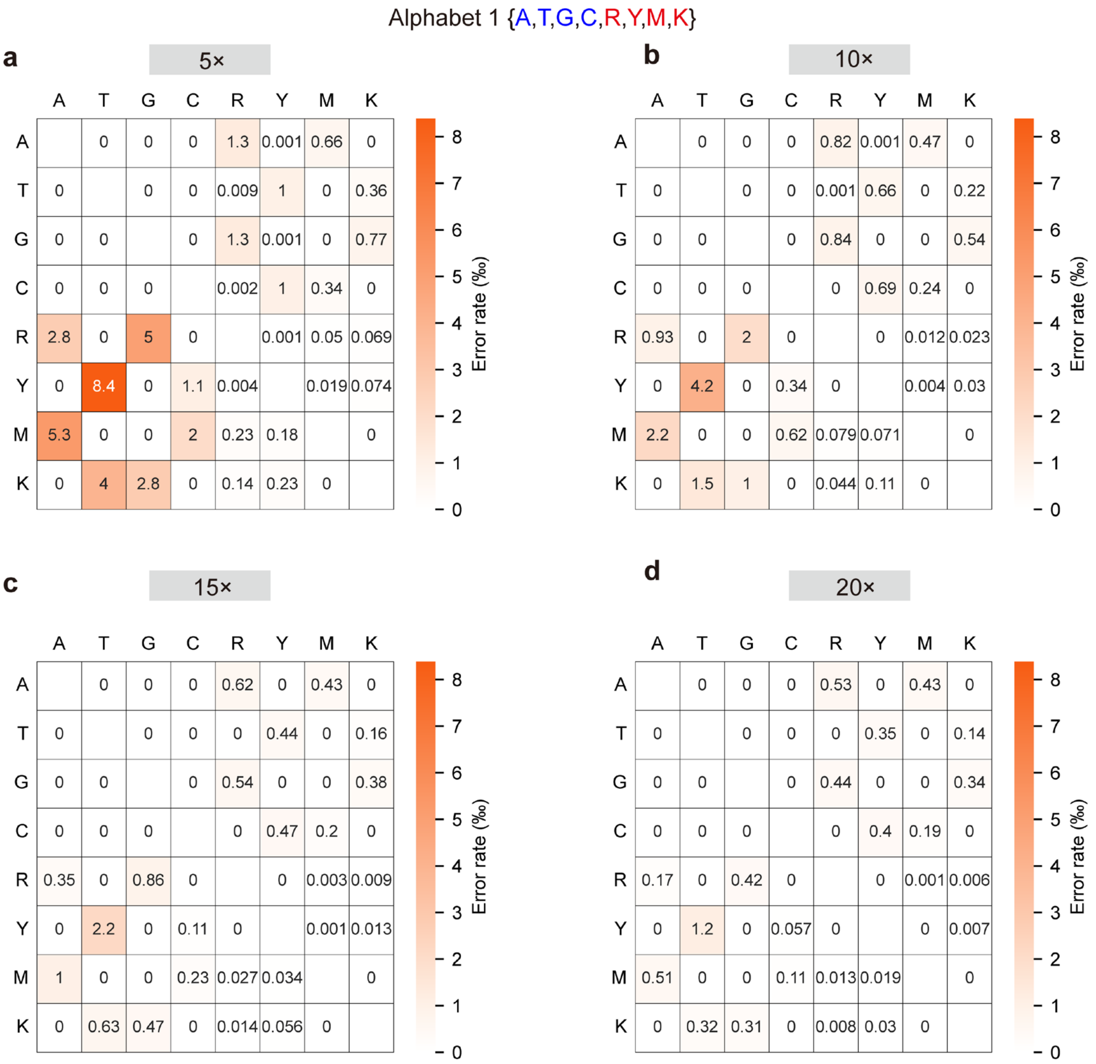

Supplementary Figure 37. Substitution error matrices for the eight-letter Alphabet 1 {A, T, G, C, R, Y, M, K} at four sequencing coverages. Alphabet 1 contains the four natural bases (A, T, G, C) and four composite letters (R, Y, M, K). Each heatmap panel shows the substitution errors that a letter in the row was misidentified as the letter in the column after strand synthesis, sequencing, and letter detection. Rows give the original encoded letters, whereas columns show the letters inferred. **a**, Substitution error matrices at the 5× coverage. Composite letters are frequently misidentified as one of their constituent natural bases (e.g., R → A, 2.8‰; Y → T, 8.4‰; M → A, 5.3‰; K → T, 4‰). **b**, Substitution error matrices at the 10× coverage, where the error rates decreased by at least twofold. (R → A, 0.93‰; Y → T, 4.2‰; M → A, 2.2‰; K → T, 1.5‰). **c**, Substitution error matrices at the 15× coverage. Most off-diagonal error rates drop below 2‰. Only a handful of composite-to-natural substitutions remain appreciable. **d**, Substitution error matrices at a sequencing coverage of 20×. All substitution errors fall below 1.5‰, indicating that increased coverage effectively improves the detection accuracy of composite letters. Source data are provided as a Source Data file.

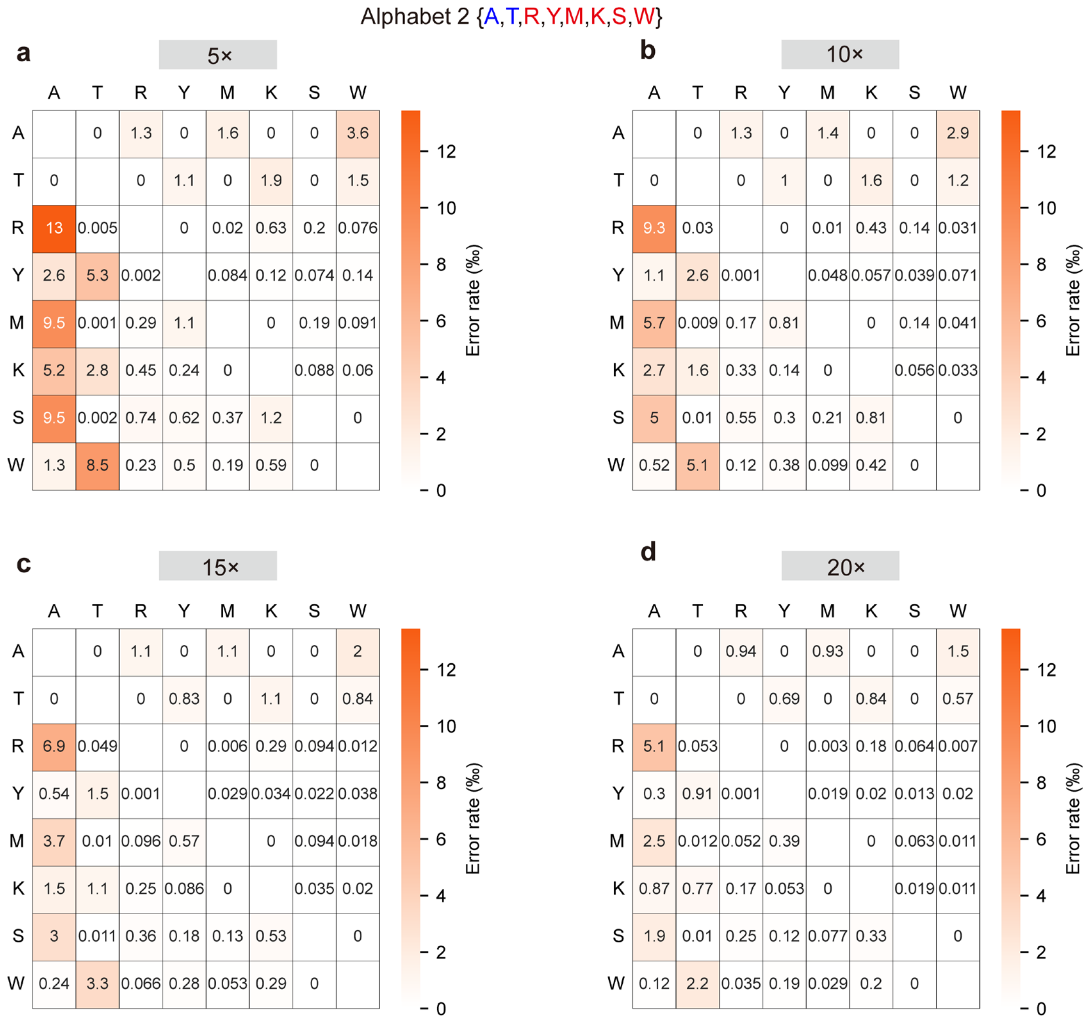

Supplementary Figure 38. Substitution error matrices for the eight-letter Alphabet 2 {A, T, R, Y, M, K, S, W} at four sequencing coverages. Alphabet 2 contains only two natural bases (A, T) but six composite letters (R, Y, M, K, S, W). **a-d**, Substitution error matrices obtained at sequencing coverages of 5×, 10×, 15×, and 20×, respectively. Rows give the original encoded letters and columns give the letters inferred after letter detection; each cell reports the substitution error rates (diagonals omitted). Relative to Alphabet 1{A, T, G, C, R, Y, M, K}, removing two natural bases elevates the share of composite letters, producing markedly higher composite-to-natural substitutions at the 5× coverage. This disparity progressively narrows as coverage rises. Source data are provided as a Source Data file.

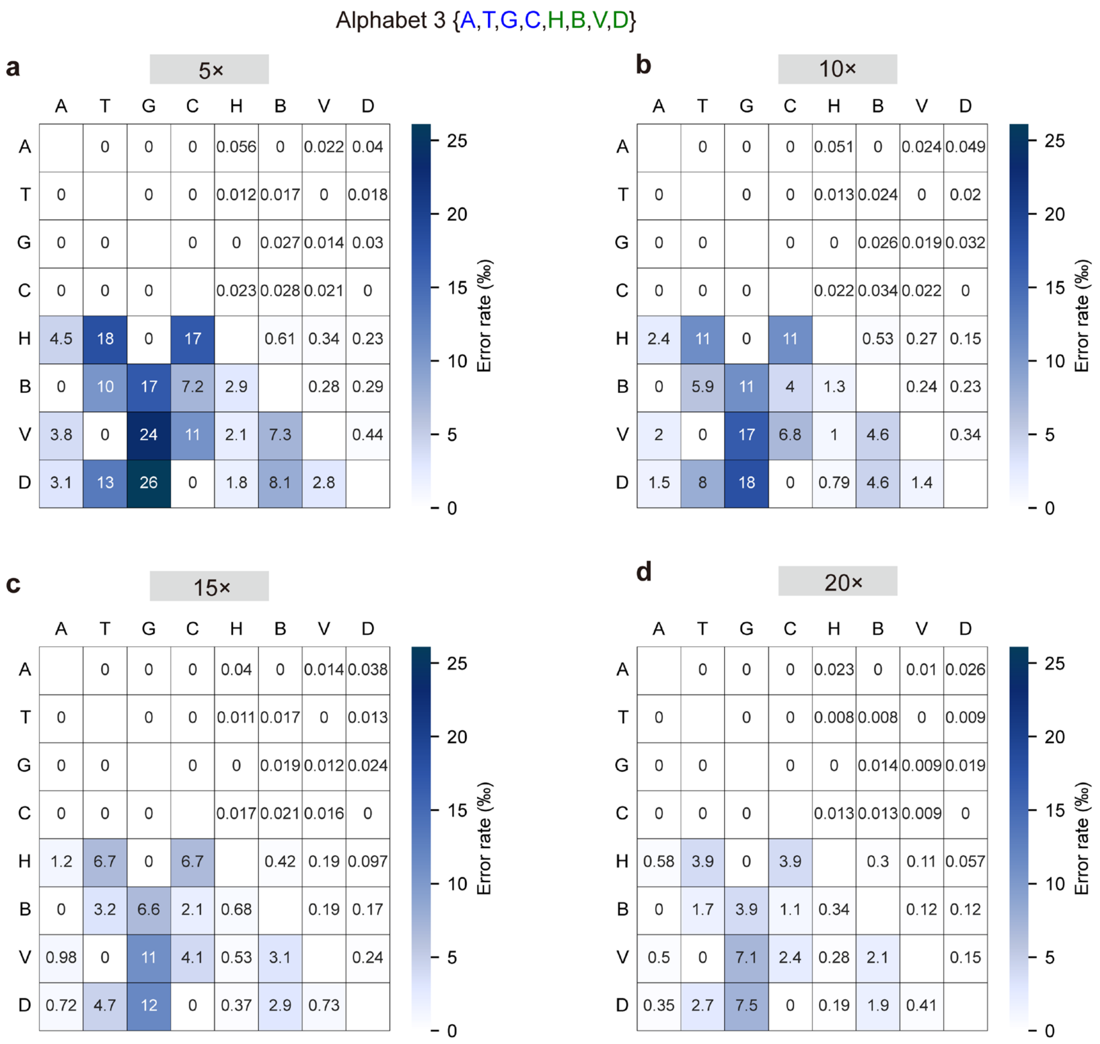

Supplementary Figure 39. Substitution error matrices for the eight-letter Alphabet 3 {A, T, G, C, H, B, V, D} at four sequencing coverages. Alphabet 3 comprises all four natural bases and four ternary composite letters (H = A/C/T, B = C/G/T, V = A/C/G, and D = A/G/T), each representing a controlled mixture of three bases. **a-d**, At 5× coverage, composite letters incur the highest errors, often converting to one of their constituent bases (e.g., V → G 24‰; H → T 18‰; D → G 26‰; B → G 17‰). As sequencing coverage rises, from 5× to 20×, the overall substitution-error rates progressively decline. Together with the matrices for Alphabets 1 & 2, these data show that error frequency scales with mixture complexity. Composite letters (Alphabets 1 & 2) are harder to detect than pure natural bases, while ternary composite letters (Alphabet 3) are harder still, particularly at low sequencing coverage. Source data are provided as a Source Data file.

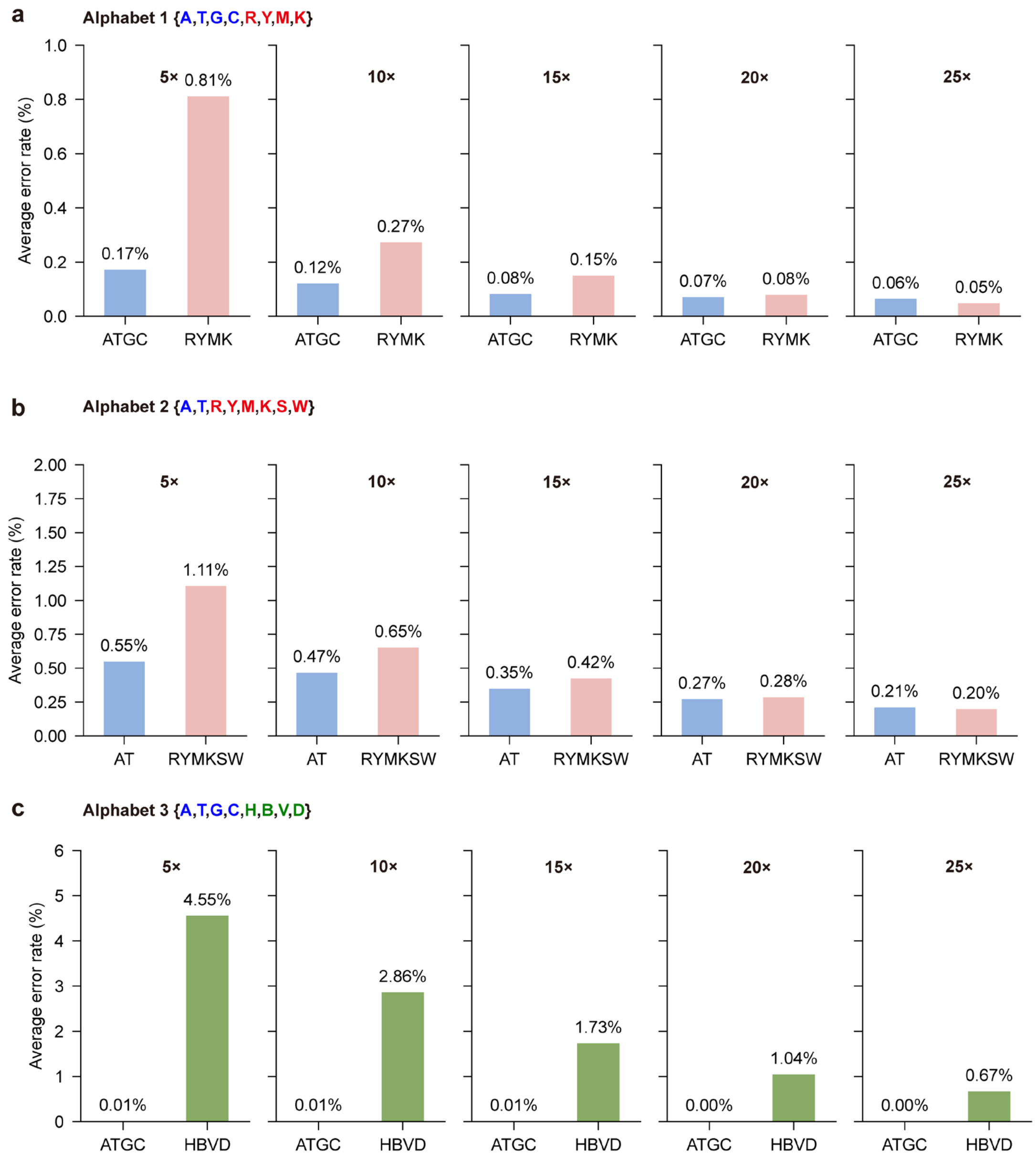

Supplementary Figure 40. Error rates of different composite letters in experimental tests. **a**, Error rates for different composite letters in an eight-letter alphabet {A, T, G, C, R, Y, M, K} based on experimental data. **b**, Error rates for different letters in an eight-letter alphabet {A, T, R, Y, M, K, S, W} based on experimental data. **c**, Error rates for different letters in an eight-letter alphabet {A, T, G, C, H, B, V, D} based on experimental data. The results from three experiments demonstrated that composite letters composed of more complex nucleotide mixtures exhibit higher error rates. As sequencing coverage decreases, their error rates converge toward those of the four natural bases. Among the three eight-letter alphabets, Alphabet 1 delivers the lowest letter error rate. Source data are provided as a Source Data file.

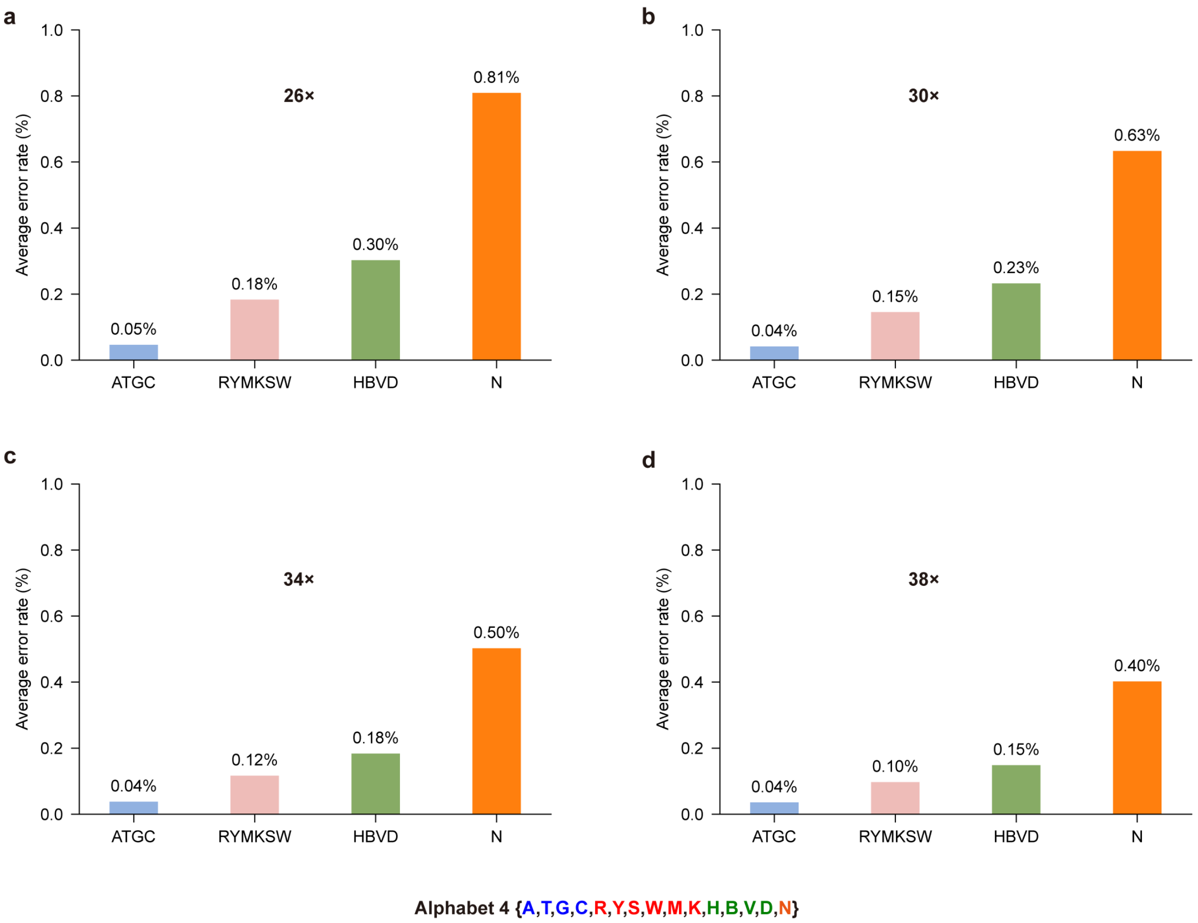

Supplementary Figure 41. Error rates of different letters in a 15-letter composite alphabet. a–d, Error analysis of 15 composite letters {A, T, G, C, R, Y, M, K, S, W, H, B, V, D, N}, showing the variation in error levels across different letter subsets. Source data are provided as a Source Data file.

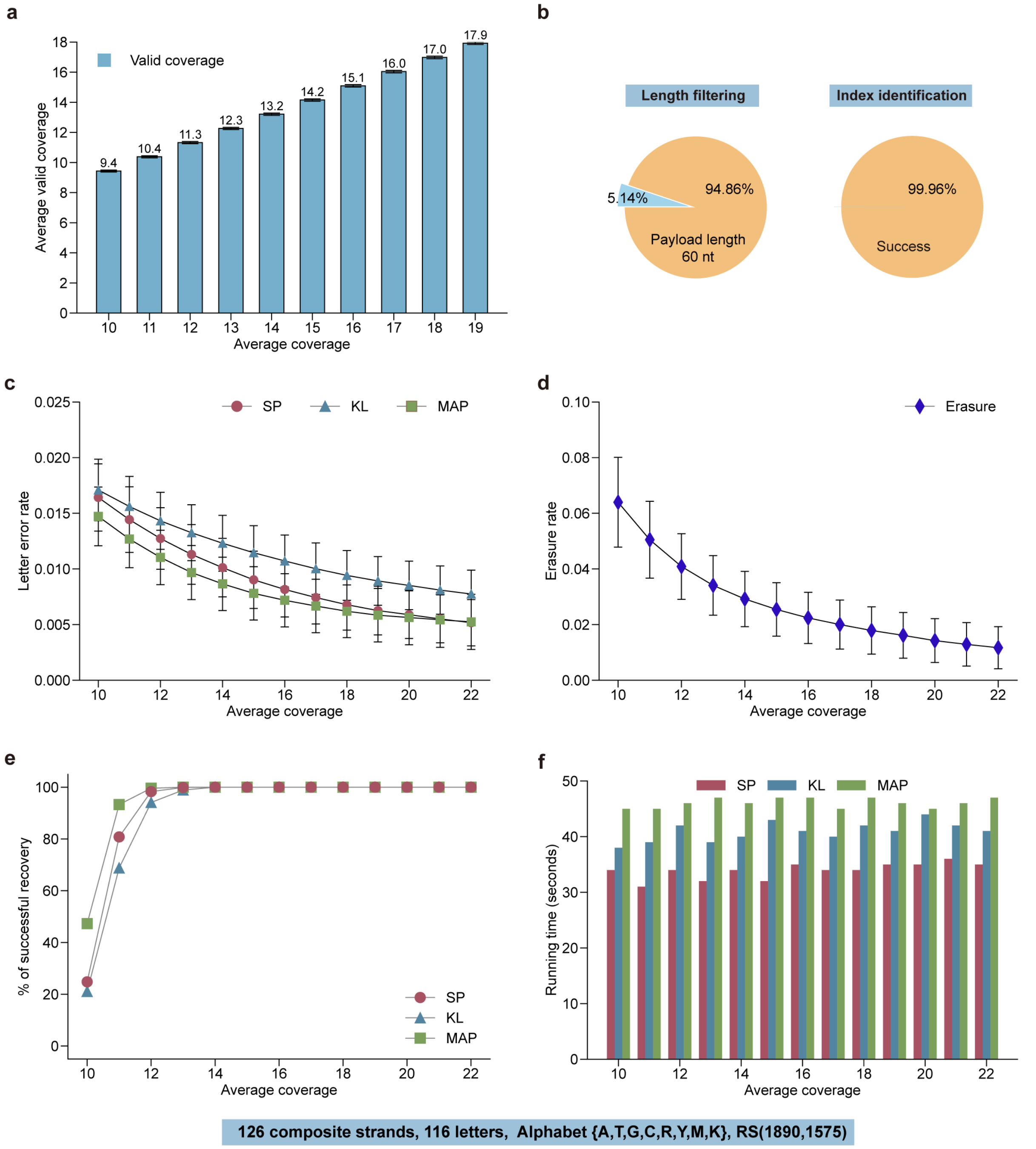

Supplementary Figure 42. End-to-end decoding performance of the eight-letter system (126 strands, 116 letters, RS code, *R*=5/6). a, Relationship between raw sequencing coverage and valid base coverage used for data recovery. **b**, 94.86% of reads passed length filtering and 99.96% were successfully identified by double-end indices. **c**, 99% accuracy at 13× for MAP and 98.9% for SP. **d**, Erasure rate at different average coverages. **e**, Percentage of successful error-free recovery across 1,000 independent trials per coverage. All three methods achieved error-free recovery at 14× coverage. **f**, Runtime comparison of SP, KL, and MAP methods in single-thread mode. Error bars indicate mean ± SD (*n* = 1,000 readout trials per average coverage). Source data are provided as a Source Data file.

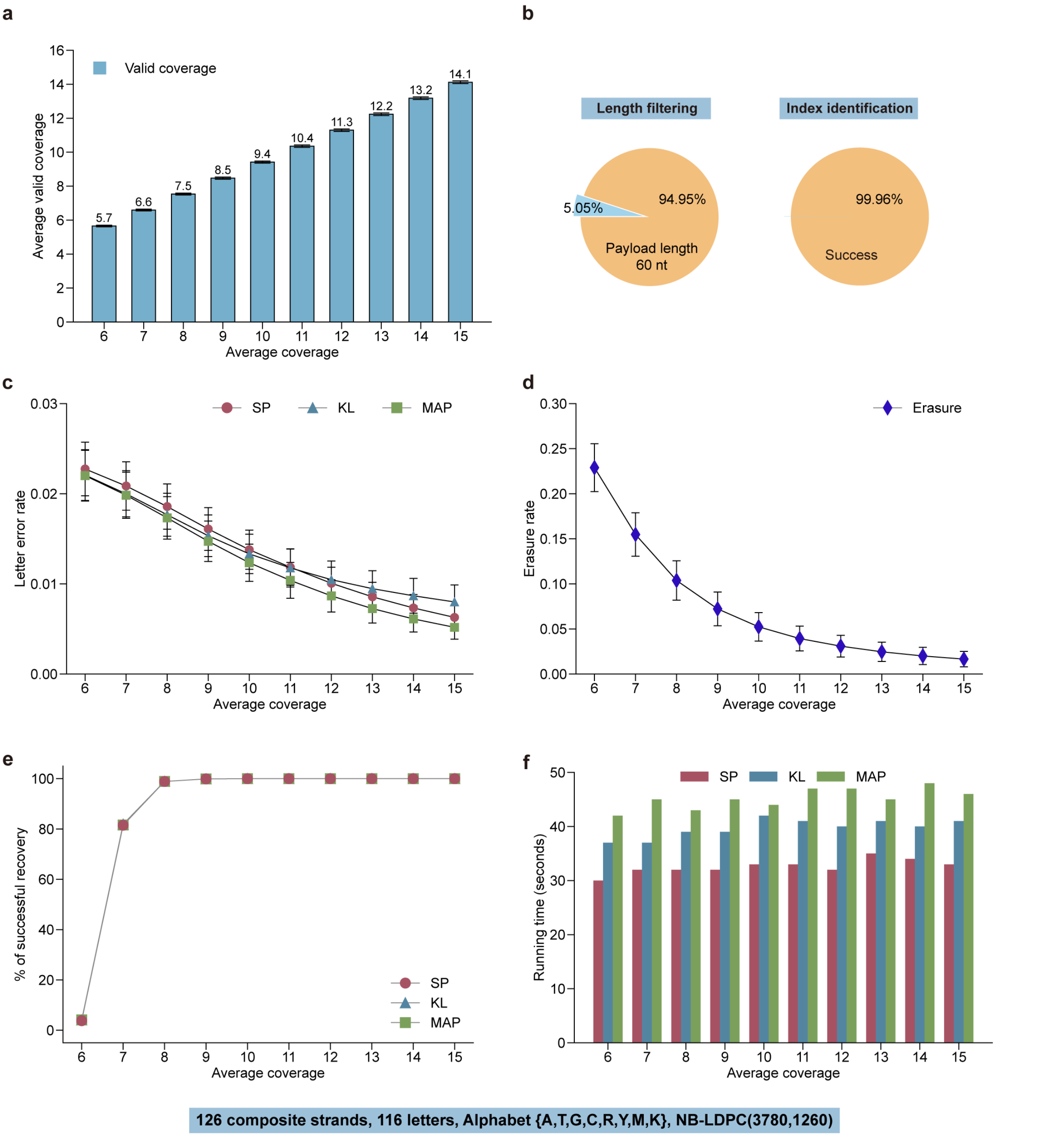

Supplementary Figure 43. End-to-end decoding performance of the eight-letter system (126 strands, 116 letters, NB-LDPC code, *R*=1/3). a, Relationship between raw sequencing coverage and valid base coverage used for data recovery. **b**, 94.95% of reads passed length filtering, and 99.96% were successfully identified by indices. **c**, Letter error rate at different average coverages. **d**, Erasure errors were the major limiting factor for recovery, with the erasure rate exceeding 20% at 6× coverage and remaining as high as 5% at 10×. **e**, Percentage of successful recoveries across 1,000 independent trials. With the strong error-correction capability of the outer code (*R*=1/3), all three methods achieved successful recovery at 9× coverage. **f**, Runtime comparison in single-thread mode, where SP maintained a ~25% runtime advantage over MAP. Error bars indicate mean ± SD (n = 1,000 readout trials per average coverage). Source data are provided as a Source Data file.

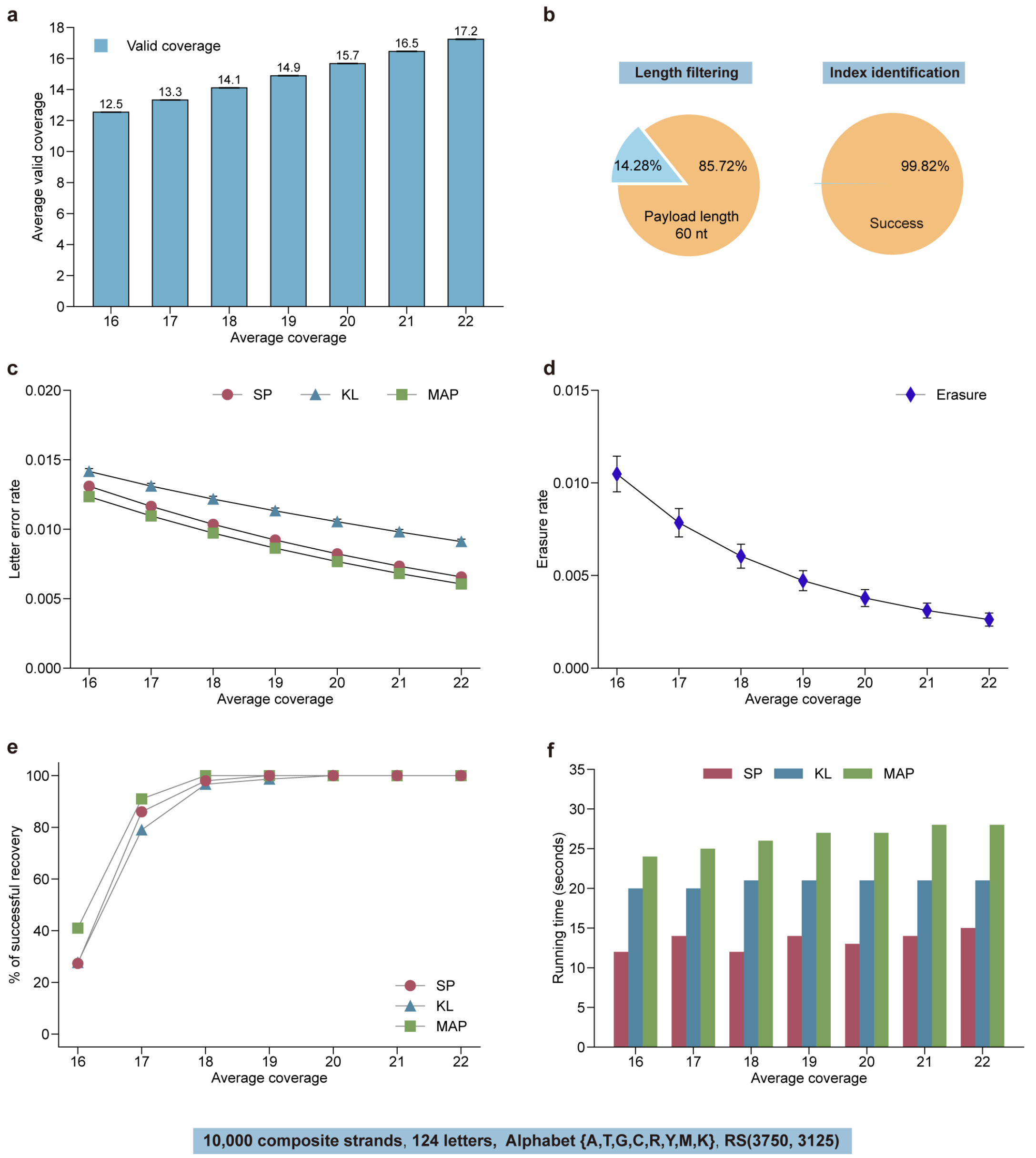

Supplementary Figure 44. End-to-end decoding performance of the eight-letter system (10,000 strands, 124 letters). a, Relationship between raw sequencing coverage and valid base coverage used for composite letter detection and data recovery. **b**, 85.72% of reads passed length filtering and 99.82% were successfully identified by indices. **c**, Letter error rates show that SP closely matches MAP, with both outperforming KL in accuracy. **d**, At the coverage of 17×, the erasure rate was very low (<1%). **e**, Percentage of successful recoveries across 300 independent trials at different coverages. MAP achieved error-free recovery at 18× coverage, while SP, with a slight loss in accuracy, achieved error-free recovery at 19×. **f**, Runtime comparison of SP, KL, and MAP in multi-threaded mode (10 threads), values represent the cumulative runtime of 300 readout trials. SP requires less than 15 s, compared with 20 s for KL and 25 s for MAP. Error bars indicate mean ± SD (*n* = 300 readout trials per average coverage). Source data are provided as a Source Data file.

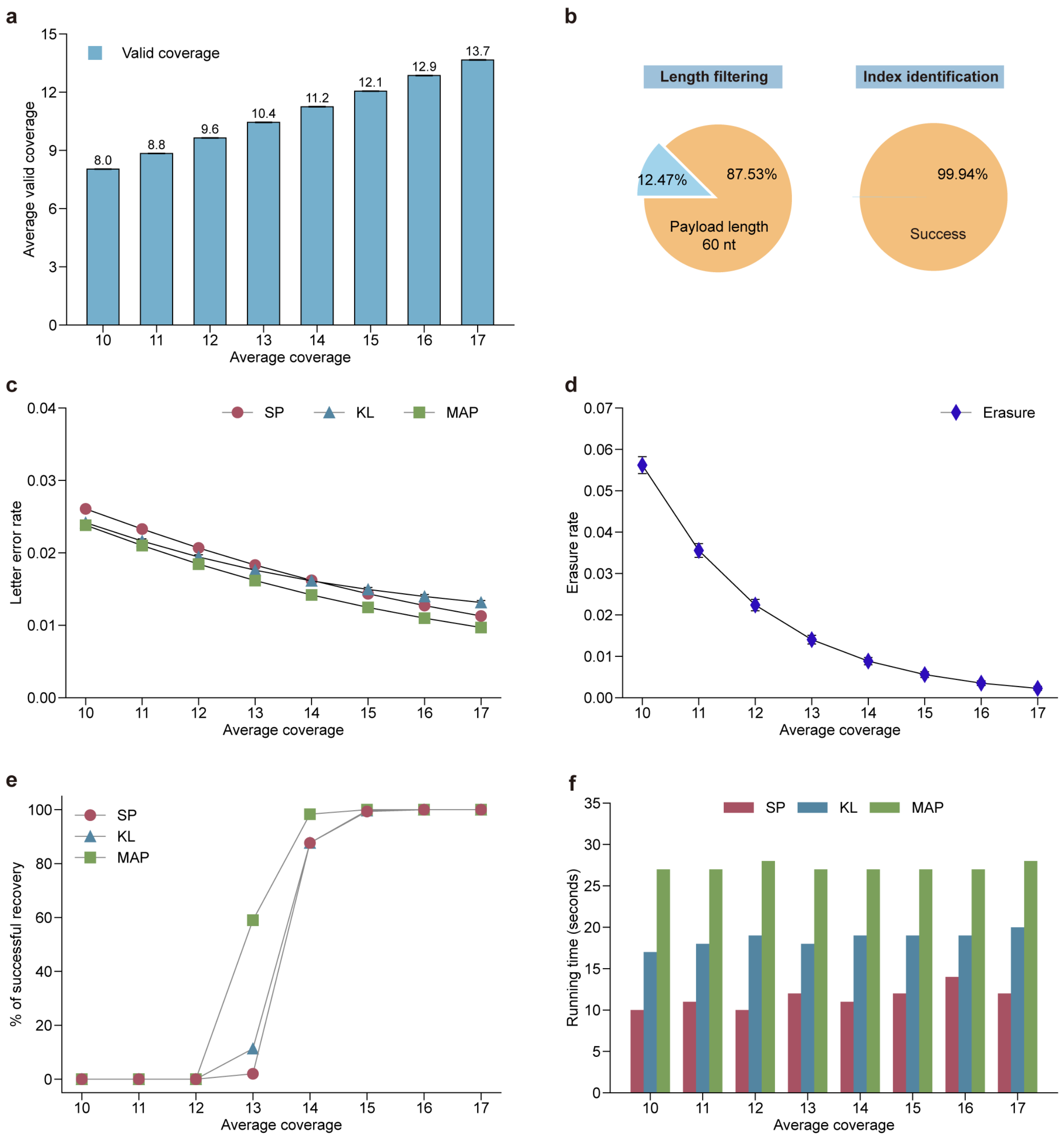

10,000 composite strands, 112 letters, Alphabet {A,T,G,C,R,Y,M,K}, RS(3750, 3125)

Supplementary Figure 45. End-to-end decoding performance of the eight-letter system (10,000 strands, 112 letters). a, Relationship between raw sequencing coverage and valid base coverage used for data recovery. **b**, 87.53% of reads passed length filtering and 99.94% were successfully identified by indices. **c**, Detection accuracy was comparable among the three methods, with MAP performing the best. **d,** Erasure rates decreased below 0.5% by 16× coverage. **e,** Error-free recovery was achieved by MAP at 15×, and by SP and KL at 16× coverage, based on 300 independent trials. **f,** SP completed 300 trials in ~12 s, whereas MAP required ~25 s. Error bars indicate mean ± SD (*n* = 300 readout trials per average coverage). Source data are provided as a Source Data file.

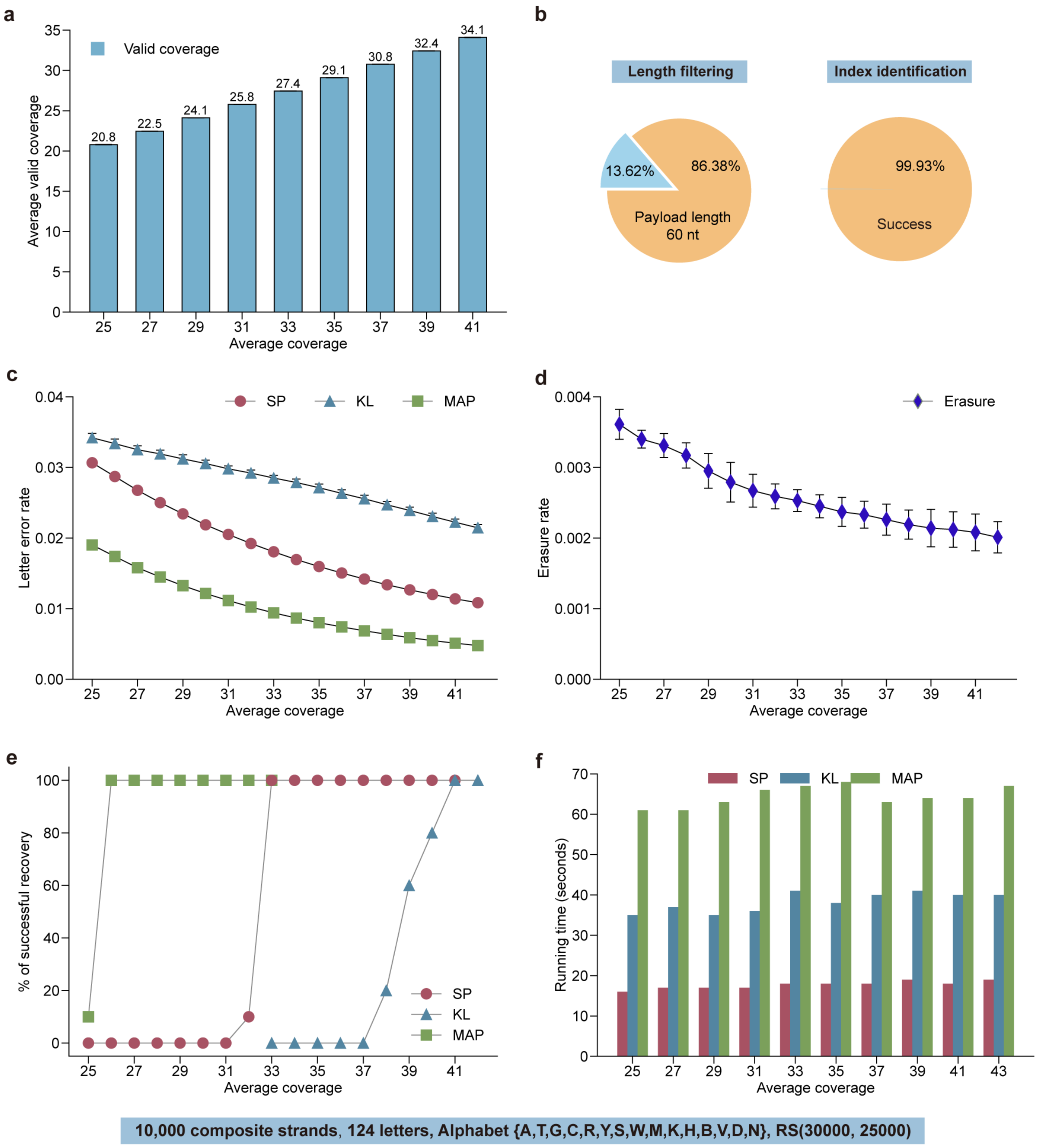

Supplementary Figure 46. End-to-end decoding performance of the 15-letter system (10,000 strands, 124 letters). a, Relationship between raw sequencing coverage and valid base coverage used for data recovery. **b**, 86.38% of reads passed length filtering and 99.93% were successfully identified by indices. **c,** With larger alphabets, SP showed higher error rates than MAP. **d,** At the coverage ≥25×, erasure errors were nearly absent. **e,** Percentage of successful recoveries across 300 independent trials at each coverage. Error-free recovery was achieved at 26× coverage using MAP, whereas 33× coverage was required for SP. **f,** Runtime comparison for 300 trials: SP completed letter detection in ~18 s, compared with ~35 s for the KL method and >60 s for the MAP method. Error bars indicate mean ± SD (*n* = 300 readout trials per average coverage). Source data are provided as a Source Data file.

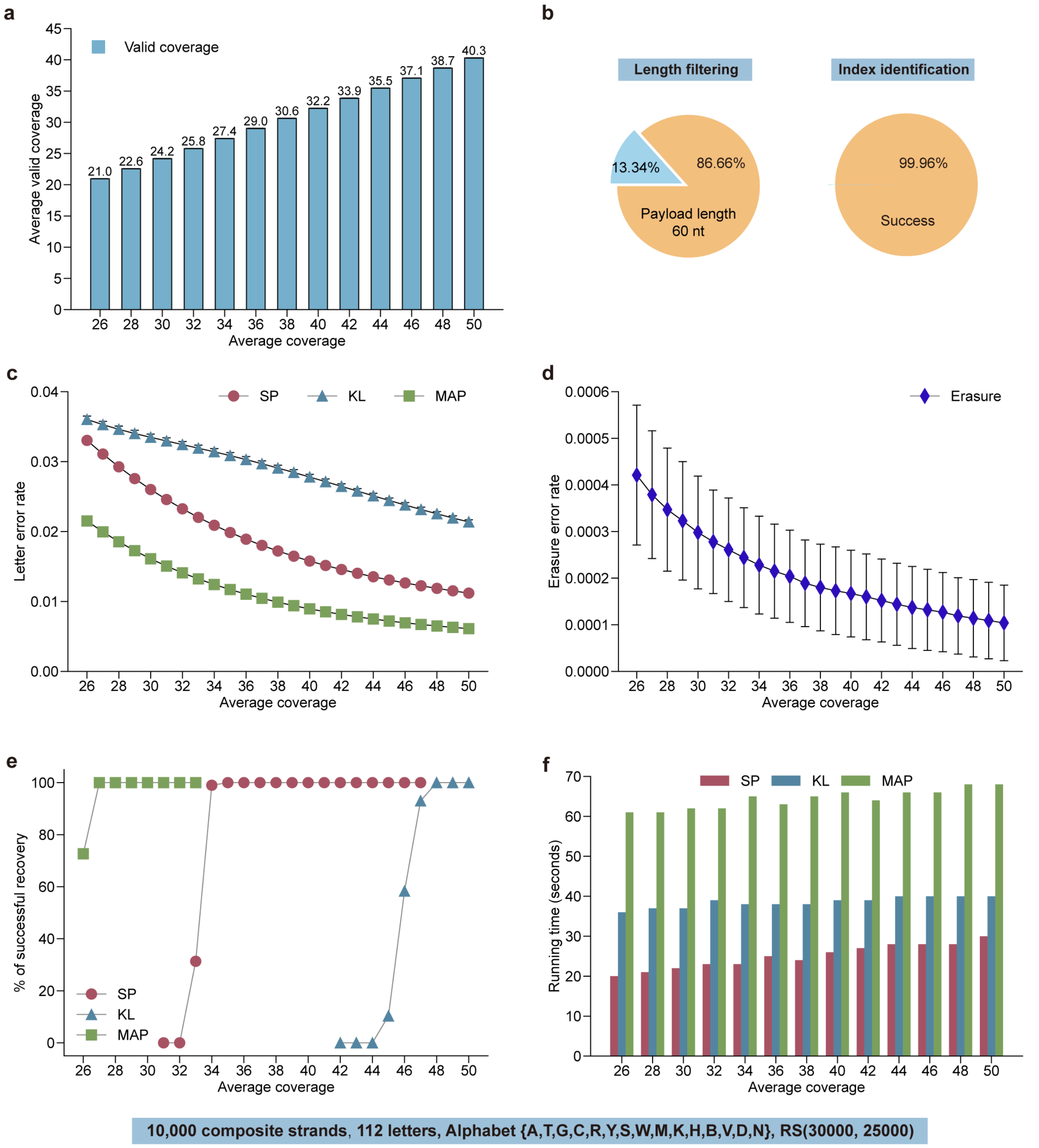

Supplementary Figure 47. End-to-end decoding performance of the 15-letter system (10,000 strands, 112 letters). a, Relationship between raw sequencing coverage and valid base coverage used for data recovery. **b**, 86.66% of reads passed length filtering and 99.96% were successfully identified by indices. **c,** With larger alphabets, SP showed higher error rates than MAP. **d,** At the coverage ≥26×, erasure errors were nearly absent. **e,** Percentage of successful recoveries across 300 independent trials at each coverage. Error-free recovery was achieved at 27× coverage using MAP, whereas 35× coverage was required using SP and 48× with the KL method. **f,** Runtime comparison for 300 trials: SP completed letter detection in ~20 s, compared with ~35 s for the KL method and ~60 s for the MAP method. Error bars indicate mean ± SD (*n* = 300 readout trials per average coverage). Source data are provided as a Source Data file.

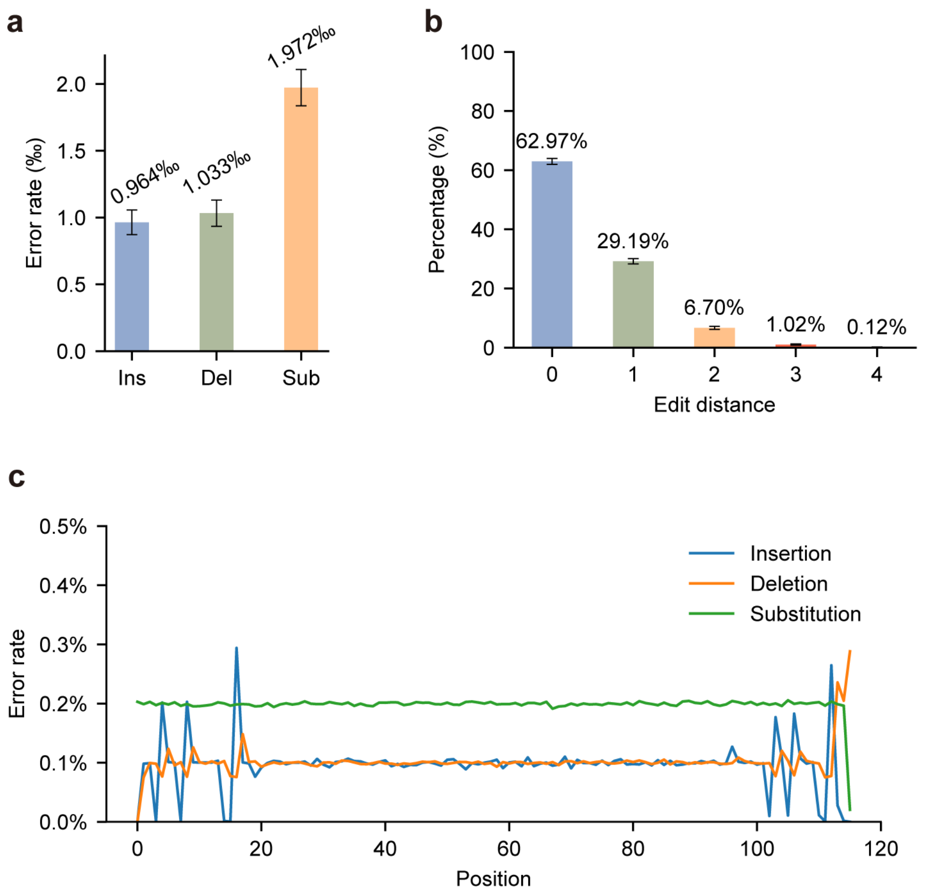

Supplementary Figure 48. Benchmarking the stochastic error model used in simulations. **a**, Mean error rate of insertions (Ins.), deletions (Del.) and substitutions (Sub.) produced by the simulator. The channel was parameterized with target error probabilities of 0.1% (Ins.), 0.1% (Del.), and 0.2% (Sub.). **b**, Distribution of edit distance, that is, the total number of indel errors per read. Most reads are error-free (63 %), 29% carry a single indel, 6.7% carry two, and < 1% contain more than two. **c**, Position-resolved error rates along 116-nt templates. Substitution frequency (green) is essentially uniform at ~0.2% across the sequence, while insertion (blue) and deletion (orange) rates remain flat at ~0.1%, indicating negligible positional bias in the simulated reads. Error bars indicate mean ± SD (n = 1,000 simulation trials). Source data are provided as a Source Data file.

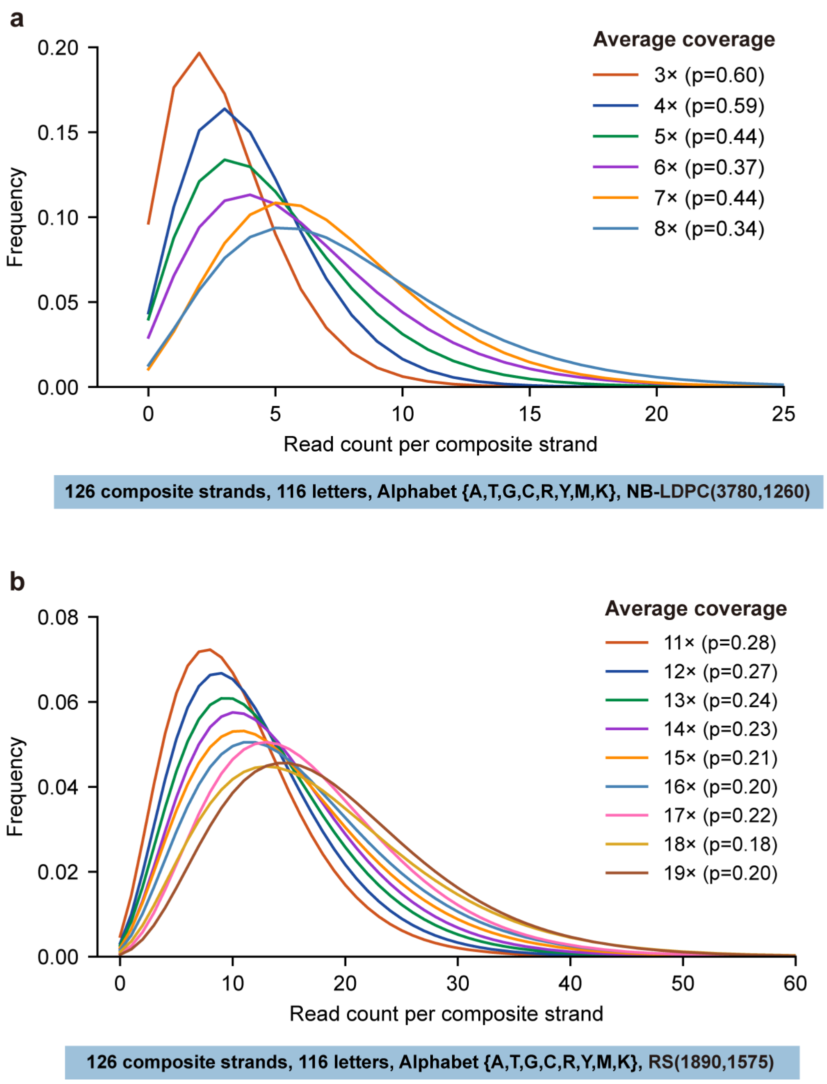

Supplementary Figure 49. Coverage distribution in simulations. a and b, For every simulation experiment, we assigned a read coverage to each DNA strand by sampling from a negative-binomial distribution. Curves show the resulting probability density of observing a given coverage (x-axis). The frequency at a coverage of zero represents molecules that were never sequenced, modeled as erasures for the decoder. The nature of these distributions, many reads for some strands and few or none for others, mirrors experimental bias and makes letter detection challenging at low coverage. Source data are provided as a Source Data file.

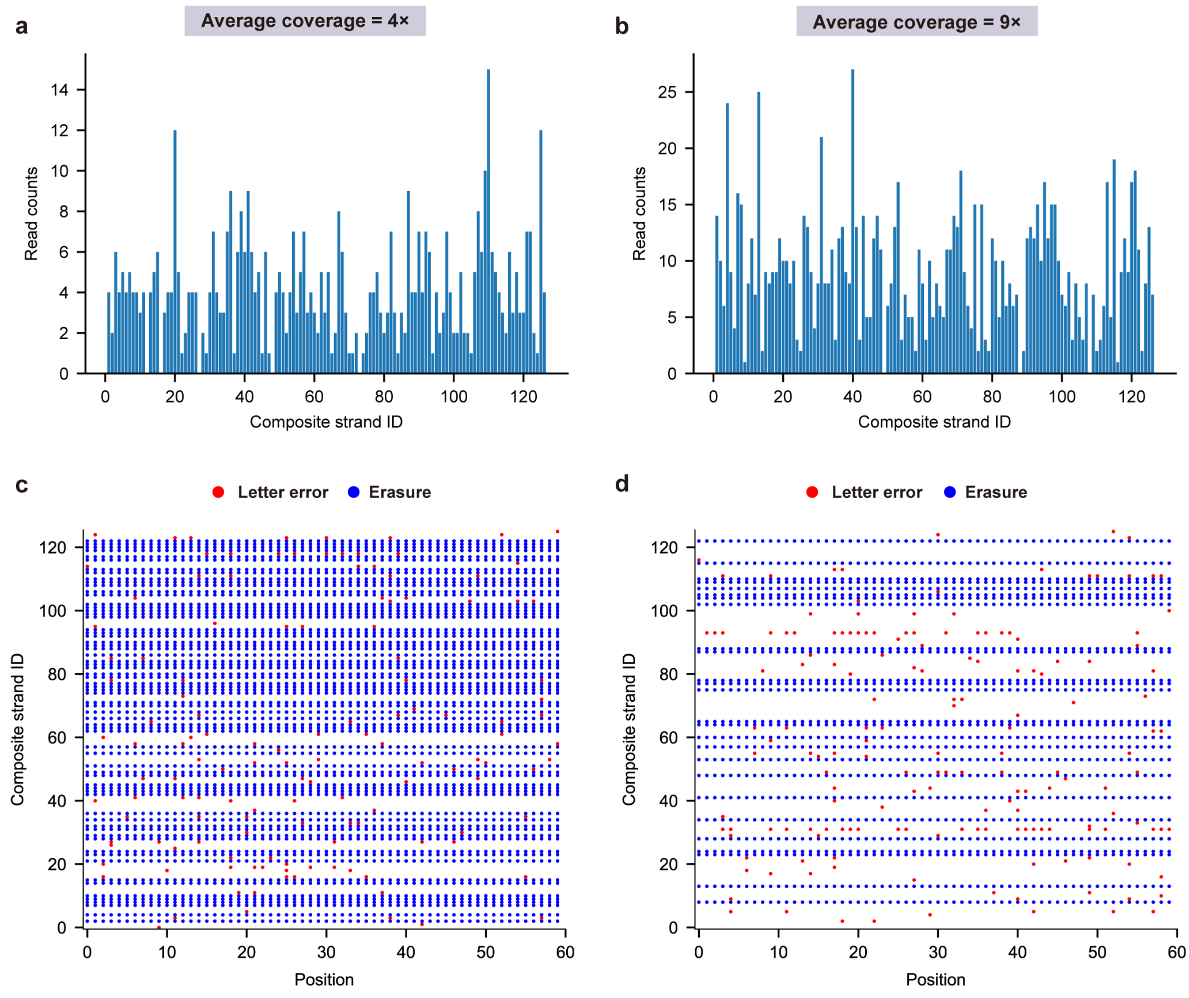

Supplementary Figure 50. Simulated copy-number heterogeneity and pre-decoding error distribution for LDPC-protected composite strands. a and b, Per-strand read copies obtained from the negative-binomial sequencing model at average coverages of 4× and 9×. Each blue dot represents one of the 126 composite strands in the pool. A value of zero denotes a strand that was never observed and is therefore treated as an erasure by the decoder. **c and d,** Error maps after our two-stage letter-detection method but before LDPC decoding. At 4× average coverage, many erasures and letter errors remain, whereas at 9×, the error load is still substantial yet lies within the correction capability of the high-redundancy LDPC (3780, 1260) code, enabling error-free data recovery in subsequent decoding. Source data are provided as a Source Data file.

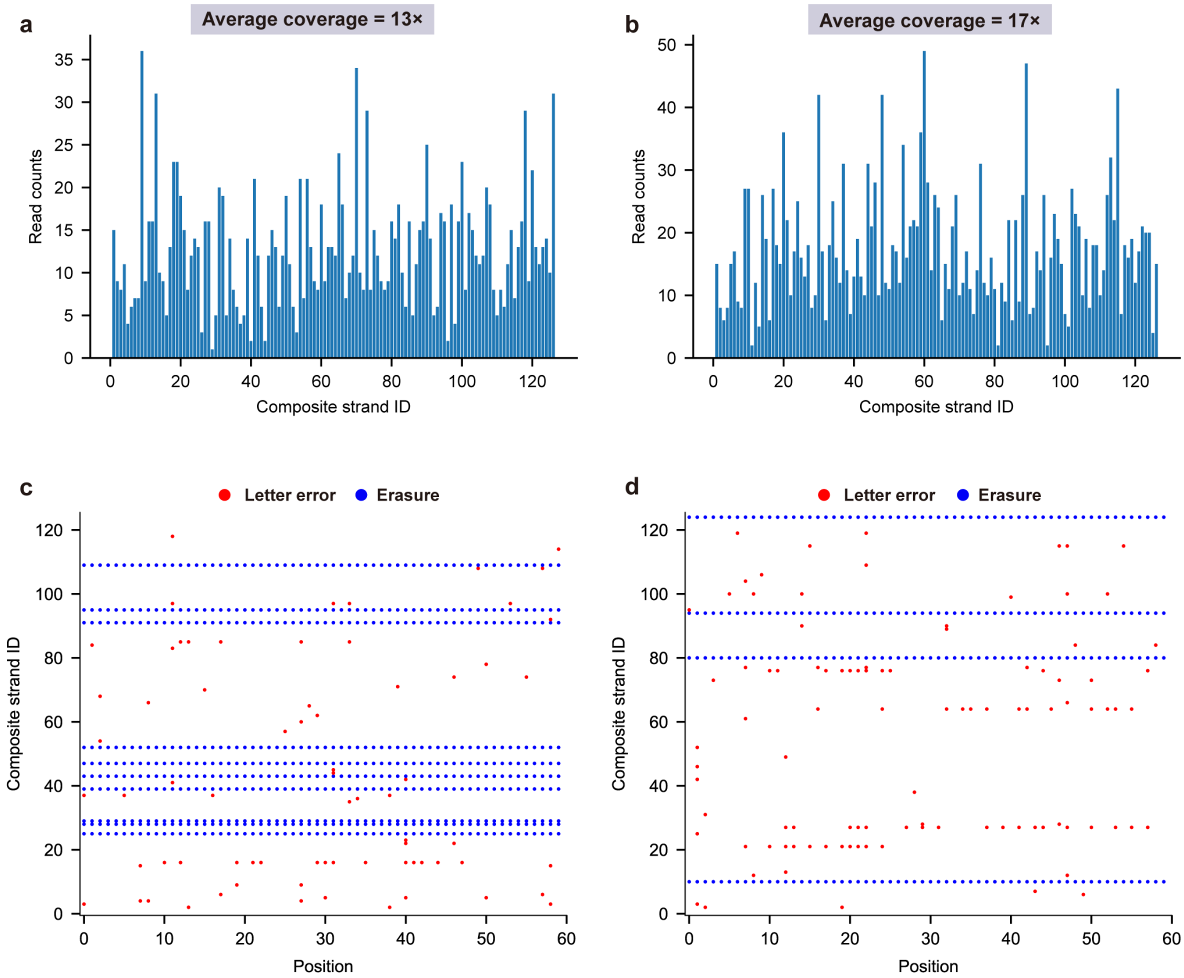

Supplementary Figure 51. Simulated copy-number heterogeneity and pre-decoding error distribution for RS-protected composite strands. a and b, Per-strand read counts for the Reed–Solomon pool at average coverages of 13× and 17×. The coverage bias shows up as some strands being read about 30×, while others are completely absent (coverage = 0), generating erasures that the decoder must correct. **c and d**, Pre-decoding error maps generated with the same two-stage letter detector. At 17× average coverage, the residual error burden is within the correction reach of the RS(1890, 1575) code. Compared with the LDPC-protected pool, the highly efficient code demands considerably deeper coverage to overcome the combined effects of strand dropout and uneven read coverage. Source data are provided as a Source Data file.

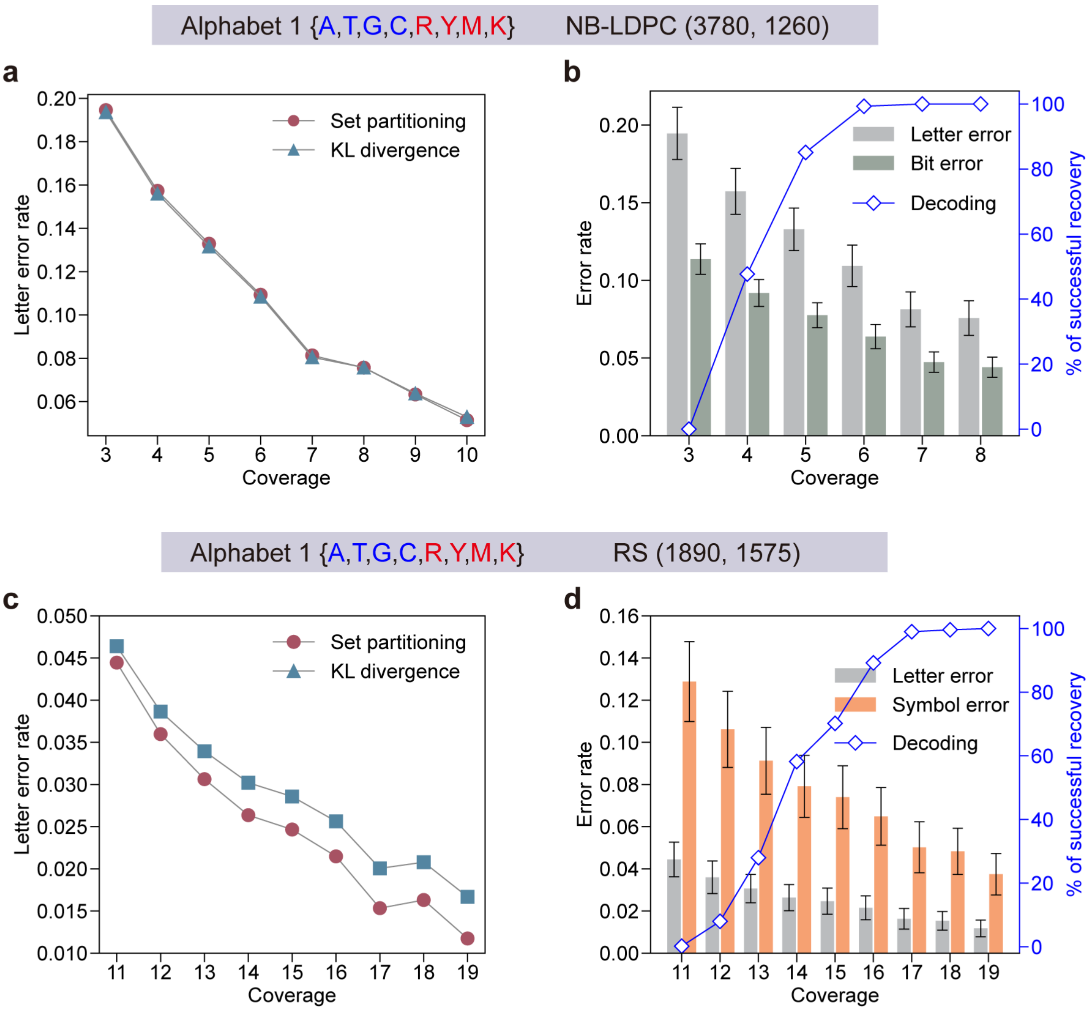

Supplementary Figure 52. Data recovery tests at various sequencing coverages based on simulated sequencing reads for two pools using alphabet 1 {A, T, G, C, R, Y, M, K}. a, Letter error rate produced by the two different letter methods. **b**, Residual errors and data recovery for the LDPC-protected pool. As sequencing coverage decreased, both letter and bit errors increased. When the coverage dropped to 3×, the error rate was about 20%, surpassing the error-correcting capacity of the chosen LDPC code. Error-free data recovery is achieved in all 1,000 simulation runs at a coverage of 7×. Error bars indicate mean ± SD (n = 1,000 readout trials per coverage). **c**, In the RS-protected pool, our set partitioning method delivers a modest yet consistent gain in letter-detecting accuracy over the conventional KL-divergence method throughout the 11× to 19× coverage range. **d**, At 19× coverage, all 1,000 Monte Carlo tests using the RS(1890, 1575) coding scheme resulted in error-free data recovery, consistent with the success rate observed in physical experiments. Error bars indicate mean ± SD (n = 1,000 readout trials per coverage). Because the RS code carries far less redundancy than the LDPC code, it demands higher sequencing coverage, yet it compensates with a correspondingly higher storage density. Source data are provided as a Source Data file.

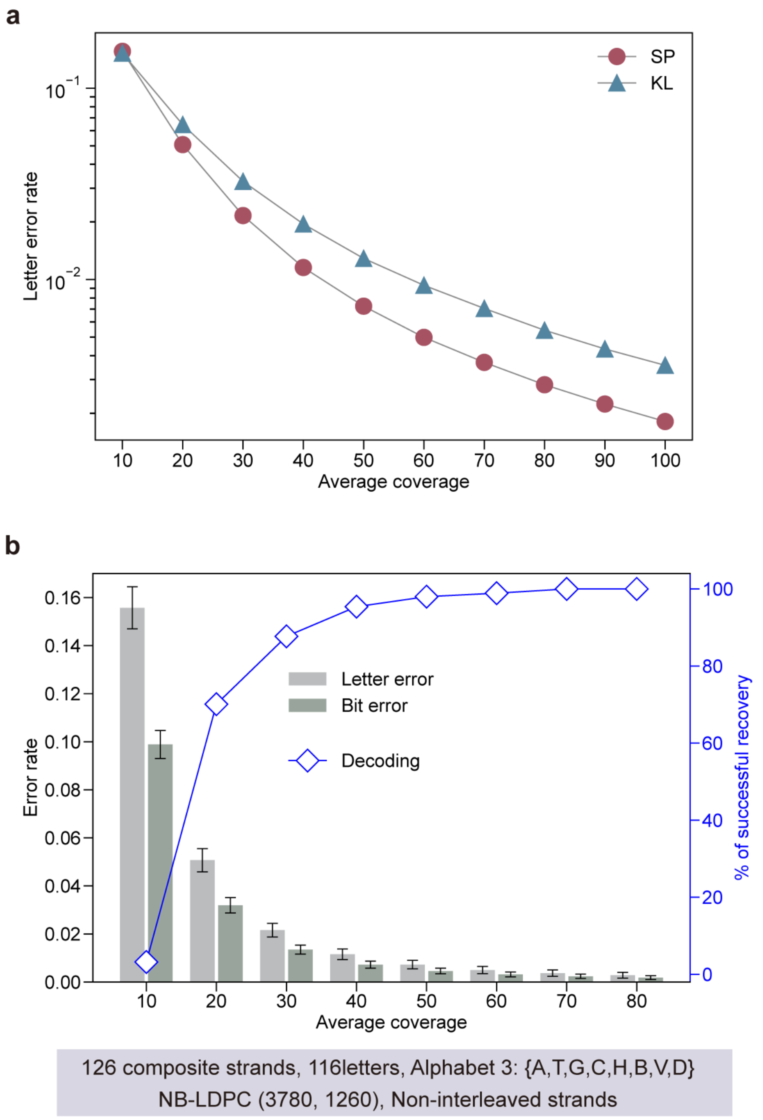

Supplementary Figure 53. Data recovery performance for Alphabet 3 {A, T, G, C, H, B, V, D} using a non-interleaved scheme. a, Letter-error rate versus sequencing coverage. The proposed set partitioning detection method (circles) increasingly outperforms the conventional KL divergence method (triangles) as coverage rises, demonstrating superior letter-detecting accuracy. **b**, Decoding results based on a non-interleaved scheme. Symbol interleaving disperses consecutive erasures across the entire codeword, thereby avoiding susceptibility to strand dropouts. Error bars indicate mean ± SD (n = 1,000 readout trials per coverage). Compared with the results obtained using Alphabet 1 (with symbol interleaving), without symbol interleaving, the non-interleaved scheme exhibits poor tolerance to erasures caused by molecule loss, necessitating substantially higher sequencing coverage (70×) for error-free data recovery. Source data are provided as a Source Data file.

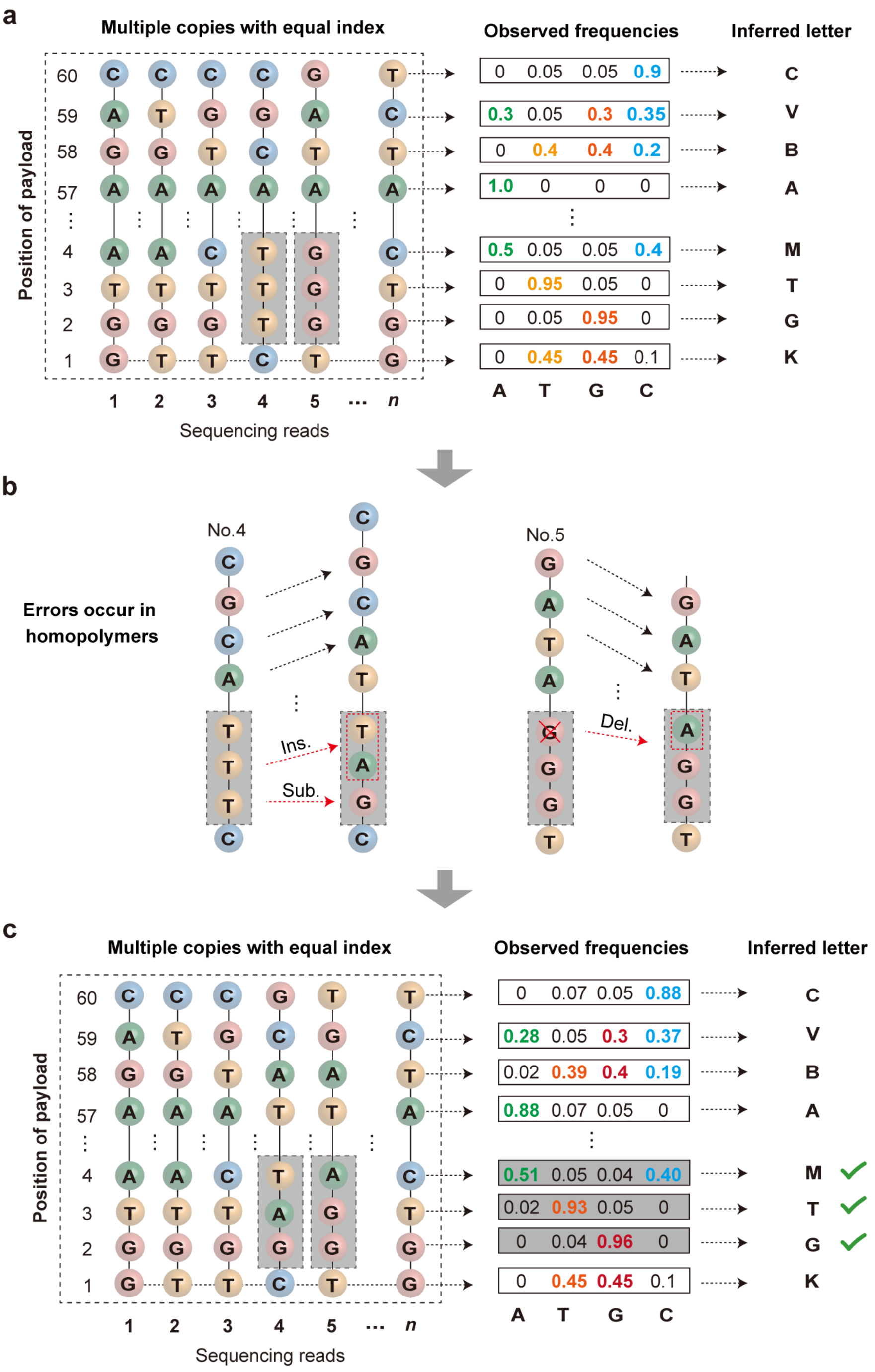

Supplementary Figure 54. Frequency-based inference of composite letters. a, Multiple sequencing reads with the same indices are aligned, and the observed base frequencies at each synthesis site are used to infer the most probable composite letter. **b**, Indel errors in homopolymer regions cause subsequent bases to misalign. **c**, Composite letters are detected based on frequency distributions from multiple copies, which offset the effect of local errors.

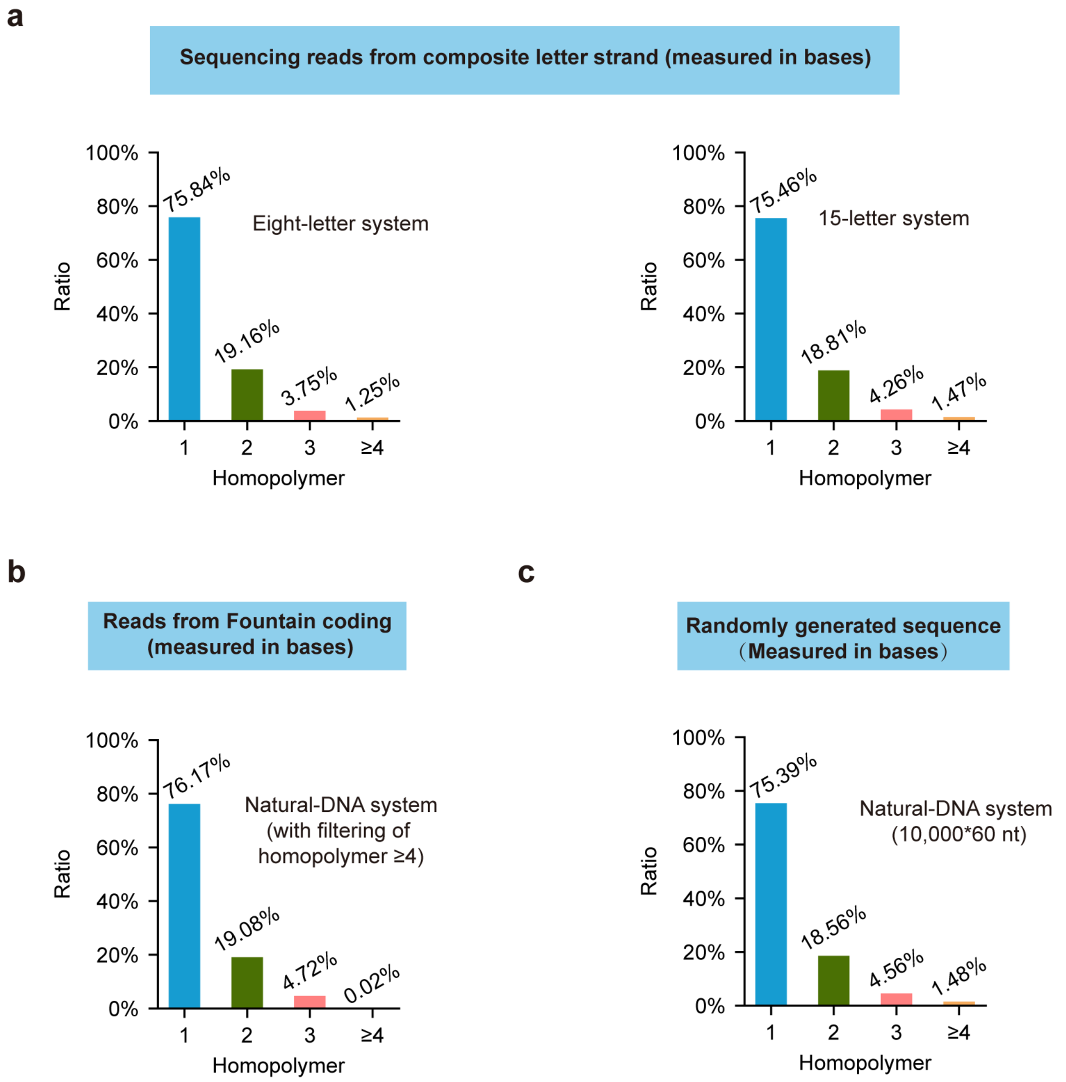

Supplementary Figure 55. Homopolymer length distribution. a, Sequencing reads from composite letter strands (measured in bases). The proportions of single-base homopolymers are 75.84% in the 8-letter system and 75.46% in the 15-letter system. **b**, Sequencing reads from a Fountain coding–based natural DNA system (measured in bases), with 75.90% single-base homopolymers. **c**, Randomly generated sequences. The proportion of single-base homopolymers is 75.39% in the natural-DNA system (measured in bases). Source data are provided as a Source Data file.

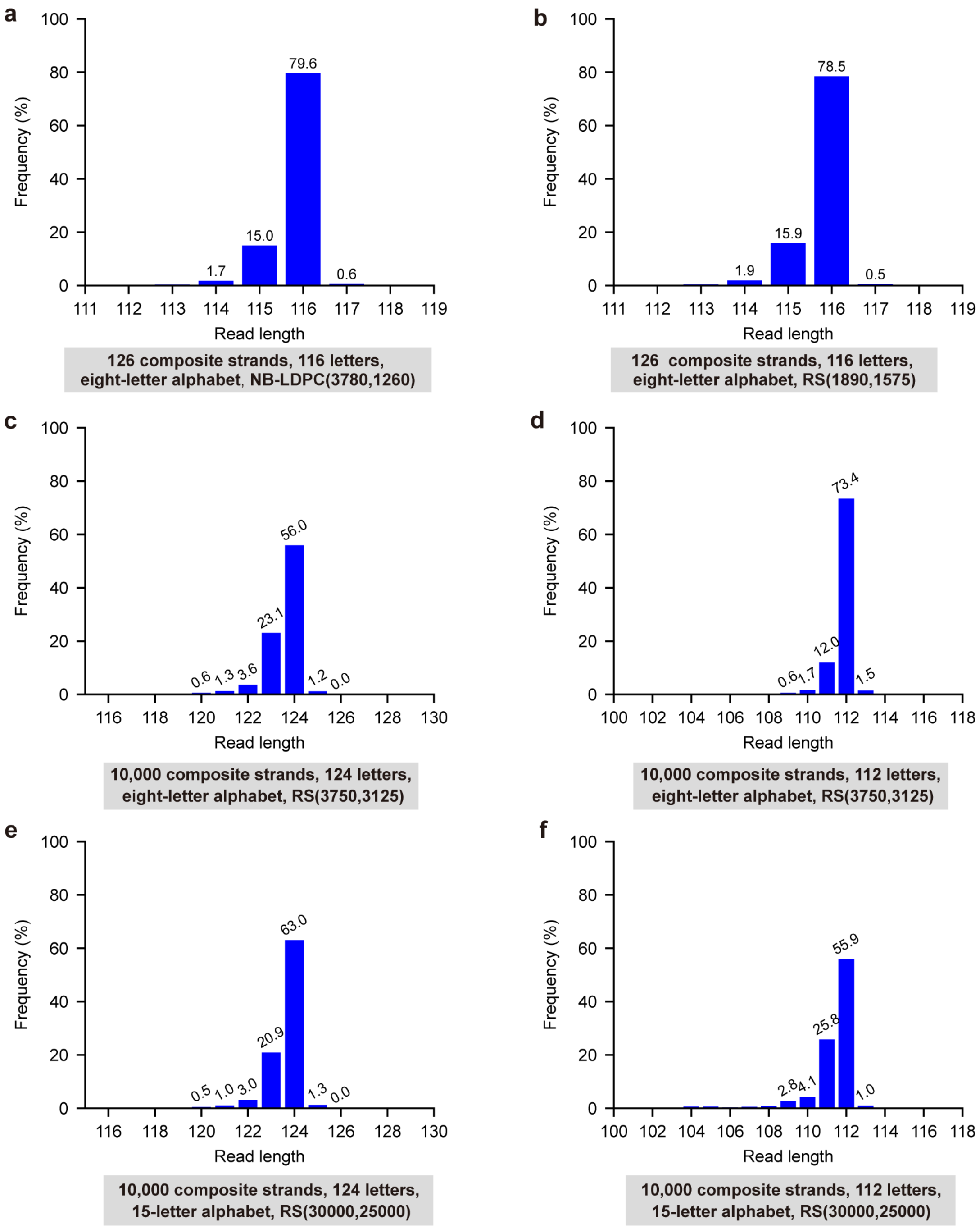

Supplementary Figure 56. Length distribution of assembled sequencing reads. a and b, Length distributions of 126 composite strands. The strands were encoded using either the NB-LDPC(3780, 1260) code or the RS(1890, 1575) code, with 79.6% and 78.5% of sequencing reads, respectively, reaching the designed full length of 116 nt. **c and d**, Large-scale composite pools using the eight-letter alphabet, where 56.0% and 73.4% of reads, respectively, reached the designed full length after paired-end assembly. **e and f**, Large-scale composite pools using the fifteen-letter alphabet, where 63.0% and 55.9% of reads, respectively, reached the designed full length after paired-end assembly. Source data are provided as a Source Data file.

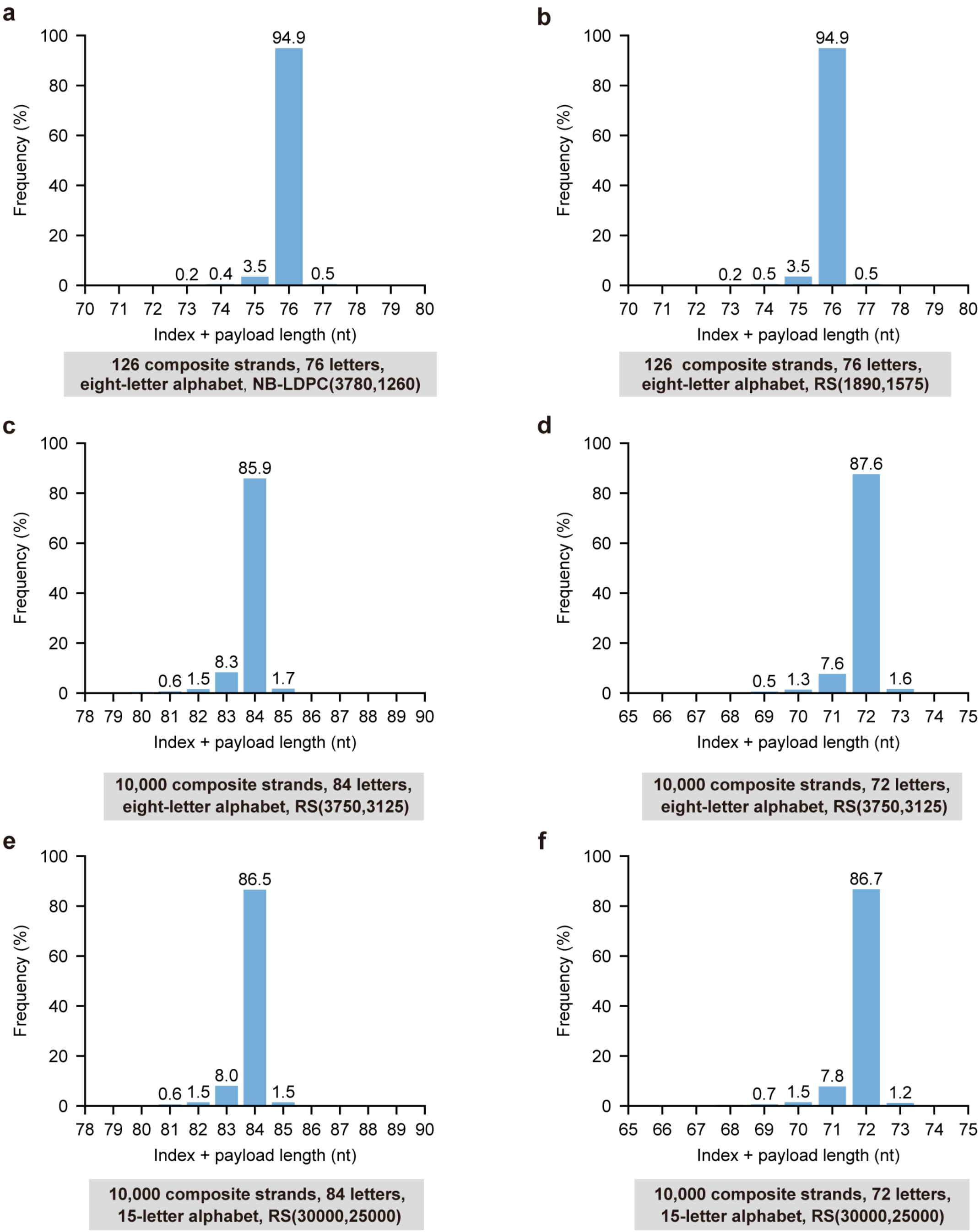

Supplementary Figure 57. Length distributions of index and payload regions after primer identification. a and b, Both datasets exhibited nearly identical distributions, with 94.9% of reads retaining the designed full length (76 nt) after primer trimming. **c and d,** Two large-scale pools using the eight-letter alphabet, with 85.9% (84 nt) and 87.6% (72 nt) of reads, respectively, retaining the designed payload length. **e and f,** Two large-scale pools using the fifteen-letter alphabet, with 86.5% (84 nt) and 86.7% (72 nt) of reads, respectively, retaining the designed payload length. Source data are provided as a Source Data file.

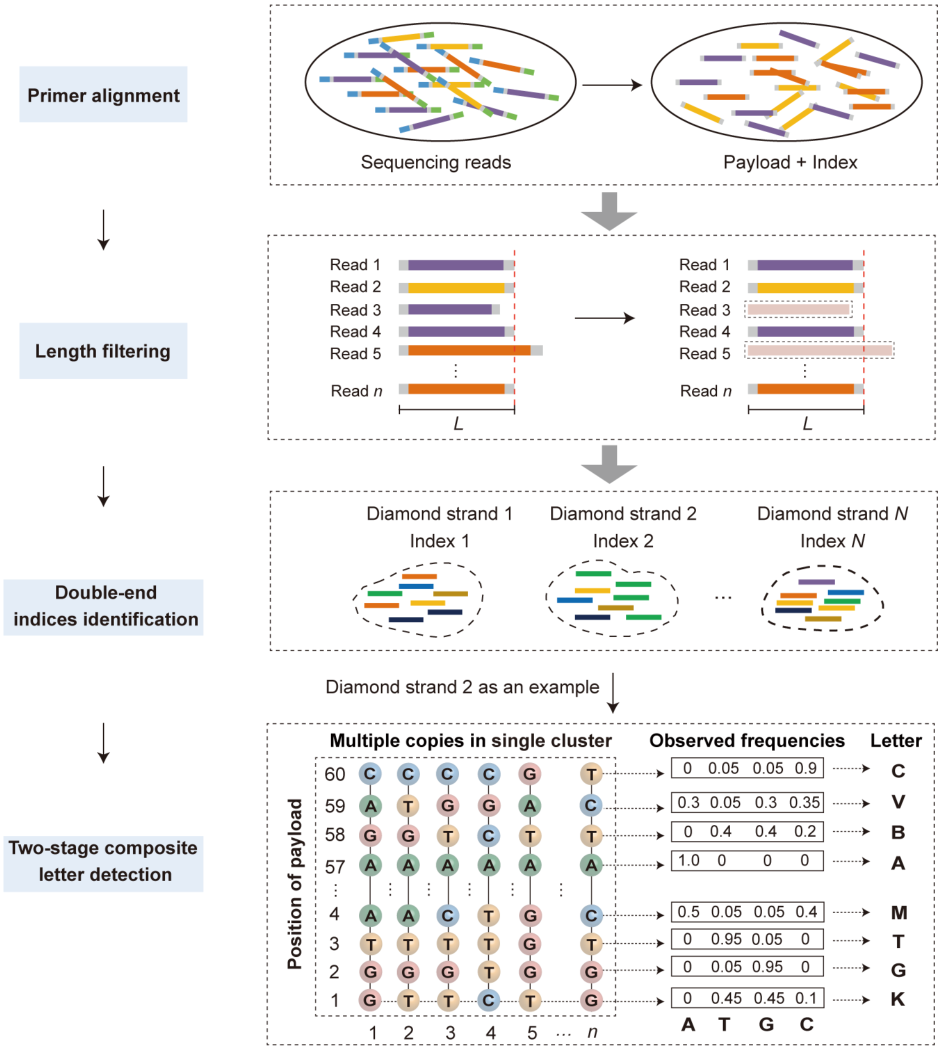

Supplementary Figure 58. Readout workflow with length filtering. Sequencing reads are first aligned to double-end primers to identify payload and index segments (Primer alignment). Reads with abnormal length are discarded, ensuring equal-length, aligned sequences for downstream readout (Length filtering). Reads are then grouped into different clusters according to the index sequences (Index identification). Within each cluster, base frequency distributions at each position are calculated across multiple copies, and the most probable composite letter is inferred (Letter detection). Source data are provided as a Source Data file.

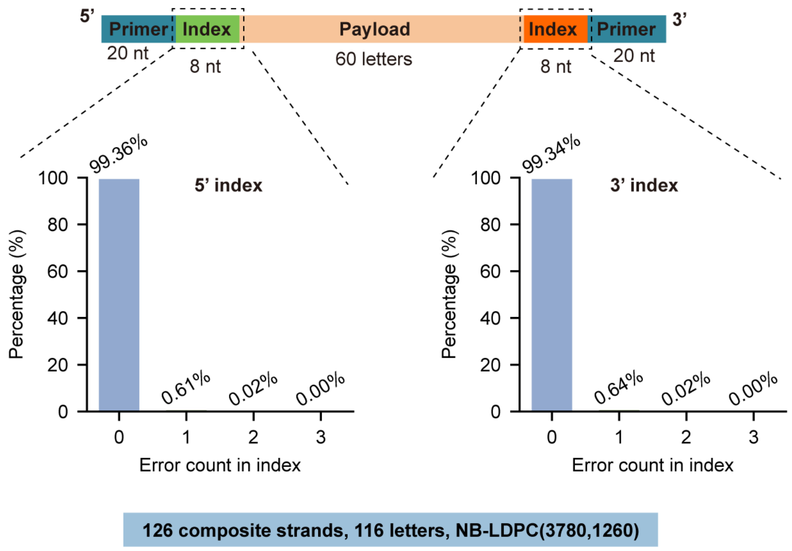

Supplementary Figure 59. Error distribution in double-end indices. Analysis shows that 99.3% of indices are error-free, demonstrating high reliability for index identification. Source data are provided as a Source Data file.

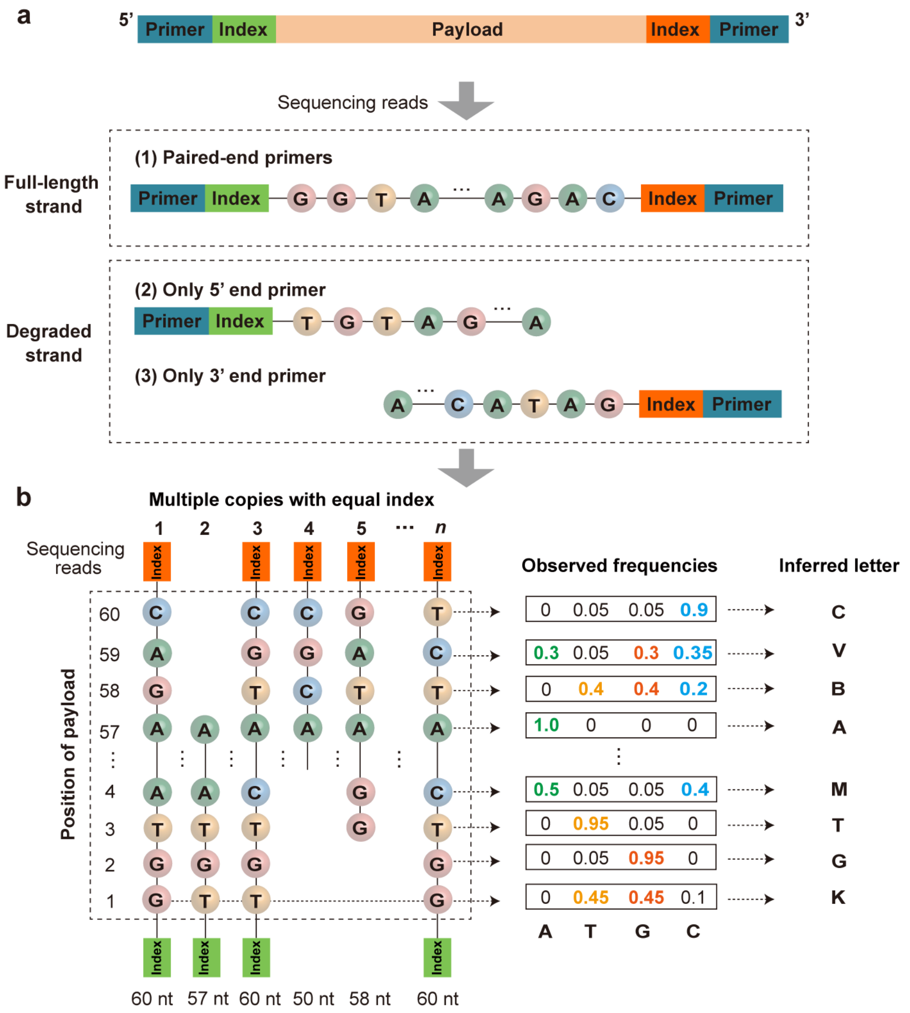

Supplementary Figure 60. Design of composite DNA strand structure enabling degradation-tolerant decoding. a, Schematic of composite strand architecture with double-end primers and indices. Three sequencing scenarios are shown: (1) reads containing both paired-end primers, (2) reads containing only the 5' primer, and (3) reads containing only the 3' primer. **b**, Multiple sequencing reads sharing the same index are aggregated to compute position-wise observed frequencies and infer composite letters. Even if one end of the strand is degraded, the remaining indexed fragment contributes to composite letter detection.

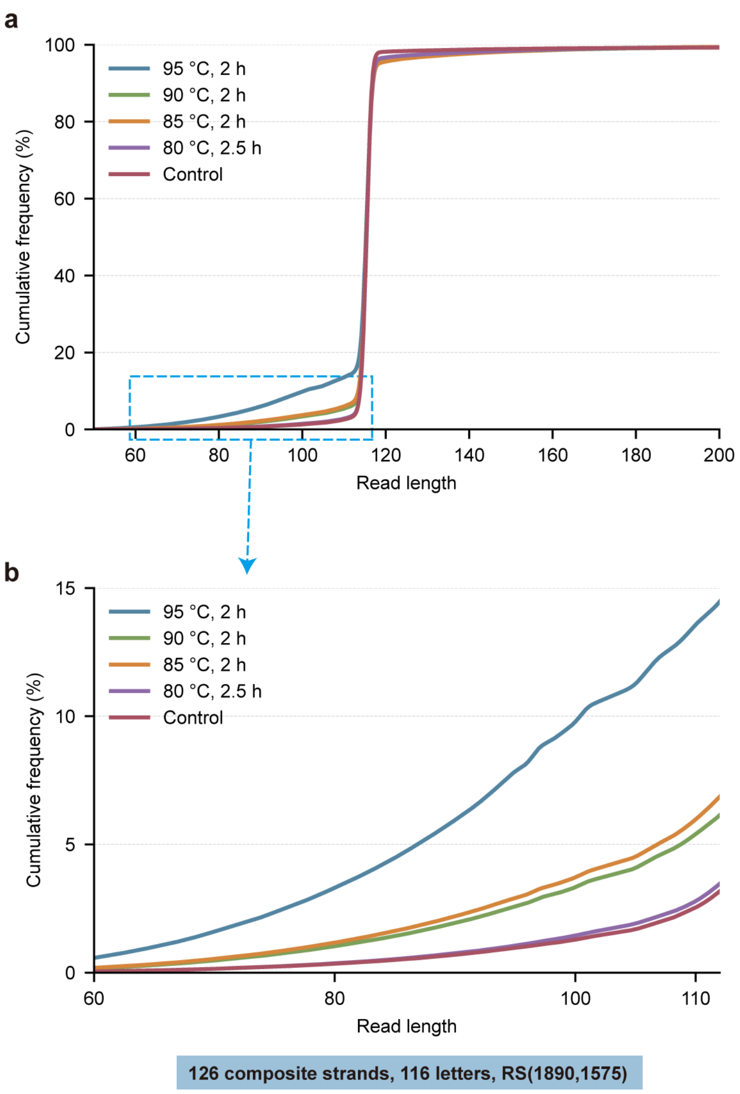

Supplementary Figure 61. Cumulative distributions of read length under different degradation conditions. a, Cumulative frequency curves of assembled reads for the pool encoded with RS(1890, 1575). Increasing temperature led to a gradual shift toward shorter read lengths and a broader cumulative distribution, indicating progressive degradation of DNA strands. **b**, Magnified view of the 60–110 nt region. The original full-length reads were 116 nt in design, and the detailed view highlights the emergence of shorter degraded fragments at higher temperatures (95 °C, 2 h). Source data are provided as a Source Data file.

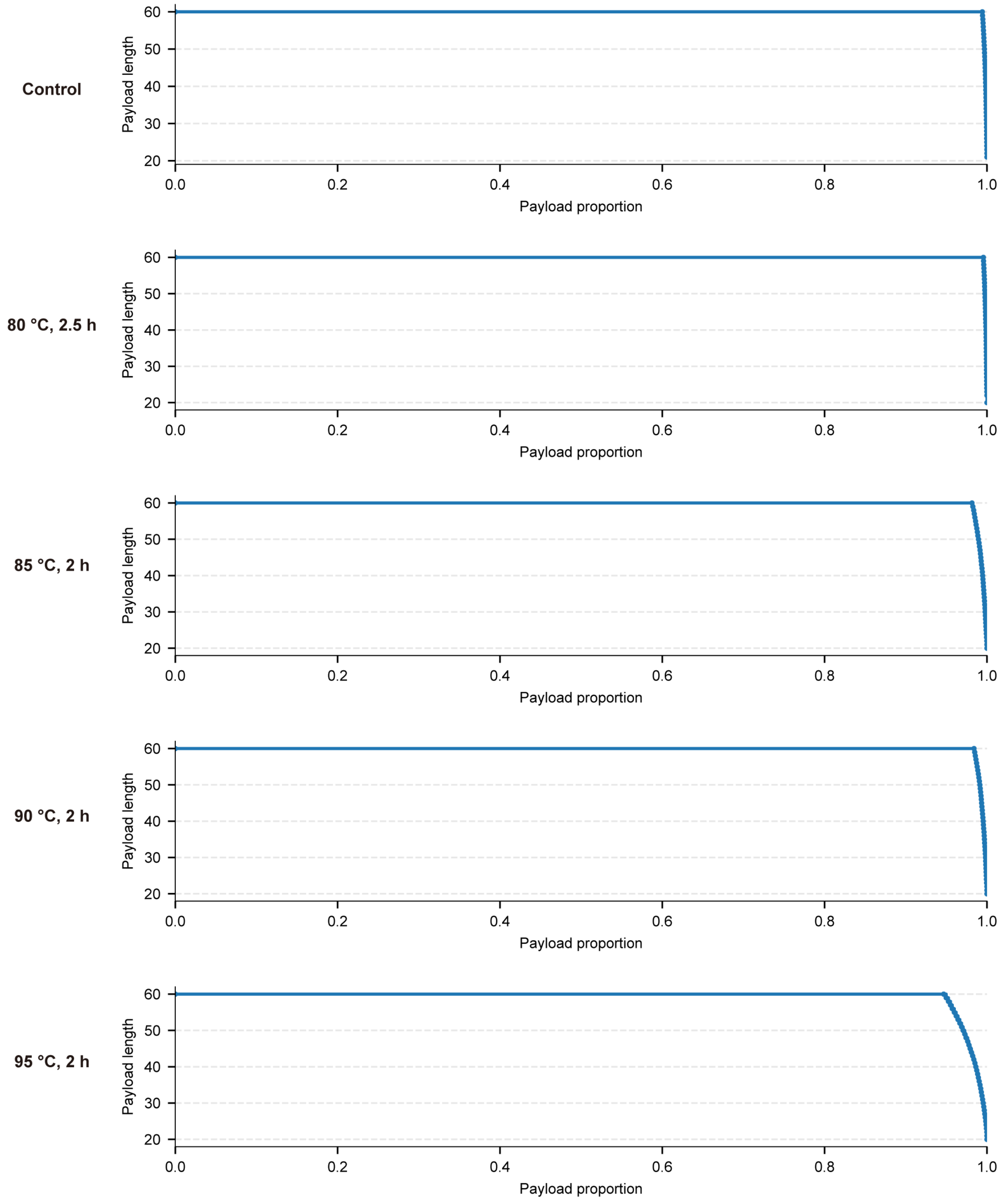

Supplementary Figure 62. Proportion of read length under different thermal degradation conditions (only payload). The *y*-axis is payload length (nt), and the *x*-axis is the proportion of reads when strands are sorted by payload length from long to short; i.e., for each proportion on the *x*-axis, the curve shows the corresponding payload length. Higher temperatures (95 °C, 2 h) shift the curves downward, indicating a larger fraction of shorter payloads. Source data are provided as a Source Data file.

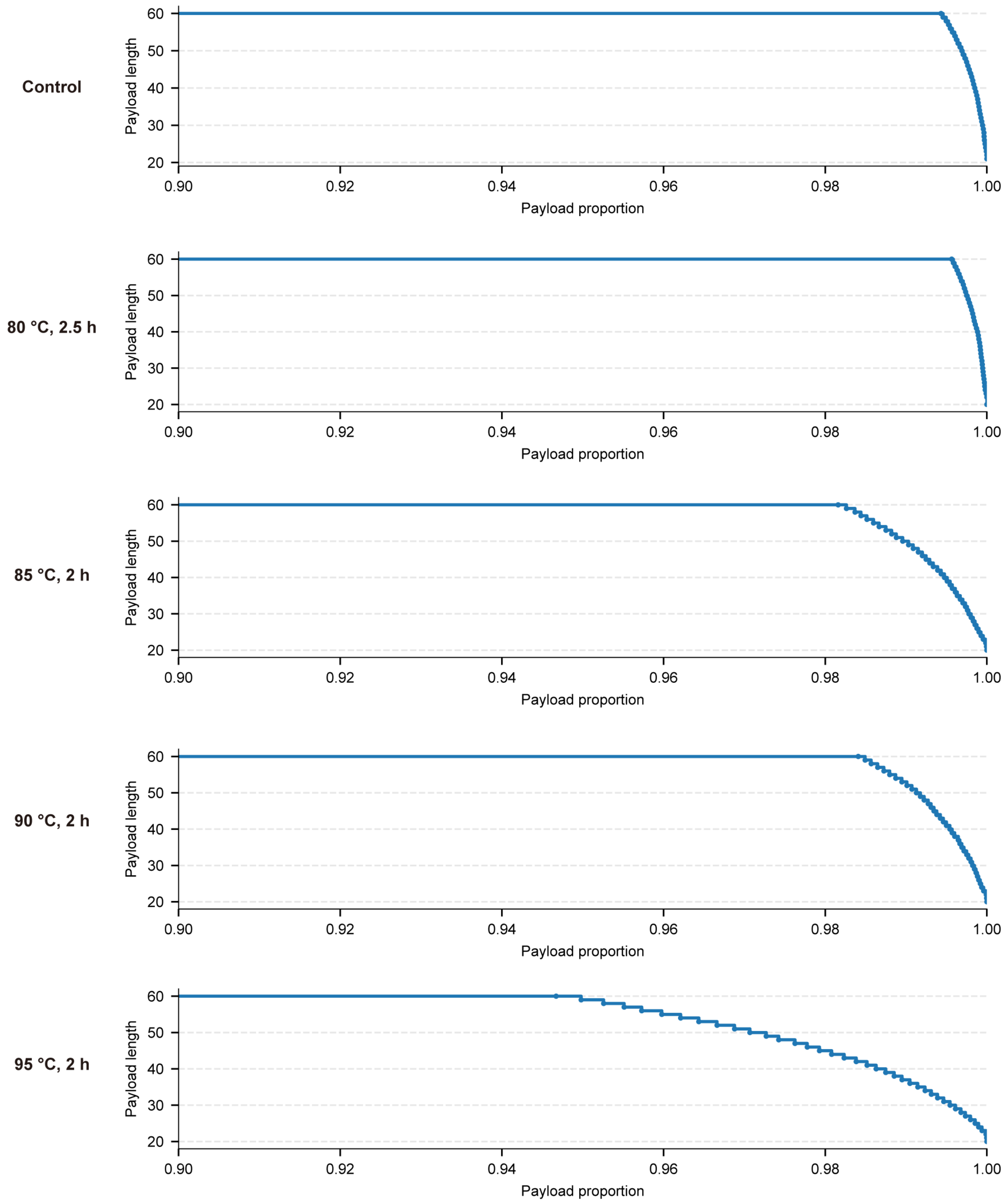

Supplementary Figure 63. Magnified view of proportion of read length under different thermal degradation conditions (only payload). Detailed view of the payload ratio range 0.9–1.0. The payload exhibited a gradual shortening trend at higher temperatures. Degradation becomes more pronounced at elevated temperatures, with partial fractures observed within the payload region. Source data are provided as a Source Data file.

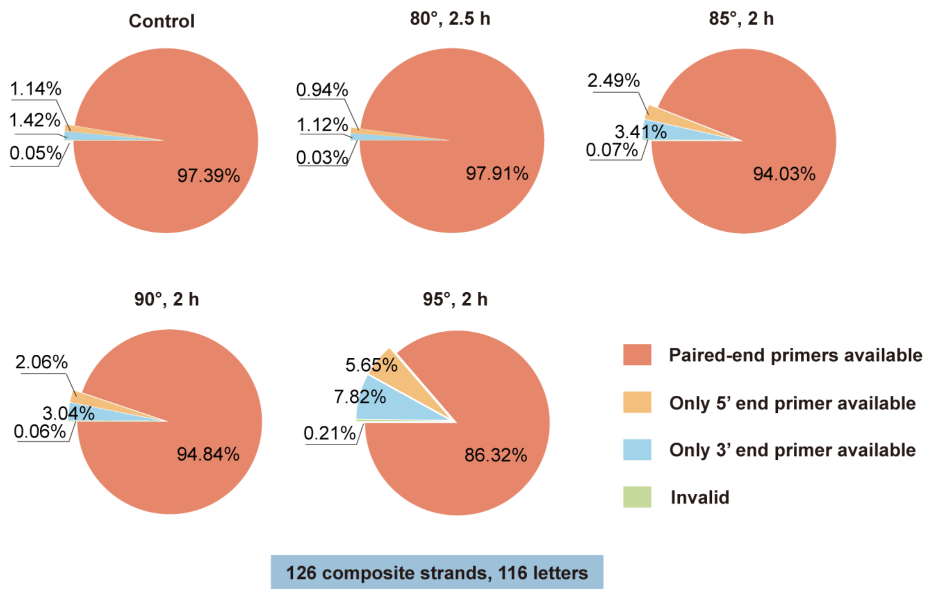

Supplementary Figure 64. Classification of sequencing reads according to primers under different thermal degradation conditions (126 composite strands, 116 nt). Proportions of reads containing paired-end primers, only 5’ primer, only 3’ primer, or invalid primers. The percentage of paired-end reads gradually decreases with increasing temperature, while single-end reads become more prevalent. At 95 °C, nearly 14% of reads exhibited fragmentation. Source data are provided as a Source Data file.

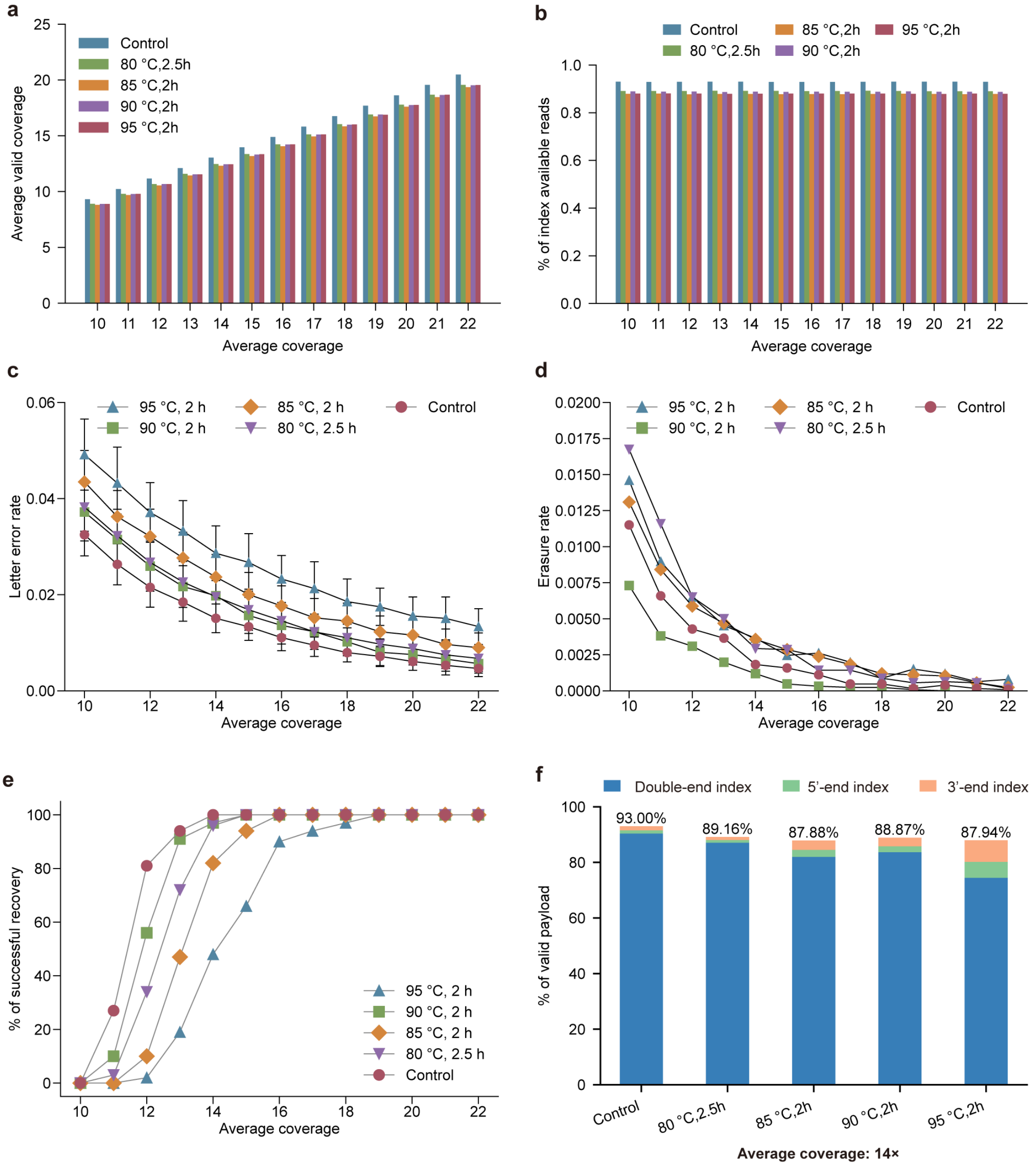

Supplementary Figure 65. Data recovery performance of degraded samples (126 composite strands, 116 nt). a, Relationship between valid base coverage and average coverage. Valid base coverage is defined as the ratio of the number of bases used for composite-letter detection to the total number of encoded letters. **b**, Index identification accuracy. The payload regions identified were used for composite-letter detection. **c**, Letter error rate as a function of average coverage for each condition. The non-heated control sample showed the lowest error rate. Error bars indicate mean ± SD (*n* = 1,000 readout trials per coverage). **d**, Erasure rate at different average coverages. **e**, Percentage of successful (error-free) recoveries across 100 independent trials using the RS(1890, 1575) coding scheme. **f**, Composition of valid payloads at 14× coverage by index identification, including double-end indices and those identified by only 5'-end or 3'-end indices. Source data are provided as a Source Data file.

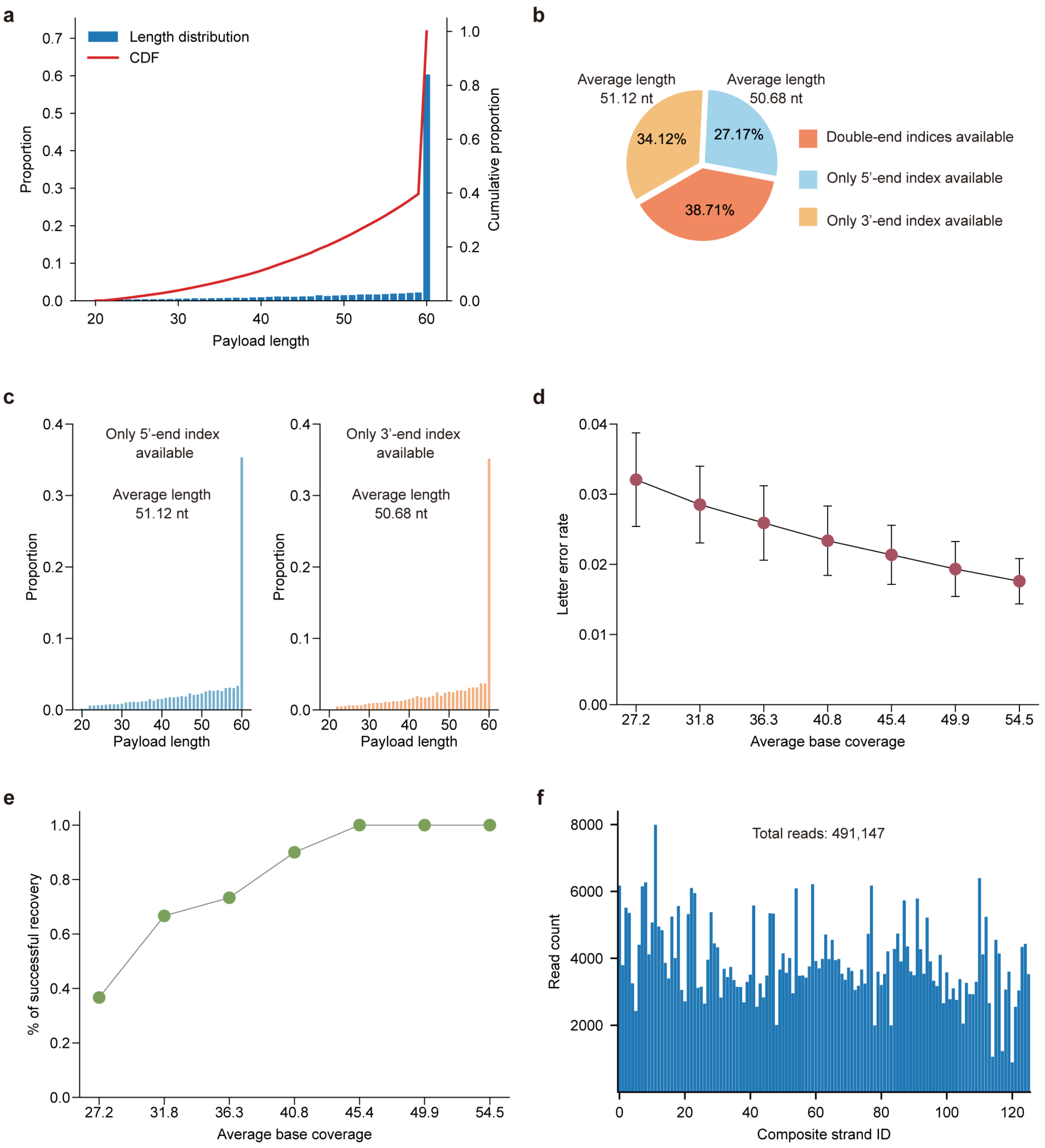

Supplementary Figure 66. Recovery performance using severely-degraded sequencing reads. a, Length distribution of payload regions identified based on double-end indices. Approximately 40% of the fragments showed degradation within the payload region. **b**, Proportion of degraded reads containing double-end indices (38.71%), only 5'-end index (27.17%), or only 3'-end index (34.12%). **c**, Length distributions of payload regions identified by single-end indices, where sequences shorter than 60 nt indicate degradation within the payload region. **d and e**, Data recovery performance based on degraded reads, showing letter error rate and successful recovery rate as functions of average base coverage (mean ± SD; n = 1,000 trials per coverage). **f**, Distribution of 491,147 degraded sequencing reads extracted from the aging experiment at 95 °C for 2 h. Source data are provided as a Source Data file.

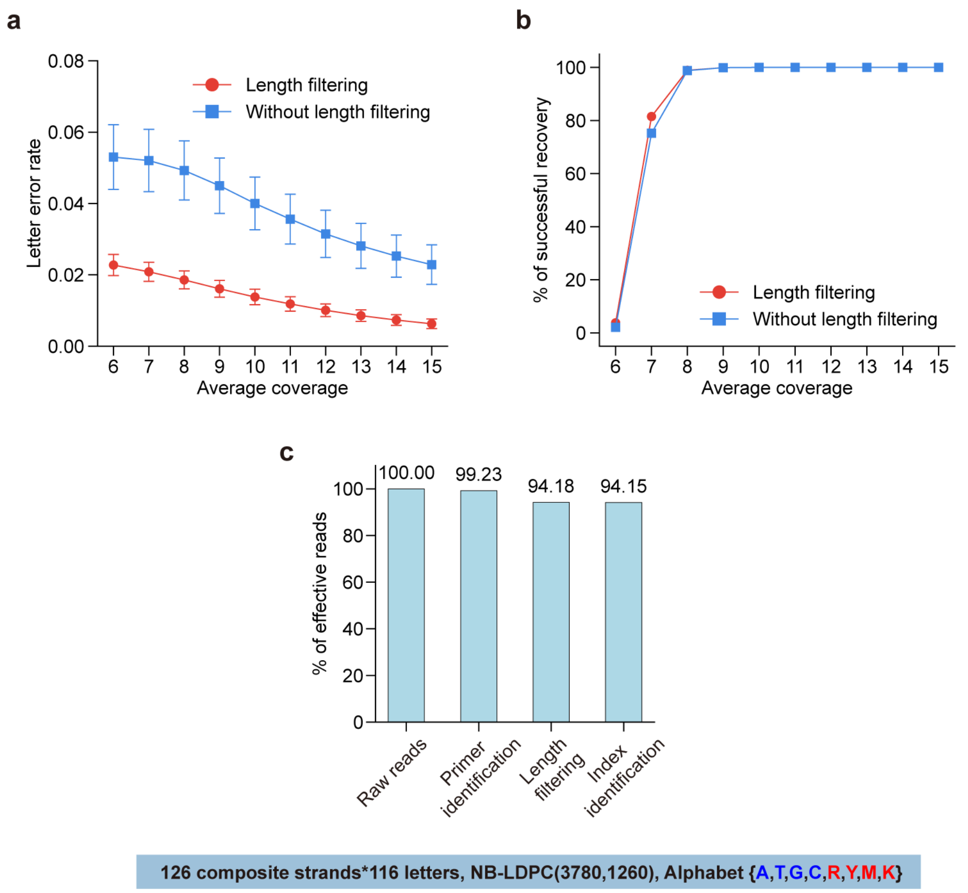

Supplementary Figure 67. Letter detection accuracy and recovery performance with length filtering. a, Letter error rates as a function of average coverage, comparing workflows with (red) and without (blue) length filtering. Length filtering effectively reduces the error rate across all coverages. Error bars indicate mean ± SD (n = 1,000 readout trials per coverage). **b,** Successful recovery rate as a function of average coverage. For each coverage, 1,000 independent evaluations were performed, and the successful recovery rate represents the proportion of tests achieving error-free recovery. Although the omission of length filtering leads to higher error rates, error-free recovery can still be achieved using NB-LDPC coding with a rate of 1/3. **c,** Proportion of valid reads at each processing step of the recovery workflow. Starting from 6,066,534 raw paired-end assembled reads (100%), 99.23% were retained after primer identification, 94.18% after length filtering, and 94.15% after index identification. Source data are provided as a Source Data file.

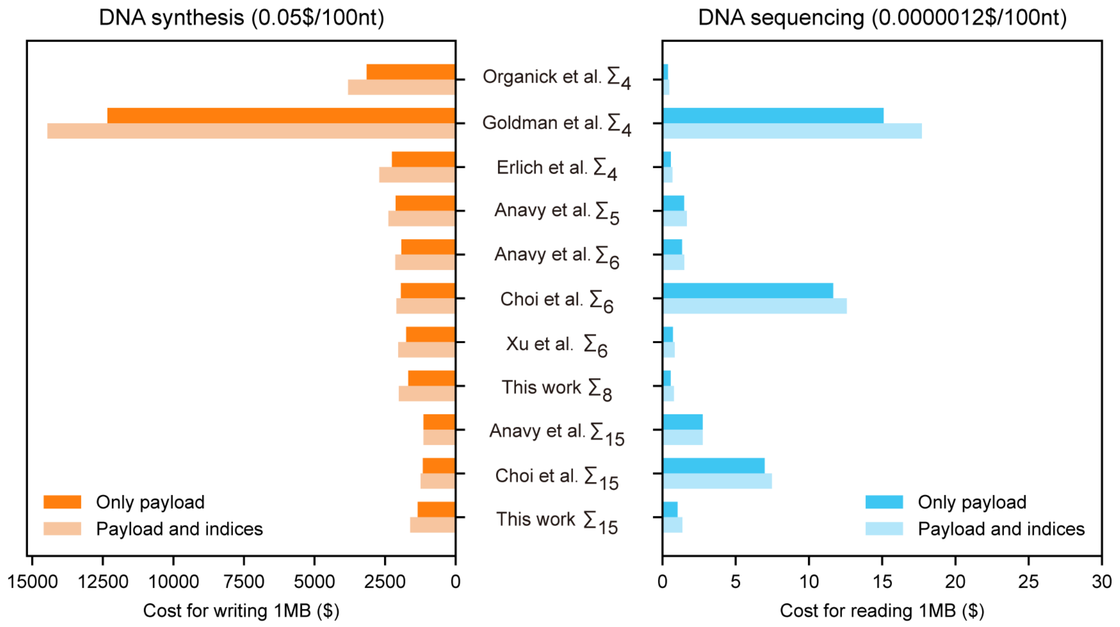

Supplementary Figure 68. Cost analysis of different DNA storage schemes. Estimated costs for writing (left) and reading (right) 1 MB of data, assuming DNA synthesis and sequencing costs of $0.05/100 nt and $0.0000012/100 nt, respectively. Two conditions are considered: Only payload part (light bars) and the region including payload and indices (dark bars). Source data are provided as a Source Data file.

Supplementary Tables

Supplementary Table 1. Comparison of published composite letter data storage schemes.

	Study	Error correction codes	Alphabet	Data volume	Total composite letters	Logical density (composite letters, bits/letter)	Logical density (including indices)	Average coverage
Exp.	Anavy et al.[1]	RS(45, 43) +Fountain (1.1)	$\sum_6$	2.12 MB	7,830,000	2.16	1.93	29×
		RS(45, 43) +Fountain (1.08)	$\sum_6$	6.42 MB	23,490,000	2.19	1.96	29×
		RS(45, 43) +Fountain (1.08)	$\sum_5$	6.42 MB	26,055,000	1.97	1.76	29×
		Binary Huffman	$\sum_{15}$	19.25 Bytes	42	3.67	3.67 (only one strand, no index)	100×
		Binary Huffman	$\sum_{20}$	22.5 Bytes	42	4.29	4.29 (only one strand, no index)	100×
	Choi et al.[2]	RS	$\sum_{15}$	854 Bytes	1,890	3.61	3.37	250×
		RS	$\sum_6$	135 KB	499,833	2.16	2.0	250×
	Xu et al.[6]	RS(45, 41) over $GF(7^3)$	$\sum_6$	6.42 MB	34,391,250	2.39	2.06	17×
	This work	NB-LDPC(3780, 1260) over $GF(2^6)$	$\sum_8$	945 Bytes*3	7,560*3	1	0.79	9×
		RS(1890, 1575) over $GF(2^{12})$	$\sum_8$	2,355 Bytes	7,560	2.5	1.97	14×
		RS(3750, 3125) over $GF(2^{12})$	$\sum_8$	187,500 Bytes*2	600,000*2	2.5	2.08 (112 letters) / 1.79 (124 letters)	16× / 19×
		RS(30000, 25000) over $GF(2^{15})$	$\sum_{15}$	234,375 Bytes*2	600,000*2	3.125	2.60 (112 letters) / 2.23 (124 letters)	35× / 33×
Sim.	Anavy et al.[1]	RS(136, 130)	$\sum_{256}$	2.12 MB	2,373,975	7.13	/	2000×
		RS(68, 65)	$\sum_{56}$	2.12 MB	3,354,480	5.05	/	500×
	Xu et al.[6]	RS(45, 41) over $GF(2^6)$	$\sum_{64}$	20.8 MB	31,495,500	5.46	/	190×
		RS(45, 41) over $GF(2^7)$	$\sum_{128}$	20.8 MB	27,776,250	6.37	/	300×
		RS(45, 41) over $GF(2^8)$	$\sum_{256}$	20.8 MB	24,273,000	7.28	/	490×
	This work	RS(32767, 27767) over $GF(2^{15})$	$\sum_{15}$	780,713 Bytes	1,966,020	3.18	/	30×

Supplementary Table 2. Composite letters and corresponding natural bases used in experimental proofs.

Composite letters	Natural bases	Reagent mix proportion	Mix proportion of four monomers (A, T, G, C)
R	A & G	1:1	(1, 0, 1, 0)
Y	C & T	1:1	(0, 1, 0, 1)
M	A & C	1:1	(1, 0, 0, 1)
K	G & T	1:1	(0, 1, 1, 0)
S	G & C	1:1	(0, 0, 1, 1)
W	A & T	1:1	(1, 1, 0, 0)
H	A & T & C	1:1:1	(1, 1, 0, 1)
B	G & T & C	1:1:1	(0, 1, 1, 1)
V	G & A & C	1:1:1	(1, 0, 1, 1)
D	G & A & T	1:1:1	(1, 1, 1, 0)
N	G & A & T& C	1:1:1:1	(1, 1, 1, 1)

Supplementary Table 3. The number of ordinary strands corresponding to four composite DNA pools in this work.

ID	Number (Exp.1)	Number (Exp.2)	Number (Exp.3)	Number (Exp.4)
1	1.07E+09	1.07E+09	1.10E+12	3.87E+08
2	2.68E+08	5.50E+11	1.41E+14	6.86E+13
3	4.29E+09	3.36E+07	3.44E+10	1.50E+17
4	2.15E+09	2.15E+09	2.81E+14	4.05E+18
5	1.37E+11	1.34E+08	1.41E+14	6.86E+13
6	4.29E+09	1.07E+09	2.81E+14	5.00E+16
7	3.44E+10	1.07E+09	1.76E+13	1.22E+19
8	1.34E+08	2.68E+08	2.25E+15	6.86E+13
9	4.29E+09	5.37E+08	5.63E+14	1.35E+18
10	3.44E+10	4.29E+09	1.76E+13	1.85E+15
11	1.10E+12	2.75E+11	1.76E+13	5.00E+16
12	1.34E+08	5.37E+08	5.50E+11	1.67E+16
13	6.71E+07	1.34E+08	2.81E+14	2.29E+13
14	5.37E+08	5.37E+08	1.41E+14	4.50E+17
15	2.75E+11	2.15E+09	4.50E+15	6.18E+14
16	2.15E+09	8.59E+09	2.20E+12	1.85E+15
17	5.37E+08	5.37E+08	3.52E+13	6.86E+13
18	2.68E+08	1.07E+09	8.59E+09	6.18E+14
19	2.15E+09	5.37E+08	1.41E+14	1.50E+17
20	6.87E+10	2.15E+09	7.04E+13	1.85E+15
21	5.37E+08	4.29E+09	7.21E+16	8.47E+11
22	6.71E+07	8.39E+06	4.40E+12	6.18E+14
23	2.10E+06	8.39E+06	1.41E+14	2.82E+11
24	4.29E+09	5.37E+08	1.37E+11	2.29E+13
25	1.34E+08	1.72E+10	3.52E+13	1.67E+16
26	8.59E+09	1.07E+09	2.25E+15	1.85E+15
27	5.37E+08	4.29E+09	3.60E+16	6.86E+13
28	2.15E+09	4.29E+09	2.75E+11	2.54E+12
29	1.34E+08	2.15E+09	1.41E+14	1.50E+17
30	4.29E+09	6.87E+10	1.10E+12	7.63E+12
31	3.36E+07	2.68E+08	1.76E+13	7.63E+12
32	1.07E+09	1.72E+10	7.04E+13	1.09E+20
33	6.71E+07	1.34E+08	4.50E+15	9.41E+10
34	4.29E+09	4.29E+09	1.10E+12	1.67E+16
35	3.44E+10	2.68E+08	1.10E+12	7.63E+12
36	2.68E+08	5.37E+08	2.75E+11	5.56E+15
37	8.59E+09	2.15E+09	7.04E+13	2.29E+13
38	4.29E+09	2.15E+09	3.52E+13	2.29E+13
39	2.15E+09	1.72E+10	2.25E+15	1.67E+16

ID	Number (Exp.1)	Number (Exp.2)	Number (Exp.3)	Number (Exp.4)
40	8.59E+09	3.36E+07	7.04E+13	1.35E+18
41	5.37E+08	8.59E+09	3.52E+13	6.18E+14
42	1.72E+10	6.71E+07	1.37E+11	2.54E+12
43	1.05E+06	2.15E+09	8.80E+12	2.29E+13
44	5.37E+08	5.37E+08	7.04E+13	6.18E+14
45	4.29E+09	1.07E+09	1.41E+14	5.56E+15
46	2.68E+08	1.07E+09	4.40E+12	6.18E+14
47	8.59E+09	1.68E+07	5.63E+14	8.47E+11
48	4.29E+09	2.15E+09	3.52E+13	1.85E+15
49	4.29E+09	2.68E+08	5.50E+11	6.86E+13
50	4.29E+09	2.68E+08	4.50E+15	6.86E+13
51	1.07E+09	2.68E+08	1.76E+13	2.29E+13
52	5.37E+08	5.37E+08	8.80E+12	6.18E+14
53	4.29E+09	5.37E+08	2.81E+14	8.47E+11
54	2.68E+08	2.68E+08	4.40E+12	2.29E+13
55	2.15E+09	1.68E+07	1.41E+14	2.06E+14
56	1.07E+09	2.15E+09	3.52E+13	6.18E+14
57	1.34E+08	1.07E+09	8.80E+12	9.41E+10
58	8.39E+06	2.15E+09	1.41E+14	7.63E+12
59	5.37E+08	2.15E+09	1.41E+14	2.29E+13
60	4.29E+09	4.29E+09	2.81E+14	6.18E+14
61	1.68E+07	4.29E+09	1.13E+15	1.85E+15
62	1.10E+12	6.87E+10	1.76E+13	2.06E+14
63	1.37E+11	5.37E+08	1.13E+15	7.63E+12
64	2.68E+08	8.59E+09	1.76E+13	1.85E+15
65	2.75E+11	2.68E+08	5.63E+14	1.85E+15
66	5.37E+08	1.07E+09	4.40E+12	1.09E+20
67	5.37E+08	1.07E+09	1.76E+13	2.06E+14
68	2.15E+09	3.36E+07	3.52E+13	2.54E+12
69	6.71E+07	2.15E+09	3.52E+13	2.29E+13
70	1.07E+09	3.36E+07	1.41E+14	7.63E+12
71	2.10E+06	1.07E+09	9.01E+15	1.35E+18
72	2.68E+08	1.72E+10	3.52E+13	1.85E+15
73	8.59E+09	3.44E+10	2.81E+14	2.06E+14
74	1.07E+09	1.72E+10	5.63E+14	8.47E+11
75	6.71E+07	1.72E+10	1.76E+13	1.85E+15
76	2.68E+08	1.72E+10	1.10E+12	1.67E+16
77	1.07E+09	1.68E+07	3.52E+13	6.86E+13
78	5.37E+08	1.07E+09	2.20E+12	1.50E+17
79	3.36E+07	1.68E+07	1.41E+14	6.18E+14
80	8.59E+09	1.72E+10	1.76E+13	2.06E+14

ID	Number (Exp.1)	Number (Exp.2)	Number (Exp.3)	Number (Exp.4)
81	4.29E+09	8.59E+09	7.04E+13	1.35E+18
82	2.15E+09	3.36E+07	1.76E+13	1.85E+15
83	4.29E+09	5.37E+08	8.80E+12	1.85E+15
84	3.44E+10	4.29E+09	2.81E+14	1.85E+15
85	4.29E+09	8.59E+09	4.40E+12	2.29E+13
86	5.24E+05	3.44E+10	2.20E+12	5.56E+15
87	1.34E+08	3.44E+10	7.04E+13	1.85E+15
88	3.44E+10	3.36E+07	7.04E+13	6.86E+13
89	3.44E+10	2.15E+09	5.63E+14	7.63E+12
90	1.34E+08	1.07E+09	8.80E+12	1.22E+19
91	5.37E+08	2.68E+08	8.80E+12	6.18E+14
92	1.07E+09	2.15E+09	1.41E+14	2.82E+11
93	5.37E+08	1.07E+09	5.63E+14	1.35E+18
94	2.68E+08	5.37E+08	5.50E+11	6.18E+14
95	1.72E+10	1.34E+08	2.81E+14	2.54E+12
96	1.34E+08	1.72E+10	1.13E+15	1.85E+15
97	6.71E+07	1.68E+07	2.81E+14	1.67E+16
98	5.37E+08	8.59E+09	7.04E+13	5.56E+15
99	1.37E+11	1.07E+09	3.52E+13	6.18E+14
100	8.59E+09	1.07E+09	3.52E+13	4.50E+17
101	2.68E+08	1.34E+08	7.04E+13	2.82E+11
102	3.36E+07	8.59E+09	2.81E+14	3.14E+10
103	2.15E+09	1.07E+09	7.04E+13	3.87E+08
104	1.34E+08	1.72E+10	4.40E+12	6.86E+13
105	3.36E+07	2.15E+09	1.76E+13	2.29E+13
106	4.29E+09	2.75E+11	4.40E+12	6.86E+13
107	5.37E+08	3.44E+10	4.40E+12	3.14E+10
108	2.15E+09	3.44E+10	3.52E+13	7.63E+12
109	5.37E+08	2.75E+11	4.40E+12	7.63E+12
110	1.68E+07	2.75E+11	1.76E+13	2.29E+13
111	5.50E+11	5.37E+08	3.52E+13	7.63E+12
112	8.59E+09	2.15E+09	1.10E+12	1.05E+10
113	1.07E+09	5.37E+08	2.20E+12	5.56E+15
114	5.37E+08	5.37E+08	1.10E+12	5.56E+15
115	2.15E+09	8.59E+09	7.04E+13	7.63E+12
116	6.71E+07	2.68E+08	7.04E+13	1.85E+15
117	3.36E+07	1.34E+08	7.04E+13	1.67E+16
118	2.15E+09	8.59E+09	3.52E+13	5.00E+16
119	8.59E+09	6.71E+07	4.40E+12	6.18E+14
120	3.44E+10	1.37E+11	2.20E+12	2.06E+14
121	6.71E+07	1.07E+09	7.04E+13	1.67E+16

ID	Number (Exp.1)	Number (Exp.2)	Number (Exp.3)	Number (Exp.4)
122	2.68E+08	1.34E+08	2.81E+14	5.00E+16
123	5.37E+08	1.07E+09	1.13E+15	6.18E+14
124	6.71E+07	1.34E+08	3.52E+13	8.47E+11
125	6.71E+07	2.15E+09	1.76E+13	1.85E+15
126	1.07E+09	8.59E+09	1.76E+13	1.85E+15

Supplementary Table 4. Coding potential and the net information density.

Scheme	Column-based DNA synthesis experiments		Array-based DNA synthesis experiments				Simulation
**Data volume (bytes)**	945	2,355	187,500		234,375		780,713
**Bit information (bits)**	7,560	18,900	1,500,000		1,875,000		6,247,575
**Payload (*M**L*)**	126*60 =7560 letters	126*60 =7560 letters	10000*60 =600,000 letters		10000*60 =600,000 letters		32767*60 =1,966,020 letters
**Alphabet**	$\sum_8$	$\sum_8$	$\sum_8$		$\sum_{15}$		$\sum_{15}$
**Coding potential (bits/letter)**	$\log_2(8) = 3$	$\log_2(8) = 3$	$\log_2(8) = 3$		$\log_2(15) = 3.9$		$\log_2(15) = 3.9$
**Coding scheme**	NB-LDPC(3780, 1260)	RS(1890, 1575)	RS(3750, 3125)		RS(30000, 25000)		RS(32767, 27767)
**Code rate**	*R*=1/3	*R*=5/6	*R*=5/6		*R*=5/6		*R*=17/20
**Logical density (payload)**	1 bit/letter	2.5 bits/letter	2.5 bits/letter		3.125 bits/letter		3.18 bits/letter
**Synthesized nucleotides (including primers/indices)**	126*116 =14,616	126*116 =14,616	10000*124 =1,240,000	10000*112 =1,120,000	10000*124 =1,240,000	10000*112 =1,120,000	/
**Logical density (payload+index)**	0.79 bit/letter	1.97 bits/letter	1.79 bits/letter	2.08 bits/letter	2.23 bits/letter	2.60 bits/letter	/
**Net information density (synthesized nucleotides) (bits/letter)**	0.52 bits/letter	1.29 bits/letter	1.21 bits/letter	1.34 bits/letter	1.51 bits/letter	1.67 bits/letter	/

Supplementary Table 5. Primers used in this work.

Primers	Sequences
Forward	TCACCATCCACTCTAAACAC
Reverse	ATGAGTGGAGGTGTAAAGTG

Supplementary Table 6. Unique index used in this work.

ID	Index sequence	ID	Index sequence
1	GAATCTATCAAAGAAT	64	AAACGGACGAATGAAG
2	TAAACGGTGGAACAAA	65	GAAGTCAGTAATAAAC
3	CAATACGATGAATAAT	66	TAACTAGGAGATTAAG
4	AGAATCTTGTAAGGAA	67	CAAGGTGCCGATCAAC
5	GGATGGTATTAAAGAT	68	AGACCTTGATATAGAG
6	TGAAGTCAACAATGAA	69	GGAGAATCCTATGGAC
7	CGATTACTCCAACGAT	70	TGACACCCGCATCGAG
8	ATAAACGCGAGAGTAA	71	CGAGCGCGTCATTGAC
9	GTATCGGGTAGAATAT	72	ATACGTGAAAGTATAG
10	TTAACTAGAGGATTAA	73	GTAGTAGTCAGTGTAC
11	CTATAAACCGGACTAT	74	TTACTCATGGGTCTAG
12	ACAATACGATGAACAA	75	CTAGGGAATGGTTTAC
13	GCATGTCCCTGAGCAT	76	ACACCGCTGTGTGCAG
14	TCAAGGTCGCGACCAA	77	GCAGACCATTGTACAC
15	CCATTCTGTCGATCAT	78	TCACAATAACGTTCAG
16	AAGTCACAGATAGAGA	79	CCAGCTTTCCGTCCAC
17	GAGAATCTTATAAAGT	80	AAGGTGCCAATTAAGG
18	TAGTAGTTAGTATAGA	81	GAGCGCCGCATTGAGC
19	CAGACCTACGTACAGT	82	TAGGGATGGGTTCAGG
20	AGGTGCGTATTAAGGA	83	CAGCTTTCTGTTTAGC
21	GGGATGGACTTAGGGT	84	AGGGATGGGTTTGGGG
22	TGGTTTAAGCTACGGA	85	GGGCCAGCTTTTAGGC
23	CGGAGAATTCTATGGT	86	TGGGCCACACTTTGGG
24	ATGTCCTCAACAATGA	87	CGGCAGAGCCTTCGGC
25	GTGAAGTGCACAGTGT	88	ATGGTTTAGACTGTGG
26	TTGTATCGGGCACTGA	89	GTGCGATTTACTATGC
27	CTGACACCTGCATTGT	90	TTGGGCCTAGCTTTGG
28	ACGTGAAGGTCAGCGA	91	CTGCTGCACGCTCTGC
29	GCGATTACTTCAACGT	92	ACGGAGATATCTACGG
30	TCGTTGGCACCATCGA	93	GCGCCCAACTCTGCGC
31	CCGAGCGGCCCACCGT	94	TCGGCAGAGCCTCCGG
32	AATACGTGGAAGGATA	95	CCGCATGTTCCTTCGC
33	GATTACTCTAAGAATT	96	AATCTATTAAACAATG
34	TATAAACCAGAGTATA	97	GATGGTTACAACGATC
35	CATTCTCGCGAGCATT	98	TATCGGCAGGACCATG
36	AGTAGTACATAGAGTA	99	CATGTCCTTGACTATC
37	GGTTTAAGCTAGGGTT	100	AGTCACAAGTACGGTG
38	TGTATCGGGCAGCGTA	101	GGTGCGATTTACAGTC
39	CGTTGGGCTCAGTGTT	102	TGTCCTGTACACTGTG
40	ATTACTCTAAGGATTA	103	CGTGAAGACCACCGTC

ID	Index sequence	ID	Index sequence
41	GTTTAACACAGGGTTT	104	ATTCTCCGGAGCGTTG
42	TTTAACTAGGGGCTTA	105	GTTGGGCCTAGCATTC
43	CTTTCGTTTGGGTTTT	106	TTTCGTTCAGGCTTTG
44	ACTAGGGAGTGGGCTA	107	CTTGTATGCGGCCTTC
45	GCTTTCGTTTGGACTT	108	ACTCAAGCATGCACTG
46	TCTATAATACGGTCTA	109	GCTGCTGGCTGCGCTC
47	CCTTGTAACCGGCCTT	110	TCTCCGAGGCGCCCTG
48	AACTAGGGAATGAACA	111	CCTGACACTCGCTCTC
49	GACACCGCCATGGACT	112	AACGGAGTGATCGACG
50	TACTCAACGGTGCACA	113	GACCTTGATATCAACC
51	CACAATAGTGTGTACT	114	TACGTGAAAGTCTACG
52	AGCTTTCCGTTGGGCA	115	CACCGCATCGTCCACC
53	GGCAGACGTTTGAGCT	116	AGCGCCCAATTCAGCG
54	TGCTGCTGACTGTGCA	117	GGCCAGCTCTTCGGCC
55	CGCATGTCCCTGCGCT	118	TGCGATTTGCTCCGCG
56	ATCTATATGACGGTCA	119	CGCCCATATCTCTGCC
57	GTCACAAATACGATCT	120	ATCGGCAGAACCATCG
58	TTCTCCGAAGCGTTCA	121	GTCCTGACCACCGTCC
59	CTCAAGGTCGCGCTCT	122	TTCGTTGCGGCCCTCG
60	ACCTTGTAATCGACCA	123	CTCCGAGGTGCCTTCC
61	GCCAGCTTCTCGGCCT	124	ACCGCATCGTCCGCCG
62	TCCTGACTGCCGCCCA	125	GCCCATTGTTCCACCC
63	CCCATTCATCCGTCCT	126	TCCGAGCGACCCTCCG

Supplementary Table 7. Letter detection error rates under different detection methods with an eight-letter alphabet {A, T, G, C, R, Y, M, K} and RS(1890, 1575).

Method	Cov.	A(%)	T(%)	G(%)	C(%)	R(%)	Y(%)	M(%)	K(%)	Total
SP	10×	0.129	0.089	0.138	0.093	0.294	0.454	0.293	0.264	1.754
	11×	0.123	0.082	0.126	0.087	0.243	0.390	0.246	0.223	1.519
	12×	0.117	0.075	0.116	0.081	0.204	0.341	0.206	0.187	1.327
	13×	0.113	0.070	0.108	0.076	0.172	0.298	0.175	0.159	1.170
	14×	0.110	0.066	0.100	0.072	0.145	0.262	0.150	0.137	1.042
	15×	0.105	0.061	0.093	0.068	0.122	0.229	0.131	0.117	0.926
	16×	0.103	0.058	0.089	0.065	0.104	0.200	0.112	0.104	0.834
	17×	0.101	0.055	0.085	0.064	0.090	0.176	0.097	0.091	0.758
	18×	0.098	0.052	0.081	0.061	0.077	0.156	0.085	0.082	0.691
	19×	0.096	0.050	0.079	0.060	0.067	0.138	0.074	0.073	0.636
	20×	0.096	0.049	0.078	0.059	0.060	0.123	0.066	0.066	0.597
	21×	0.095	0.047	0.076	0.059	0.054	0.109	0.058	0.060	0.558
KL	10×	0.215	0.153	0.276	0.174	0.235	0.339	0.227	0.207	1.825
	11×	0.222	0.160	0.289	0.183	0.182	0.268	0.177	0.164	1.644
	12×	0.225	0.164	0.296	0.186	0.142	0.213	0.139	0.130	1.493
	13×	0.225	0.165	0.298	0.186	0.113	0.170	0.111	0.104	1.372
	14×	0.219	0.163	0.295	0.185	0.091	0.139	0.090	0.086	1.268
	15×	0.213	0.159	0.287	0.183	0.073	0.114	0.074	0.072	1.175
	16×	0.206	0.154	0.279	0.178	0.060	0.094	0.062	0.063	1.096
	17×	0.197	0.148	0.267	0.172	0.051	0.080	0.052	0.056	1.022
	18×	0.188	0.141	0.255	0.166	0.045	0.068	0.046	0.051	0.959
	19×	0.183	0.136	0.243	0.161	0.039	0.059	0.040	0.046	0.907
	20×	0.178	0.131	0.232	0.155	0.035	0.054	0.036	0.044	0.864
	21×	0.173	0.126	0.220	0.149	0.032	0.047	0.033	0.041	0.820
MAP	10×	0.126	0.088	0.161	0.108	0.250	0.377	0.243	0.219	1.572
	11×	0.116	0.082	0.149	0.102	0.200	0.312	0.196	0.179	1.336
	12×	0.109	0.076	0.139	0.096	0.162	0.261	0.161	0.146	1.150
	13×	0.102	0.071	0.127	0.090	0.135	0.222	0.135	0.121	1.002
	14×	0.098	0.066	0.117	0.086	0.113	0.193	0.116	0.104	0.892
	15×	0.094	0.062	0.108	0.082	0.096	0.169	0.102	0.090	0.801
	16×	0.092	0.060	0.102	0.078	0.083	0.148	0.089	0.082	0.735
	17×	0.089	0.058	0.097	0.076	0.074	0.132	0.081	0.075	0.680
	18×	0.086	0.053	0.090	0.072	0.067	0.119	0.074	0.070	0.631
	19×	0.084	0.051	0.086	0.071	0.061	0.108	0.069	0.065	0.594
	20×	0.082	0.049	0.083	0.069	0.058	0.101	0.066	0.063	0.572
	21×	0.082	0.048	0.081	0.068	0.055	0.092	0.063	0.061	0.550

Supplementary Table 8. Letter detection error rates under different detection methods with a 15-letter alphabet (10,000 composite strands, 124 letters).

Method	Cov.	A(%)	T(%)	G(%)	C(%)	R(%)	Y(%)	M(%)	K(%)	S(%)	W(%)	H(%)	B(%)	V(%)	D(%)	N(%)	Total
	26×	0.037	0.044	0.073	0.032	0.245	0.129	0.109	0.225	0.240	0.151	0.272	0.375	0.184	0.381	0.809	3.305
	30×	0.033	0.041	0.063	0.029	0.203	0.098	0.088	0.171	0.198	0.114	0.198	0.285	0.156	0.291	0.633	2.602
	34×	0.030	0.040	0.056	0.027	0.167	0.078	0.072	0.135	0.160	0.090	0.148	0.220	0.140	0.228	0.502	2.090
**SP**	38×	0.028	0.038	0.051	0.025	0.141	0.066	0.061	0.110	0.132	0.074	0.113	0.173	0.127	0.181	0.402	1.722
	42×	0.026	0.037	0.048	0.024	0.126	0.059	0.054	0.094	0.116	0.064	0.088	0.137	0.115	0.146	0.324	1.457
	46×	0.024	0.037	0.045	0.023	0.119	0.054	0.051	0.084	0.106	0.058	0.070	0.110	0.103	0.119	0.263	1.265
	50×	0.023	0.036	0.042	0.022	0.114	0.051	0.049	0.077	0.101	0.054	0.056	0.089	0.090	0.098	0.215	1.119
	26×	0.174	0.166	0.415	0.146	0.461	0.155	0.196	0.324	0.447	0.228	0.111	0.137	0.192	0.185	0.266	3.431
	30×	0.145	0.138	0.325	0.120	0.493	0.159	0.208	0.336	0.479	0.236	0.087	0.108	0.197	0.164	0.155	3.349
	34×	0.128	0.122	0.263	0.104	0.490	0.155	0.205	0.330	0.477	0.230	0.079	0.097	0.211	0.160	0.091	3.141
**KL**	38×	0.119	0.112	0.225	0.095	0.453	0.144	0.189	0.306	0.441	0.212	0.077	0.093	0.226	0.162	0.055	2.909
	42×	0.113	0.108	0.202	0.089	0.396	0.129	0.164	0.270	0.384	0.187	0.077	0.092	0.237	0.164	0.034	2.647
	46×	0.108	0.104	0.187	0.085	0.335	0.114	0.139	0.234	0.323	0.161	0.076	0.091	0.238	0.164	0.021	2.379
	50×	0.105	0.101	0.174	0.082	0.284	0.101	0.118	0.203	0.269	0.141	0.073	0.087	0.230	0.158	0.014	2.138
	26×	0.040	0.046	0.067	0.035	0.108	0.102	0.050	0.182	0.104	0.115	0.197	0.251	0.145	0.264	0.448	2.152
	30×	0.036	0.043	0.058	0.031	0.079	0.074	0.036	0.132	0.075	0.082	0.138	0.182	0.097	0.192	0.356	1.611
	34×	0.033	0.040	0.051	0.029	0.064	0.060	0.029	0.103	0.059	0.064	0.097	0.131	0.065	0.140	0.278	1.243
**MAP**	38×	0.030	0.039	0.046	0.027	0.054	0.051	0.025	0.086	0.050	0.054	0.069	0.097	0.045	0.105	0.210	0.990
	42×	0.028	0.037	0.043	0.026	0.048	0.046	0.023	0.075	0.045	0.047	0.052	0.074	0.033	0.082	0.156	0.815
	46×	0.027	0.037	0.040	0.025	0.044	0.044	0.021	0.068	0.041	0.043	0.040	0.058	0.025	0.066	0.116	0.694
	50×	0.026	0.036	0.038	0.024	0.041	0.042	0.020	0.063	0.039	0.040	0.033	0.047	0.021	0.055	0.087	0.611

Supplementary Table 9. Length filtering of the different experiments.

		Exp.1	Exp.2	Exp.3	Exp.4	Exp.5	Exp.6
**Composite strands**		126	126	10,000	10,000	10,000	10,000
**Alphabet**		8	8	8	8	15	15
**Length (Primer+index)**		28 nt	28 nt	32 nt	26 nt	32 nt	26 nt
**Error rate (Forward primer+index)**	Total	0.0059	0.0062	0.0102	0.0030	0.0087	0.0134
	Ins.	0.0001	0.0001	0.0004	0.0001	0.0004	0.0001
	Del.	0.0053	0.0056	0.0085	0.0027	0.0071	0.0129
	Sub.	0.0005	0.0005	0.0013	0.0002	0.0012	0.0004
**Error rate (Reverse primer+index)**	Total	0.0020	0.0020	0.0040	0.0009	0.0036	0.0036
	Ins.	0.0001	0.0001	0.0003	0.0001	0.0003	0.0001
	Del.	0.0013	0.0013	0.0025	0.0005	0.0021	0.0031
	Sub.	0.0006	0.0006	0.0012	0.0003	0.0012	0.0004
**Length (Index+payload)**		76	76	84	72	84	72
**Length filtering (Valid reads)**		94.9%	94.9%	85.9%	87.6%	86.5%	86.7%
**Length filtering (Invalid reads)**		5.1%	5.1%	14.1%	12.4%	13.5%	13.3%

Supplementary Table 10. Comparative cost analysis aligned with prior studies.

Alphabet	Study	Cov.	Logical density (bits/letter) (excluding primers)	Normalized cost C_{syn}:C_{seq} (500:1)	Normalized cost C_{syn}:C_{seq} (1000:1)
$\sum_4$	**Organick et al.[15]**	5×	1.10	1.00	1.00
	**Goldman et al.[16]**	51×	0.29	4.14	3.97
	**Erlich et al.[3]**	10.5×	1.55	0.72	0.71
$\sum_5$	**Anavy et al.[1]**	29×	1.76	0.65	0.64
$\sum_6$	**Anavy et al.[1]**	29×	1.96	0.59	0.57
	**Choi et al.[2]**	250×	2.0	0.82	0.68
	**Xu et al.[6]**	17×	2.06	0.55	0.54
$\sum_8$	**This work**	16×	2.08	0.54	0.53
$\sum_{15}$	**Anavy et al.[1]**	100×	3.67	0.36	0.33
	**Choi et al.[2]**	250×	3.37	0.48	0.41
	**This work**	35×	2.60	0.45	0.44
$\sum_{20}$	**Anavy et al.[1]**	100×	4.29	0.30	0.28

Supplementary Table 11. Initial vectors and normalized probability vectors corresponding to the 11 composite letters.

Composite letters	Corresponding vector (A, T, G, C)	Probability vector $\sigma(\sigma_{\mathrm{A}}, \sigma_{\mathrm{T}}, \sigma_{\mathrm{G}}, \sigma_{\mathrm{C}})$
R	(1, 0, 1, 0)	$\left(\frac{1+\epsilon}{2+4\epsilon}, \frac{\epsilon}{2+4\epsilon}, \frac{1+\epsilon}{2+4\epsilon}, \frac{\epsilon}{2+4\epsilon}\right)$
Y	(0, 1, 0, 1)	$\left(\frac{\epsilon}{2+4\epsilon}, \frac{1+\epsilon}{2+4\epsilon}, \frac{\epsilon}{2+4\epsilon}, \frac{1+\epsilon}{2+4\epsilon}\right)$
M	(1, 0, 0, 1)	$\left(\frac{1+\epsilon}{2+4\epsilon}, \frac{\epsilon}{2+4\epsilon}, \frac{\epsilon}{2+4\epsilon}, \frac{1+\epsilon}{2+4\epsilon}\right)$
K	(0, 1, 1, 0)	$\left(\frac{\epsilon}{2+4\epsilon}, \frac{1+\epsilon}{2+4\epsilon}, \frac{1+\epsilon}{2+4\epsilon}, \frac{\epsilon}{2+4\epsilon}\right)$
S	(0, 0, 1, 1)	$\left(\frac{\epsilon}{2+4\epsilon}, \frac{\epsilon}{2+4\epsilon}, \frac{1+\epsilon}{2+4\epsilon}, \frac{1+\epsilon}{2+4\epsilon}\right)$
W	(1, 1, 0, 0)	$\left(\frac{1+\epsilon}{2+4\epsilon}, \frac{1+\epsilon}{2+4\epsilon}, \frac{\epsilon}{2+4\epsilon}, \frac{\epsilon}{2+4\epsilon}\right)$
H	(1, 1, 0, 1)	$\left(\frac{1+\epsilon}{3+4\epsilon}, \frac{1+\epsilon}{3+4\epsilon}, \frac{\epsilon}{3+4\epsilon}, \frac{1+\epsilon}{3+4\epsilon}\right)$
B	(0, 1, 1, 1)	$\left(\frac{\epsilon}{3+4\epsilon}, \frac{1+\epsilon}{3+4\epsilon}, \frac{1+\epsilon}{3+4\epsilon}, \frac{1+\epsilon}{3+4\epsilon}\right)$
V	(1, 0, 1, 1)	$\left(\frac{1+\epsilon}{3+4\epsilon}, \frac{\epsilon}{3+4\epsilon}, \frac{1+\epsilon}{3+4\epsilon}, \frac{1+\epsilon}{3+4\epsilon}\right)$
D	(1, 1, 1, 0)	$\left(\frac{1+\epsilon}{3+4\epsilon}, \frac{1+\epsilon}{3+4\epsilon}, \frac{1+\epsilon}{3+4\epsilon}, \frac{\epsilon}{3+4\epsilon}\right)$
N	(1, 1, 1, 1)	$\left(\frac{1}{4}, \frac{1}{4}, \frac{1}{4}, \frac{1}{4}\right)$

Supplementary Table 12. PCR reaction components used in this work.

**Components**	**Per 25 μL reaction**
Oligo pool (0.1 ng/μL)	1 μL
NF water	7.75 μL
Forward primer (10 μM)	1.5 μL
Reverse primer (10 μM)	1.5 μL
DMSO	0.75 Ml
Phusion Hot Start Flex 2X Master Mix	12.5 μL

Supplementary Table 13. PCR reaction conditions.

Cycling step	Temperatures	Duration	Number of cycles
Initialization Denaturation	98 ℃	1 min	1
Denaturation	98 ℃	10 sec	10
Annealing	60 ℃	30 sec	
Extension	72 ℃	30 sec	
Final Extension	72 ℃	1 min	1
Preservation	4 ℃	Hold on	

Supplementary Table 14. Negative binomial distribution parameters for each fitted sequencing coverage.

Theoretical sequencing coverage M	Fitted sequencing coverage D	Shape parameter r	Success probability p
10	9.3254	6.8505	0.4235
15	14.0635	10.3779	0.4246
20	18.9286	6.2064	0.2469
25	23.2381	8.4777	0.2673
30	28.3810	7.1008	0.2001
35	32.6190	7.7211	0.1914
40	37.9762	6.7972	0.1518
45	42.1111	7.1940	0.1459
50	47.8016	6.5719	0.1209
55	51.3651	6.6219	0.1142
60	57.2302	6.4745	0.1016
65	60.6111	6.7095	0.0997
70	66.7619	6.4586	0.0882
75	69.8095	7.2540	0.0941
80	75.8810	6.6494	0.0806

Supplementary References

1. Anavy, L., Vaknin, I., Atar, O., Amit, R. & Yakhini, Z. Data storage in DNA with fewer synthesis cycles using composite DNA letters. *Nat. Biotechnol.* **37**, 1229–1236 (2019).
2. Choi, Y. et al. High information capacity DNA-based data storage with augmented encoding characters using degenerate bases. *Sci. Rep.* **9**, 6582 (2019).
3. Erlich, Y. & Zielinski, D. DNA Fountain enables a robust and efficient storage architecture. *Science* **355**, 950–954 (2017).
4. Chen, W. et al. An artificial chromosome for data storage. *Natl Sci. Rev*. **8**, nwab028 (2021).
5. Ping, Z. et al. Towards practical and robust DNA-based data archiving using the yin–yang codec system. *Nat. Comput. Sci.* **2**, 234–242 (2022).
6. Xu, Y., Ding, L., Wu, S. & Ruan, J. Overcoming the high error rate of composite DNA letters-based digital storage through soft-decision decoding. *Adv. Sci.* **11**, 2402951 (2024).
7. Liu, Z. et al. Family of mutually uncorrelated codes for DNA storage address design. *IEEE Trans. Nanobiosci.* **24**, 295–304 (2025).
8. Davey, M. C. & MacKay, D. Low-density parity check codes over GF(q). *IEEE Commun. Lett.* **2**, 165–167 (1998).
9. Chen, W., Liang, C., Guo, T. & Ding, Y. Encoder implementation with FPGA for non-binary LDPC codes. In *2012 18th Asia-Pacific Conference on Communications (APCC)* 980–984 (IEEE, 2012); https://doi.org/10.1109/APCC.2012.6388230
10. Ge, Q. et al. Pragmatic soft-decision data readout of encoded large DNA. *Brief. Bioinform.* 26, bbaf102 (2025).
11. Rougemont, J. et al. Probabilistic base calling of Solexa sequencing data. *BMC Bioinformatics* **9**, 431 (2008).
12. Ungerboeck, G. Channel coding with multilevel/phase signals. *IEEE Trans. Inf. Theory* **28**, 55–67 (1982).
13. Wachsmann, U., Fischer, RFH. & Huber, JB. Multilevel codes: theoretical concepts and practical design rules. *IEEE Trans. Inf. Theory* **45**, 1361–1391 (1999).
14. Wetterstrand KA. DNA sequencing costs: data from the NHGRI genome sequencing program (GSP). Available at: www.genome.gov/sequencingcostsdata (2019).

15. Organick, L. et al. Random access in large-scale DNA data storage. *Nat. Biotechnol.* **36**, 242–248 (2018).
16. Goldman, N. et al. Towards practical, high-capacity, low-maintenance information storage in synthesized DNA. *Nature* **494**, 77–80 (2013).
