# Supplementary material for: DNA diamond formulates a decomposable composite letter constellation model for DNA data storage
Source: Nat Commun. 2026 Jan 31;17:1704. doi: 10.1038/s41467-026-68861-y (PMC12909984; doi:10.1038/s41467-026-68861-y)
Supplement: Supplementary file 2 — Description of Additional Supplementary Files [file 41467_2026_68861_MOESM2_ESM.pdf]

Description of Additional Supplementary Files:

Supplementary Data 1: Encoded DNA sequences for eight encoded composite-letter pools.
