# Supplementary material for: DNA diamond formulates a decomposable composite letter constellation model for DNA data storage
Source: Nat Commun. 2026 Jan 31;17:1704. doi: 10.1038/s41467-026-68861-y (PMC12909984; doi:10.1038/s41467-026-68861-y)
Supplement: Supplementary file 4 — Reporting Summary [file 41467_2026_68861_MOESM4_ESM.pdf]

nature portfolio

Corresponding author(s): Weigang Chen
Yingjin Yuan

Last updated by author(s): Dec 16, 2025

Reporting Summary

Nature Portfolio wishes to improve the reproducibility of the work that we publish. This form provides structure for consistency and transparency in reporting. For further information on Nature Portfolio policies, see our Editorial Policies and the Editorial Policy Checklist.

Statistics

For all statistical analyses, confirm that the following items are present in the figure legend, table legend, main text, or Methods section.

n/a	Confirmed	
☐	☒	The exact sample size (n) for each experimental group/condition, given as a discrete number and unit of measurement
☐	☒	A statement on whether measurements were taken from distinct samples or whether the same sample was measured repeatedly
☒	☐	The statistical test(s) used AND whether they are one- or two-sided<br>*Only common tests should be described solely by name; describe more complex techniques in the Methods section.*
☒	☐	A description of all covariates tested
☒	☐	A description of any assumptions or corrections, such as tests of normality and adjustment for multiple comparisons
☐	☒	A full description of the statistical parameters including central tendency (e.g. means) or other basic estimates (e.g. regression coefficient) AND variation (e.g. standard deviation) or associated estimates of uncertainty (e.g. confidence intervals)
☒	☐	For null hypothesis testing, the test statistic (e.g. F, t, r) with confidence intervals, effect sizes, degrees of freedom and P value noted<br>*Give P values as exact values whenever suitable.*
☒	☐	For Bayesian analysis, information on the choice of priors and Markov chain Monte Carlo settings
☒	☐	For hierarchical and complex designs, identification of the appropriate level for tests and full reporting of outcomes
☒	☐	Estimates of effect sizes (e.g. Cohen's d, Pearson's r), indicating how they were calculated

Our web collection on statistics for biologists contains articles on many of the points above.

Software and code

Policy information about availability of computer code

Data collection	DNA libraries were sequenced by Novogene on an Illumina NovaSeq X Plus platform, generating 150-nt paired-end (PE150) raw reads in FASTQ format. The deposited datasets correspond to the raw instrument output and were not subjected to additional quality control or filtering prior to downstream analyses.
Data analysis	Data readout and composite-letter detection were performed using custom C/C++ code (GitHub: https://github.com/TJU-QiGe/Two-stage-composite-letter-detection-method-using-set-partitioning, v1.0.0); The specific version of the code associated with this publication is archived in Zenodo and is accessible via https://doi.org/10.5281/zenodo.17905993; The pipeline calls third-party tools including seqtk v1.5 for FASTQ preprocessing, edlib v1.2.7 for sequence alignment, and a Reed–Solomon decoder based on reference implementations by Morelos-Zaragoza, R. Shell scripts were used to orchestrate the workflow on Linux. Figures were generated using Python 3.11.5.

For manuscripts utilizing custom algorithms or software that are central to the research but not yet described in published literature, software must be made available to editors and reviewers. We strongly encourage code deposition in a community repository (e.g. GitHub). See the Nature Portfolio guidelines for submitting code & software for further information.

Data

Policy information about availability of data

All manuscripts must include a data availability statement. This statement should provide the following information, where applicable:

- Accession codes, unique identifiers, or web links for publicly available datasets
- A description of any restrictions on data availability
- For clinical datasets or third party data, please ensure that the statement adheres to our policy

Encoded composite-letter sequences are available via Zenodo at https://doi.org/10.5281/zenodo.17350307. The Illumina sequencing data (raw FASTQ) from the array-based synthesis pools are deposited in the NCBI Sequence Read Archive (SRA) under accession PRJNA1345374 and are also archived on Zenodo (doi:10.5281/zenodo.17350307). Sequencing data (raw FASTQ) from the column-based synthesis experiments are deposited in SRA under accession PRJNA1258704 and are also archived on Zenodo (doi:10.5281/zenodo.15337157). Source data are provided with this paper. No restrictions on data availability.

Research involving human participants, their data, or biological material

Policy information about studies with human participants or human data. See also policy information about sex, gender (identity/presentation), and sexual orientation and race, ethnicity and racism.

Reporting on sex and gender	N/A
Reporting on race, ethnicity, or other socially relevant groupings	N/A
Population characteristics	N/A
Recruitment	N/A
Ethics oversight	N/A

Note that full information on the approval of the study protocol must also be provided in the manuscript.

Field-specific reporting

Please select the one below that is the best fit for your research. If you are not sure, read the appropriate sections before making your selection.

[x] Life sciences [ ] Behavioural & social sciences [ ] Ecological, evolutionary & environmental sciences

For a reference copy of the document with all sections, see nature.com/documents/nr-reporting-summary-flat.pdf

Life sciences study design

All studies must disclose on these points even when the disclosure is negative.

Sample size	No statistical method was used to predetermine sample size. Eight composite-letter pools were synthesized: four column-based pools (126 strands per pool; 116 nt) and four array-based pools (10,000 strands per pool; 112/124 nt), chosen to evaluate decoding performance across pool types and composite-letter constellations. For per-pool performance summaries, the effective sample sizes for each analysis are reported in the corresponding figure legends.
Data exclusions	No data were excluded from the analyses. All sequencing reads were processed from the raw data after paired-end read assembly using predefined, objective criteria (e.g., index validation and length filtering) implemented in the pipeline.
Replication	The study evaluates decoding and recovery performance using repeated, independent readout trials. For performance statistics, recovery experiments were repeated up to 1,000 independent decoding trials per coverage level to assess robustness and reproducibility.
Randomization	Randomization was not applicable because no experimental units were allocated to different intervention groups. The recovery pipeline is deterministic, and outcomes are determined by sequencing error rates and coverage rather than processing order or batch assignment.
Blinding	Blinding was not applicable because the data analysis and recovery pipeline is fully automated and deterministic, without subjective decision-making or manual outcome assessment.

Reporting for specific materials, systems and methods

We require information from authors about some types of materials, experimental systems and methods used in many studies. Here, indicate whether each material, system or method listed is relevant to your study. If you are not sure if a list item applies to your research, read the appropriate section before selecting a response.

Materials & experimental systems

n/a	Involved in the study
☒	☐ Antibodies
☒	☐ Eukaryotic cell lines
☒	☐ Palaeontology and archaeology
☒	☐ Animals and other organisms
☒	☐ Clinical data
☒	☐ Dual use research of concern
☒	☐ Plants

Methods

n/a	Involved in the study
☒	☐ ChIP-seq
☒	☐ Flow cytometry
☒	☐ MRI-based neuroimaging

Plants

Seed stocks	N/A
Novel plant genotypes	N/A
Authentication	N/A
